# Isolable acetylene complexes of copper and silver†

**DOI:** 10.1039/d2sc02377f

**Published:** 2022-05-20

**Authors:** Anurag Noonikara-Poyil, Shawn G. Ridlen, Israel Fernández, H. V. Rasika Dias

**Affiliations:** Department of Chemistry and Biochemistry, The University of Texas at Arlington Arlington Texas 76019 USA dias@uta.edu; Departamento de Química Orgánica I and Centro de Innovación en Química Avanzada (ORFEO-CINQA), Facultad de Ciencias Químicas, Universidad Complutense de Madrid 28040-Madrid Spain israel@quim.ucm.es

## Abstract

Copper and silver play important roles in acetylene transformations but isolable molecules with acetylene bonded to Cu(i) and Ag(i) ions are scarce. This report describes the stabilization of π-acetylene complexes of such metal ions supported by fluorinated and non-fluorinated, pyrazole-based chelators. These Cu(i) and Ag(i) complexes were formed readily in solutions under an atmosphere of excess acetylene and the appropriate ligand supported metal precursor, and could be isolated as crystalline solids, enabling complete characterization using multiple tools including X-ray crystallography. Molecules that display κ^2^-or κ^3^-ligand coordination modes and trigonal planar or tetrahedral metal centers have been observed. Different trends in coordination shifts of the acetylenic carbon resonance were revealed by ^13^C NMR spectroscopy for the Cu(i) and Ag(i) complexes. The reduction in acetylene *ν̄*_C≡C_ due to metal ion coordination is relatively large for copper adducts. Computational tools were also used to quantitatively understand in detail the bonding situation in these species. It is found that the interaction between the transition metal fragment and the acetylene ligand is significantly stronger in the copper complexes, which is consistent with the experimental findings. The C≡C distance of these copper and silver acetylene complexes resulting from routine X-ray models suffers due to incomplete deconvolution of thermal smearing and anisotropy of the electron density in acetylene, and is shorter than expected. A method to estimate the C≡C distance of these metal complexes based on their experimental *ν̄*_C≡C_ is also presented.

## Abstract 2

Gaseous acetylene can be trapped on copper(i) and silver(i) sites supported by pyrazole-based scorpionates to produce isolable molecules for detailed investigations and the study of metal-acetylene bonding.

## Graphical abstract

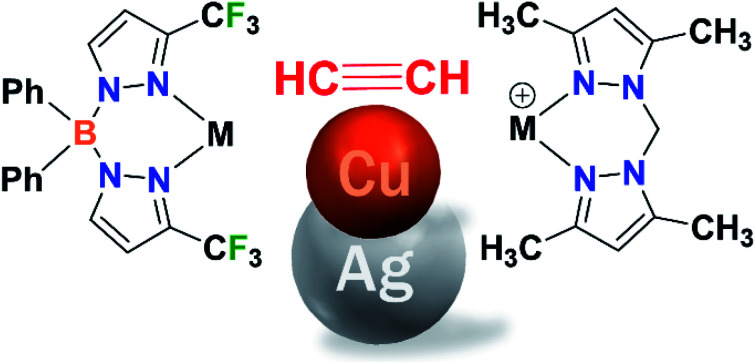

## Introduction

Acetylene (C_2_H_2_) is a useful building block in organic and industrial chemistry.^1^ It is usually obtained from coal *via* a process involving calcium carbide (which is different from the petroleum-based, other important C2-feedstock, ethylene).^1*c*,2^ However, compared to ethylene, the applications involving acetylene are somewhat challenging due to its fire and explosion risks, especially under high-pressure conditions and in purified form.^1*c*^ Furthermore, additional care must be taken when certain metals such as copper and silver are involved because they are known to form explosive acetylides and carbides with acetylene.^1*a*,3^ Nevertheless, transition metals, including copper and silver, have been utilized successfully in many acetylene transformations.^1*a*,*b*,4^ Selective semi-hydrogenation of acetylene in ethylene-rich gas streams to produce ethylene is one such application with great industrial importance, as it serves as an effective method to remove acetylene impurities in ethylene feedstocks. Silver-modified palladium is the most commonly used catalyst for this purpose.^5^ Various other silver and copper containing materials and copper complexes are also known to facilitate this process.^5*a*,6^ Silver mediated addition^7^ and carboxylation^8^ reactions of acetylene and use in acetylene sensing^9^ have been reported. Copper and/or copper salts also play diverse roles in acetylene chemistry as in the ethynylation (*e.g.*, in the 1,4-butynedione synthesis), hydrochlorination, carbonylation, cross-couplings, and azide–alkyne cycloaddition reactions, as well as vinylacetylene and cuprene synthesis.^1,10^ Acetylene has also been separated very effectively from CO_2_ using copper containing materials.^11^ The metal carbide formations noted above could be considered as “C–H activation” processes.^12^ Although limitations must be considered, the advancements stated herein show that copper and silver play an integral role in the acetylene chemistry.

**Fig. 1:**
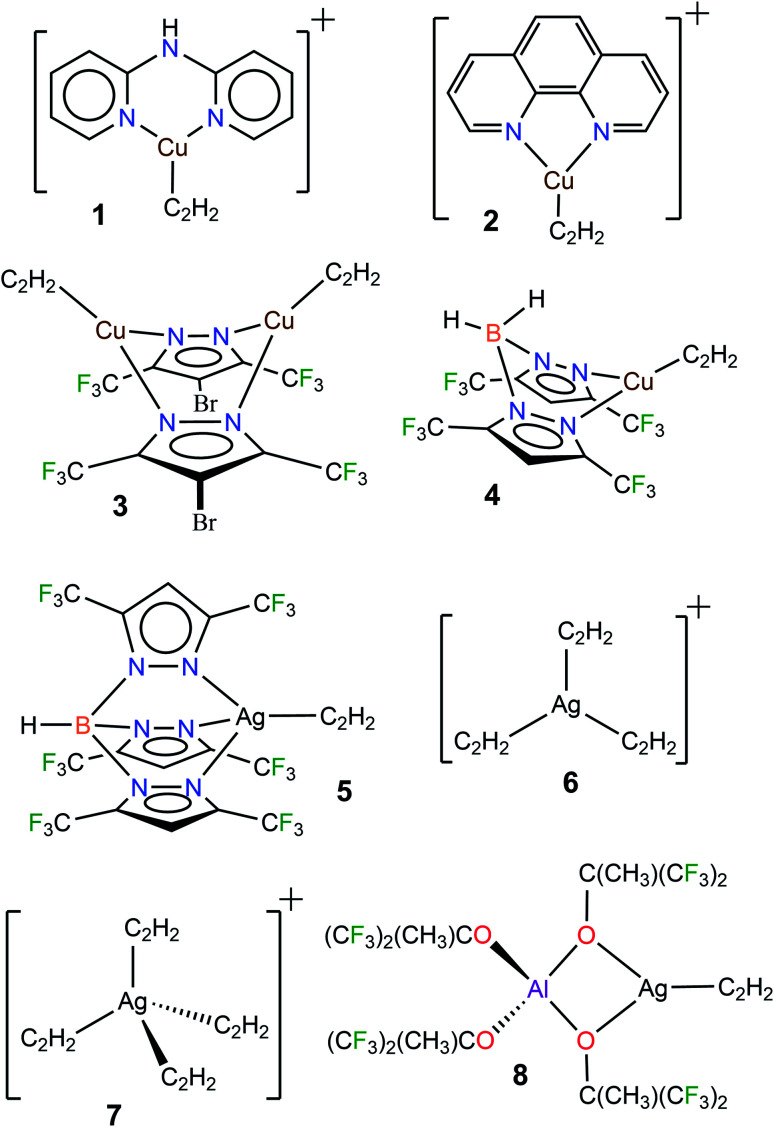
Diagram showing the structures of well-characterized copper(i) and silver(i) complexes containing η^2^-bound acetylene. Counterions [BF_4_]^−^ and [ClO_4_]^−^ of 1 and 2, and [Al(OC(CF_3_)_3_)_4_]^−^ of 6 and 7 have been omitted for clarity.

The fundamental chemistry such as structures and bonding of π-acetylene complexes of copper and silver are of significant interest because they provide useful information for the design and development of processes for separation,^13^ activation, and utilization of this important C2-feedstock chemical.^1^ However, despite over a 100 year history of coinage metal (Cu, Ag, Au) chemistry of acetylene,^3*b*,14^ and the current importance,^1*a*,4^ well-characterized molecules featuring terminal Cu(η^2^-HC≡CH) and Ag(η^2^-HC≡CH) bonds are still very limited. For example, a search of the Cambridge Structural Database^15^ revealed only four copper complexes, [Cu{NH(Py)_2_}(C_2_H_2_)][BF_4_] (1[BF_4_]),^16^ [Cu(phen)(C_2_H_2_)][ClO_4_] (2[ClO_4_]),^17^ Cu_2_(μ-[4-Br-3,5-(CF_3_)_2_Pz])_2_(C_2_H_2_)_2_ (3),^10*a*^ and [H_2_B(3,5-(CF_3_)_2_Pz)_2_]Cu(C_2_H_2_) (4),^18^ and four silver complexes [HB(3,5-(CF_3_)_2_Pz)_3_]Ag(C_2_H_2_) (5),^19^ [Ag(C_2_H_2_)_3_][Al(OC(CF_3_)_3_)_4_] (6[Al(OC(CF_3_)_3_)_4_]),^20^ [Ag(C_2_H_2_)_4_][Al(OC(CF_3_)_3_)_4_] (7[Al(OC(CF_3_)_3_)_4_]),^20^ and [Al(OC(CH_3_)(CF_3_)_2_)_4_]Ag(C_2_H_2_) (8)^20^ containing terminal M(η^2^-HC≡CH) bonds (Fig. 1, M = Cu, Ag). It is also noteworthy that these few isolable species differ in terms of charge, coordination number and/or supporting ligands, and therefore are of limited use for comparisons. Even the gas-phase studies of Cu and Ag acetylene species are quite limited.^21^ This scarcity is perhaps due to challenges such as facile loss of coordinated acetylene, metal acetylide and carbide formation, and the potential safety hazards associated with this work.^3^

**Fig. 2:**
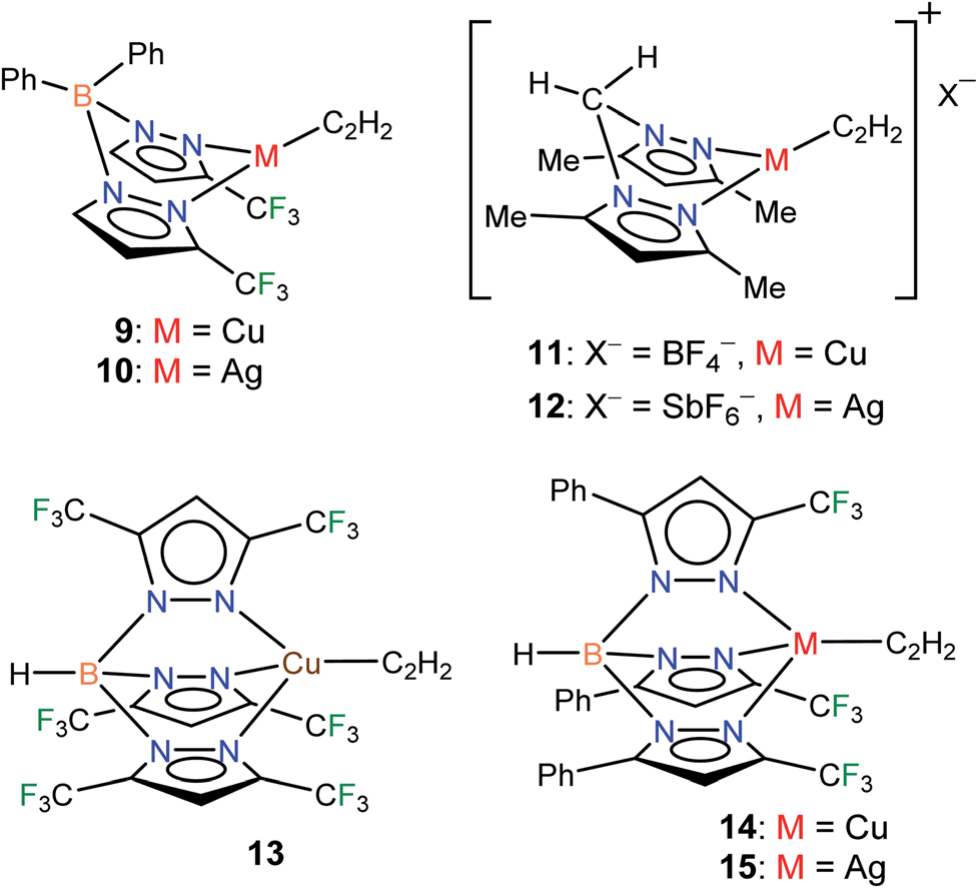
Structures of stabilized π-acetylene complexes of copper(i) and silver(i) described in this work.

Considering the importance of copper and silver in acetylene chemistry, we set out to uncover and characterize a group of molecules suitable for detailed comparisons and analysis. Herein we report the successful stabilization of several π-acetylene complexes of copper(i) and silver(i) and their spectroscopic features and X-ray crystal structures (Fig. 2). Furthermore, in this work, we demonstrate the utility of bis- and tris(pyrazolyl)borate ligands, [Ph_2_B(3-(CF_3_)Pz)_2_]^−^, [HB(3,5-(CF_3_)_2_Pz)_3_]^−^, and [HB(3-(CF_3_),5-(Ph)Pz)_3_]^−^ to stabilize neutral, and bis(pyrazolyl)methane H_2_C(3,5-(CH_3_)_2_Pz)_2_ to isolate cationic, copper and silver acetylene complexes. A complete, comparative analysis of the bonding situation of these metal-acetylene complexes using density functional theory (DFT) calculations is also presented.

## Results and discussion

### Synthesis of copper and silver complexes of acetylene supported by scorpionates

**Scheme 1:**
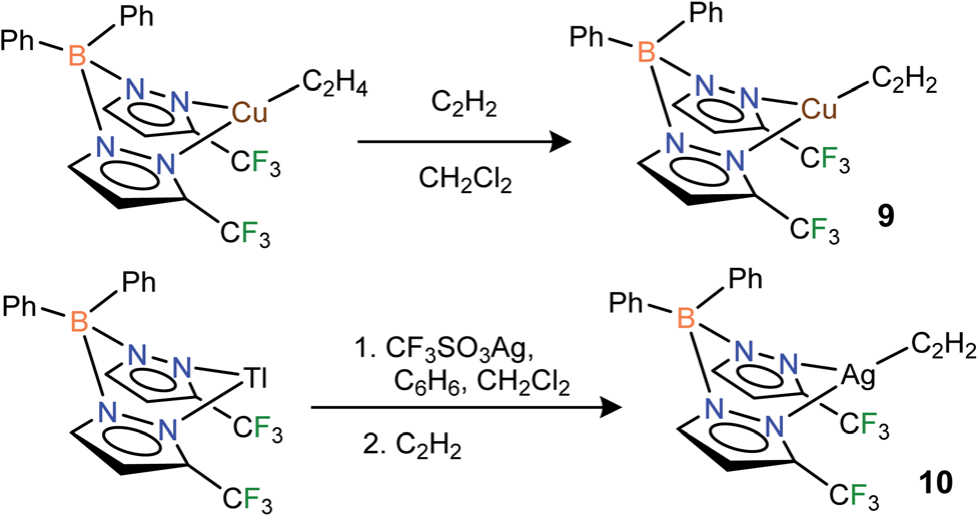
Structures and synthetic routes to bis(pyrazolyl)borate complexes [Ph_2_B(3-(CF_3_)Pz)_2_]Cu(C_2_H_2_) (9), [Ph_2_B(3-(CF_3_)Pz)_2_]Ag(C_2_H_2_)(10).

Fluorinated scorpionates^22^ have been quite useful in producing isolable molecules of reactive and/or labile organometallic fragments, including ethylene complexes of coinage metal ions.^23^ Thus, we turned to the same family of supporting ligands as the starting point for this challenging endeavor to stabilize molecules with Cu(η^2^-HC≡CH) and Ag(η^2^-HC≡CH) bonds. Indeed, the fluorinated bis(pyrazolyl)borate copper(i) complex [Ph_2_B(3-(CF_3_)Pz)_2_]Cu(C_2_H_4_)^23*g*^ undergoes a displacement reaction quite readily with purified acetylene (∼1 atm)^1*a*,24^ in CH_2_Cl_2_, affording [Ph_2_B(3-(CF_3_)Pz)_2_]Cu(C_2_H_2_) (9) as a white solid in 98% yield (Scheme 1). The related silver(i) complex [Ph_2_B(3-(CF_3_)Pz)_2_]Ag(C_2_H_2_) (10) has been synthesized from [Ph_2_B(3-(CF_3_)Pz)_2_]Tl,^23*g*^ silver triflate and purified C_2_H_2_ (∼1 atm) and isolated as a white powder in 52% yield. Synthesis of the silver–acetylene complex supported by [H_2_B(3,5-(CF_3_)_2_Pz)_2_]^−^ was also attempted but the target product could not be isolated due to the decomposition in solution, likely caused by the reduction of Ag(i) to silver metal by the BH_2_ group. Note however that the copper complex [H_2_B(3,5-(CF_3_)_2_Pz)_2_]Cu(C_2_H_2_) (4) is isolable.^18^

**Scheme 2:**
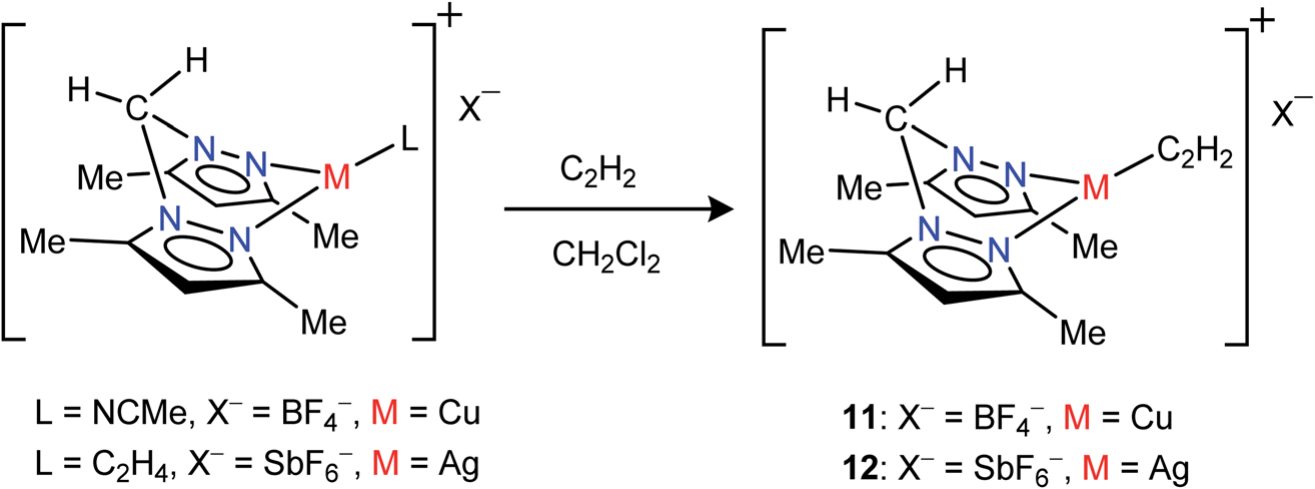
Structures and synthetic routes to bis(pyrazolyl)methane complexes [{H_2_C(3,5-(CH_3_)_2_Pz)_2_}Cu(C_2_H_2_)][BF_4_] (11) and [{H_2_C(3,5-(CH_3_)_2_Pz)_2_}Ag(C_2_H_2_)][SbF_6_](12).

Neutral bis(pyrazolyl)methane donors are close relatives of the anionic, bis(pyrazolyl)borates.^22*b*,23*a*^ We discovered that even the non-fluorinated and easily accessible H_2_C(3,5-(CH_3_)_2_Pz)_2_ can be employed to stabilize copper and silver acetylene complexes successfully. For example, the cationic, bis(pyrazolyl)methane copper(i) complex [{H_2_C(3,5-(CH_3_)_2_Pz)_2_}Cu(C_2_H_2_)][BF_4_] (11) can be obtained as a white solid in 97% yield by treating the copper(i) acetonitrile complex [{H_2_C(3,5-(CH_3_)_2_Pz)_2_}Cu(CH_3_CN)][BF_4_]^23*i*^ with purified acetylene in CH_2_Cl_2_ (Scheme 2). The bis(pyrazolyl)methane silver(i) complex [{H_2_C(3,5-(CH_3_)_2_Pz)_2_}Ag(C_2_H_2_)][SbF_6_] (12) was synthesized from [{H_2_C(3,5-(CH_3_)_2_Pz)_2_}Ag(C_2_H_4_)][SbF_6_]^23*i*^ by displacing ethylene with acetylene in CH_2_Cl_2_ and isolated in 93% yield as a white powder.

**Scheme 3:**
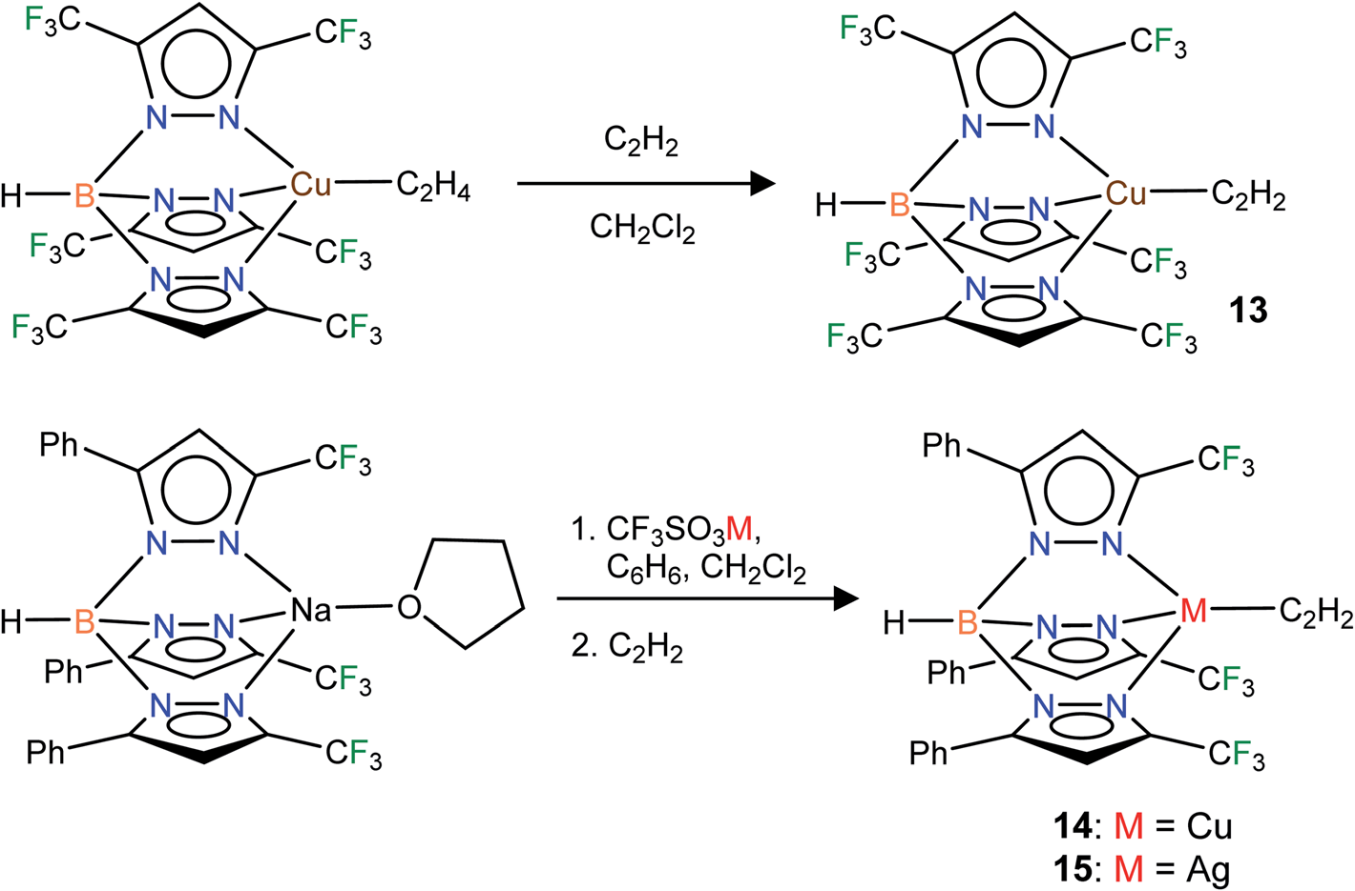
Structures and synthetic routes to tris(pyrazolyl)borato copper and silver complexes, [HB(3,5-(CF_3_)_2_Pz)_3_]Cu(C_2_H_2_) (13), [HB(3-(CF_3_),5-(Ph)Pz)_3_]Cu(C_2_H_2_) (14) and [HB(3-(CF_3_),5-(Ph)Pz)_3_]Ag(C_2_H_2_) (15).

In addition to the 3-coordinate species described above, we also wanted to isolate 4-coordinate Cu(i) and Ag(i) acetylene complexes using tridentate chelators and probe their chemistry. As apparent from the list of molecules illustrated in Fig. 1, such species are the minority. We found that [HB(3,5-(CF_3_)_2_Pz)_3_]Cu(C_2_H_2_) (13) supported by a highly fluorinated tris(pyrazolyl)borate can be obtained in essentially quantitative yield from the corresponding ethylene complex [HB(3,5-(CF_3_)_2_Pz)_3_]Cu(C_2_H_4_)^23*c*^ (Scheme 3). It is the copper analog of the silver-η^2^-acetylene complex [HB(3,5-(CF_3_)_2_Pz)_3_]Ag(C_2_H_2_) (5).^19^ Furthermore, the copper(i) and silver(i) complexes [HB(3-(CF_3_),5-(Ph)Pz)_3_]Cu(C_2_H_2_) (14) and [HB(3-(CF_3_),5-(Ph)Pz)_3_]Ag(C_2_H_2_) (15) supported by a relatively less fluorinated tris(pyrazolyl)borate have been synthesized starting from the ligand sodium salt [HB(3-(CF_3_),5-(Ph)Pz)_3_]Na(THF)^25^ and the corresponding metal triflate and acetylene (Scheme 3), and isolated as solids in 69% and 71% yield, respectively. Molecular pairs such as 13, 14 and 5, 15 serve as ideal systems to investigate ligand effects on spectroscopic and structural features of the M(η^2^-HC≡CH) group. We have also attempted the synthesis of gold(i)-acetylene analogs using several supporting ligands. However, no isolable molecules could be obtained thus far due to facile decomposition.

Copper and silver complexes 9–15 are thermally stable solids at room temperature under an acetylene atmosphere. They can be handled, even in air, for brief periods (*e.g.*, to prepare NMR samples) without signs of decomposition. Solid samples of 10–12 show some acetylene loss under nitrogen after several hours (Table 1) but lose acetylene rapidly and completely under reduced pressure. They all however retain the intact scorpionate ligands even after the acetylene loss, as evident from the NMR data. In fact, except in 10, the original acetylene complexes can be regenerated by exposing acetylene-free solids to C_2_H_2_ gas in solution. Compound 10 forms a somewhat insoluble solid (presumably a polymeric material generated as observed with {[PhB(3-(CF_3_)Pz)_3_]Ag}_∞_)^26^ with the loss of C_2_H_2_, impeding the reverse, acetylene fixing process. The tris(pyrazolyl)borate complexes 13, 14 and 5 are notably stable copper and silver acetylene complexes under a variety of conditions. The ^1^H NMR data of 9 and 11–15 taken immediately after preparing solutions in CDCl_3_ show the expected products without signs of decomposition or C_2_H_2_ loss (while compound 10 indicates some C_2_H_2_ loss). Additional details on the stability of copper and silver acetylene complex pairs in the solid form and solution (CDCl_3_) at room temperature are presented in Table 1 (and ESI†).

**Table 1:** Stability of copper and silver acetylene complexes under different conditions at ambient temperature. See the ESI for additional details

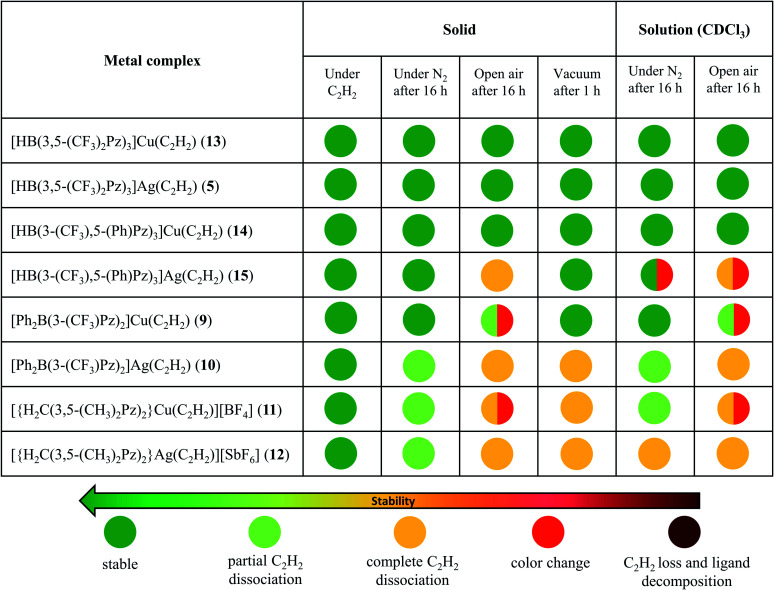

Table 2 shows available, albeit limited, key ^1^H and ^13^C NMR data and C≡C stretch of structurally characterized copper and silver complexes 1–8 and those of the newly synthesized complexes 9–15. A copper complex Cu_4_(μ-[3,5-(CF_3_)_2_Pz])_4_(μ-HC≡CH)_2_ containing a μ_2_-η^2^,η^2^-(HC≡CH) (which is a bridging acetylene)^10*a*^ has also been included for comparisons. The ^1^H NMR spectra of copper(i) complexes in general show a larger downfield shift (shift towards the typical alkene region) of acetylenic proton signal from the free acetylene resonance, whereas the silver analog shows only a smaller congruent shift. For example, the room temperature ^1^H NMR spectrum of 9 in CDCl_3_ exhibited the acetylenic proton resonance at *δ* 4.22 ppm which is a significant downfield shift relative to the corresponding signal of the free acetylene (*δ* 1.91 ppm). Furthermore, the acetylenic protons of cationic 11 in (CD_3_)_2_CO were observed at *δ* 5.14 ppm. This also indicates that the acetylene on [{H_2_C(3,5-(CH_3_)_2_Pz)_2_}Cu]^+^ does not get displaced by acetone. The corresponding resonance of silver complexes 10 and 12 was observed at *δ* 2.13 and 2.25 ppm, respectively, closer to the free acetylene peak position. The ^13^C NMR resonances of the copper(i) and silver(i) coordinated acetylene carbons are interesting in the sense that they show shifts in opposite directions from that of the free acetylene carbon signal (Table 2). For example, ^13^C NMR resonance of the acetylenic carbons of free acetylene, copper complex 9 and silver complex 10 has been observed at *δ* 72.0, 78.7, and 70.9 ppm, respectively. Note that there are other d-block metal-acetylene complexes with comparatively larger shifts in acetylenic proton and carbon signals.^27^ For example, (Ph_3_P)_2_Ni(HC≡CH)^28^ complex involving the significantly better backbonding Ni(0) displays its proton and carbon signals for the nickel-bound η^2^-(HC≡CH) in ^1^H and ^13^C NMR spectra at *δ* 6.41, 122 ppm, respectively.

**Table 2:** Selected peaks from ^1^H, ^13^C NMR and vibrational spectra for copper(i) and silver(i) complexes and the chemical shift (Δ*δ*) from free acetylene (Δ*δ* = *δ* (metal complex) – *δ* (free acetylene) and Δ*ν̄*_C≡C_ = *ν̄*_C≡C_ (metal complex) – *ν̄*_C≡C_ (free acetylene))^a^

Compound	Raman/IR (cm^−1^) (C≡C)	Δ*ν̄*_C≡C_ (cm^−1^)	^1^H NMR (ppm) (C_2_H_2_)	Δ*δ* (ppm)	^13^C{^1^H} NMR (ppm) (C≡C)	Δ*δ* (ppm)	Ref.
[Ph_2_B(3-(CF_3_)Pz)_2_]Cu(C_2_H_2_) (9)	1807	−167	4.22	2.31	78.7	6.7	This work
[HB(3-(CF_3_),5-(Ph)Pz)_3_]Cu(C_2_H_2_) (14)	1829	−145	4.66	2.75	76.5	4.5	This work
[HB(3,5-(CF_3_)_2_Pz)_3_]Cu(C_2_H_2_) (13)	1845	−129	4.50	2.59	75.8	3.8	This work
[{H_2_C(3,5-(CH_3_)_2_Pz)_2_}Cu(C_2_H_2_)][BF_4_] (11)	1812	−162	5.14^b^	2.73	79.5^b^	5.9	This work
[H_2_B(3,5-(CF_3_)_2_Pz)_2_]Cu(C_2_H_2_) (4)	1819	−155	4.70	2.79	80.2	8.2	18
Cu_2_(μ-[4-Br-3,5-(CF_3_)_2_Pz])_2_(C_2_H_2_)_2_ (3)	1811	−163	4.75^c^^,^^e^	2.95	—	—	10a
[Cu{NH(Py)_2_}(C_2_H_2_)][BF_4_] (1[BF_4_])	1795	−179	5.59^b^	3.18	—	—	16
[Cu(phen)(C_2_H_2_)][ClO_4_] (2[ClO_4_])	1800	−174	—	—	—	—	17
Cu_4_(μ-[3,5-(CF_3_)_2_Pz])_4_(μ-C_2_H_2_)_2_^d^	1638	−336	6.16	4.25	79.2	7.2	10a
[Ph_2_B(3-(CF_3_)Pz)_2_]Ag(C_2_H_2_) (10)	—	—	2.13	0.22	70.9	−1.1	This work
[HB(3-(CF_3_),5-(Ph)Pz)_3_]Ag(C_2_H_2_) (15)	1895	−79	3.59^c^	1.79	66.7^c^	−5.2	This work
[HB(3,5-(CF_3_)_2_Pz)_3_]Ag(C_2_H_2_) (5)	1905	−69	3.48	1.57	66.3	−5.6	19
[{H_2_C(3,5-(CH_3_)_2_Pz)_2_}Ag(C_2_H_2_)][SbF_6_] (12)	—	—	2.25^c^	0.45	71.7^c^	−0.2	This work
[Al(OC(CH_3_)(CF_3_)_2_)_4_]Ag(C_2_H_2_) (8)	1914	−60	3.03^c^	1.23	69.7^c^	−2.3	20
[Ag(C_2_H_2_)_3_][Al(OC(CF_3_)_3_)_4_] (6[Al(OC(CF_3_)_3_)_4_])	1925	−49	2.87^c^	1.07	72.8^c^	0.9	20
[Ag(C_2_H_2_)_4_][Al(OC(CF_3_)_3_)_4_] (7[Al(OC(CF_3_)_3_)_4_])	1940	−34	2.66^c^	0.86	72.7^c^	0.8	20
Free C_2_H_2_	1974	0	1.91 (CDCl_3_)	0	72.0 (CDCl_3_)	0	This work, 20 and 29
			2.41 ((CD_3_)_2_CO)		73.6 ((CD_3_)_2_CO)		
			1.80 (CD_2_Cl_2_)		71.9 (CD_2_Cl_2_)		

a Some NMR data in solvents other than CDCl_3_.

b (CD_3_)_2_CO.

c CD_2_Cl_2_.

d A copper complex featuring a bridging acetylene ligand (serving as a formally 4e-donor) for comparisons.

e NMR data collected at −70 °C.

The Raman and IR data of the η^2^-(HC≡CH) copper(i) complexes show a reduction of C≡C stretch by over >100 cm^−1^ with an average of reduction of 160 cm^−1^ relative to that of the free acetylene stretch observed at 1974 cm^−1^.^29^ This implies a weakening of the C≡C bond due to σ/π-interaction between copper(i) and acetylene (both components reduce the CC bond order) in terms of the Dewar–Chatt–Duncanson picture.^30^ However, the reduction in wavenumber is not as high as that observed with Cu_4_(μ-[3,5-(CF_3_)_2_Pz])_4_(μ-HC≡CH)_2_ containing bridging acetylenes, which is understandable. Furthermore, ligand effects on *ν̄*_C≡C_ are also apparent from some related complexes in which weakly donating ligand support on copper(i) produces molecules that display relatively higher HC≡CH stretch, *e.g.*, 9*vs.*4 or 14*vs.*13. Compared to Cu(i), the effect of Ag(i) on η^2^-(HC≡CH) is relatively small as evident from a significantly smaller reduction (average 60 cm^−1^ reduction from the corresponding stretch of the free C_2_H_2_). This is in agreement with silver(i) being a weaker σ-bonding and π-backbonding metal ion compared to copper(i) atom (*e.g.*, d^10^ → d^10^s^1^ electron affinities of Cu(i) and Ag(i) ions are 7.72 and 7.57 eV, in terms of energy released, respectively, and d^10^ → d^9^p^1^ promotional energies of Cu(i) and Ag(i) are 8.25 and 9.94 eV, respectively).^31^ A much more detailed analysis of metal-acetylene bonding using DFT is also given below. Unfortunately, the background fluorescence and acetylene loss prevented the observation of the *ν̄*_C≡C_ band of some silver complexes reported in this manuscript.

### X-ray crystal structures of copper and silver acetylene complexes supported by scorpionates

**Fig. 3:**
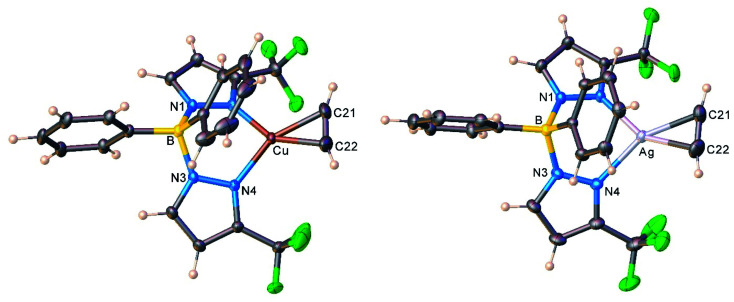
Molecular structures of [Ph_2_B(3-(CF_3_)Pz)_2_]Cu(C_2_H_2_) (9, left) and [Ph_2_B(3-(CF_3_)Pz)_2_]Ag(C_2_H_2_) (10, right).

The copper and silver acetylene complexes, [Ph_2_B(3-(CF_3_)Pz)_2_]Cu(C_2_H_2_) (9) and [Ph_2_B(3-(CF_3_)Pz)_2_]Ag(C_2_H_2_) (10) afforded excellent single crystals and were characterized by X-ray crystallography. Fig. 3 depicts the molecular structures of these molecules. They are three-coordinate, trigonal planar metal complexes with *κ*^2^-bound [Ph_2_B(3-(CF_3_)Pz)_2_]^−^ ligands. The acetylene ligand coordinates to the metal in a familiar η^2^-fashion. The M(NN)_2_B core (M = Cu, Ag) adopts a boat conformation. These molecules feature a flanking phenyl group above the metal-acetylene moiety with closest M⋯C(phenyl) separations of 3.01 and 2.88 Å in the Cu and Ag complex, respectively. Although these atoms are within the Bondi's van der Waals separation distances of 3.10 and 3.42 Å (or 4.15 and 4.30 Å proposed by Álvarez)^32^ for Cu⋯C and Ag⋯C,^33^ any interactions present between the metal and phenyl group do not affect the trigonal planar geometry at the metal (see also the computational section, below).

**Fig. 4:**
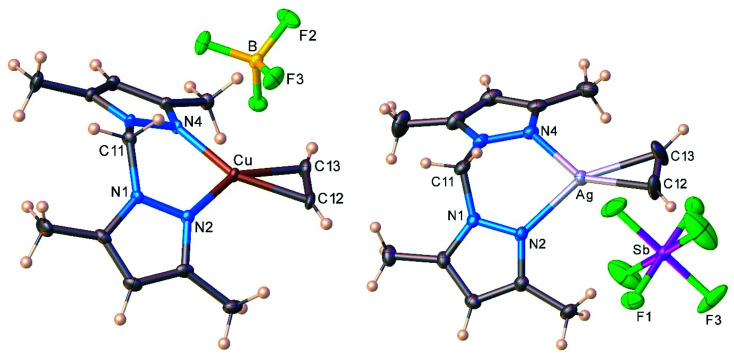
Molecular structures of [{H_2_C(3,5-(CH_3_)_2_Pz)_2_}Cu(C_2_H_2_)][BF_4_] (11, left) and [{H_2_C(3,5-(CH_3_)_2_Pz)_2_}Ag(C_2_H_2_)][SbF_6_] (12, right).

The molecular structures of the cationic acetylene complexes [{H_2_C(3,5-(CH_3_)_2_Pz)_2_}Cu(C_2_H_2_)][BF_4_] (11) and [{H_2_C(3,5-(CH_3_)_2_Pz)_2_}Ag(C_2_H_2_)][SbF_6_] (12) involving a bis(pyrazolyl)methane ligand are illustrated in Fig. 4. The M(NN)_2_C core of the bis(pyrazolyl)methane ligand in 11 and 12 adopts a flat boat conformation. The key difference between bis(pyrazolyl)borate and bis(pyrazolyl)methane backbone shapes is reflected in the larger MN⋯NM separation of the pyrazolyl groups of the latter (see ESI Fig. S43†).

As evident from the data presented in Table 3, Cu–N and Cu–C distances of 9 and 11 are shorter than the related separations involving silver in 10 and 12, which is expected as Ag is the largest metal of the coinage metal triad.^34^ Consequently, the C–Cu–C and N–Cu–N angles are significantly larger than those parameters involving silver. The acetylene ligands of 9 and 10 are essentially coplanar with the N–M–N plane (M = Cu, Ag; silver complex shows the larger twist angle of 3° but it is still minor). This parallel orientation of NMN and CMC planes is the best for maximizing metal-(η^2^-ligand) backbonding interactions, rather than the orthogonal conformation.^35^ However in contrast to 9 and 10, C–M–C and N–M–N planes of 11 and 12 deviate somewhat from co-planarity with the copper and silver adducts showing 8.8° and 11.9° inter-planar twist angles. Crystal packing diagrams indicate that one of the fluorine atoms of [BF_4_]^−^ in 11 sits near Cu at 2.8842(12) Å, while two fluorine atoms of two different [SbF_6_]^−^ counter-ions are closer to the silver center (at 3.364(4), 3.439(3) Å) of 12. These separations are longer than the Bondi's van der Waals contact separation of F with Cu (2.87 Å) and Ag (3.19 Å), and do not distort the trigonal planar geometry at copper and silver, as evident from the sum of angles at M (M = Cu, Ag) of 360°.

**Table 3:** Selected bond lengths and angles of three-coordinate copper and silver acetylene complexes and those of several related ethylene complexes for comparison. The CC distance of free acetylene is 1.20286(3) Å based on gas-phase experimental data^36^ and 1.193(6) Å from neutron diffraction data on solid acetylene.^37^ The CC bond distance (*r*(spec)) estimated from CC stretch is given in *italics* for metal acetylene complexes with *ν̄*_C≡C_ data (see Table 2 and eqn. (1)). The CC distance of free ethylene for comparison is 1.3305(10) Å from gas phase data and 1.313 Å from X-ray data^38^

Compound	π-CC (Å)	C–M–C (°)	N–M–N (°)	M–N (Å)	C–M (Å)	CN at M^a^	Ref.
[Ph_2_B(3-(CF_3_)Pz)_2_]Cu(C_2_H_2_) (9)	1.217(3)	36.17(8)	95.51(4)	1.9714(10)	1.9629(14)	3	This work
	*1.236*			1.9697(10)	1.9567(15)		
[{H_2_C(3,5-(CH_3_)_2_Pz)_2_}Cu(C_2_H_2_)][BF_4_] (11)	1.203(4)	35.55(13)	97.14(9)	1.978(2)	1.970(3)	3	This work
	*1.235*			1.977(2)	1.971(3)		
[Ph_2_B(3-(CF_3_)Pz)_2_]Ag(C_2_H_2_) (10)	1.193(3)	30.63(8)	82.76(5)	2.2665(12)	2.2653(19)	3	This work
				2.2415(14)	2.2531(19)		
[{H_2_C(3,5-(CH_3_)_2_Pz)_2_}Ag(C_2_H_2_)][SbF_6_] (12)	1.203(5)	31.10(14)	88.66(9)	2.220(2)	2.251(3)	3	This work
				2.235(2)	2.237(4)		
[HB(3,5-(CF_3_)_2_Pz)_3_]Cu(C_2_H_2_) (13)	1.134(7)	33.16(19)	90.17(10)	2.0466(17)	1.986(3)	4	This work
	*1.228*		88.25(7)	2.0466(17)	1.986(3)		
			88.25(7)	2.179(3)			
[HB(3,5-(CF_3_)_2_Pz)_3_]Ag(C_2_H_2_) (5)	1.143(14)	28.9(4)	80.99(11)	2.293(4)	2.293(4),	4	19
	*1.216*		80.99(11)	2.347(3)	2.293(4)		
			81.1(2)	2.364(4)			

[Ph_2_B(3-(CF_3_)Pz)_2_]Cu(C_2_H_4_)^b^	1.369(2)	39.59(6)	93.05(4)	1.9937(10)	2.0199(13)	3	23g
	1.353(2)	39.00(6)	92.30(4)	1.9870(10)	2.0225(13)		
				1.9980(10)	2.0307(14)		
				2.0075(10)	2.0230(15)		
[{H_2_C(3,5-(CH_3_)_2_Pz)_2_}Cu(C_2_H_4_)][*n*-BuBF_3_]	1.361(2)	39.44(6)	94.45(4)	1.9885(11)	2.0153(13)	3	23i
				1.9896(11)	2.0181(13)		
[{H_2_C(3,5-(CH_3_)_2_Pz)_2_}Ag(C_2_H_4_)][SbF_6_]	1.350(5)	34.96(12)	88.96(9)	2.223(2)	2.243(3)	3	23i
				2.232(2)	2.253(3)		
[{H_2_C(3,5-(CF_3_)_2_Pz)_2_}Ag(C_2_H_4_)][SbF_6_]^b^	1.340(4)	33.67(11)	86.44(6)	2.3306(18)	2.309(3)	3	23i
	1.340(4)	33.69(11)	86.49(6)	2.3328(18)	2.319(3)		
				2.3330(18)	2.312(3)		
				2.3293(18)	2.313(3)		

a Coordination number at M.

b Two molecules in the asymmetric unit.

Interestingly, metrical parameters such as Cu–N and Cu–C distances and N–Cu–N and C–Cu–C angles involving the copper center are quite similar between the cationic [{H_2_C(3,5-(CH_3_)_2_Pz)_2_}Cu(C_2_H_2_)][BF_4_] (11) and the neutral complexes [Ph_2_B(3-(CF_3_)Pz)_2_]Cu(C_2_H_2_) (9). The [{H_2_C(3,5-(CH_3_)_2_Pz)_2_}Ag(C_2_H_2_)][SbF_6_] (12) and [Ph_2_B(3-(CF_3_)Pz)_2_]Ag(C_2_H_2_) (10) also show analogous features at silver. The anionic but weakly coordinating ligand [Ph_2_B(3-(CF_3_)Pz)_2_]^−^ therefore appears to produce the same net result as the neutral and electron-rich H_2_C(3,5-(CH_3_)_2_Pz)_2_ on the bond distances and angles associated with copper(i) or silver(i).

**Fig. 5:**
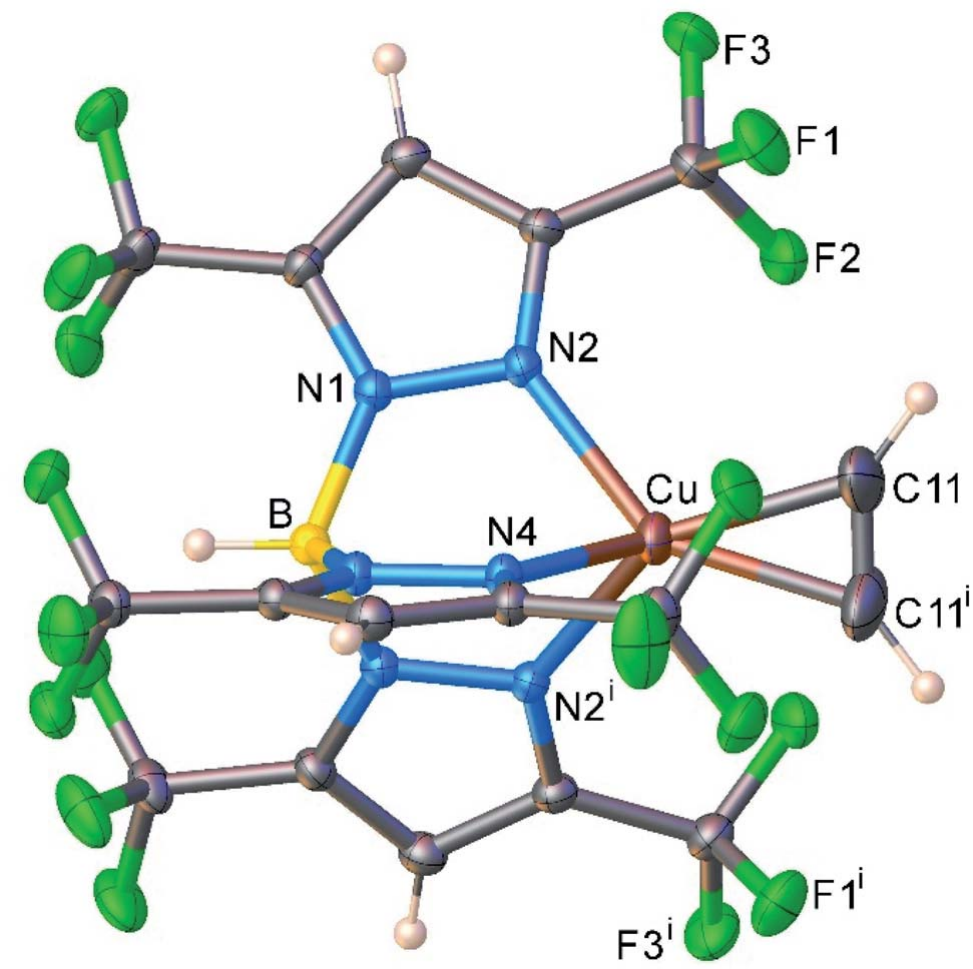
Molecular structure of [HB(3,5-(CF_3_)_2_Pz)_3_]Cu(C_2_H_2_) (13).

We also managed to characterize [HB(3,5-(CF_3_)_2_Pz)_3_]Cu(C_2_H_2_) (13) that has a highly fluorinated tris(pyrazolyl)borate supporting ligand, [HB(3,5-(CF_3_)_2_Pz)_3_]^−^ using single-crystal X-ray crystallography (Fig. 5). Interestingly, 13 is the first four-coordinate, structurally authenticated Cu(η^2^-HC≡CH) complex. It has a tetrahedral metal site. The copper atom and the centroid of the acetylene group sit on a crystallographic mirror plane. Basic structural features are similar between these copper(i) complexes and the analogous [HB(3,5-(CF_3_)_2_Pz)_3_]Ag(C_2_H_2_) (5), but as expected 13 has relatively shorter M–N and M–C distances relative to those of 5 with the larger metal ion.

**Fig. 6:**
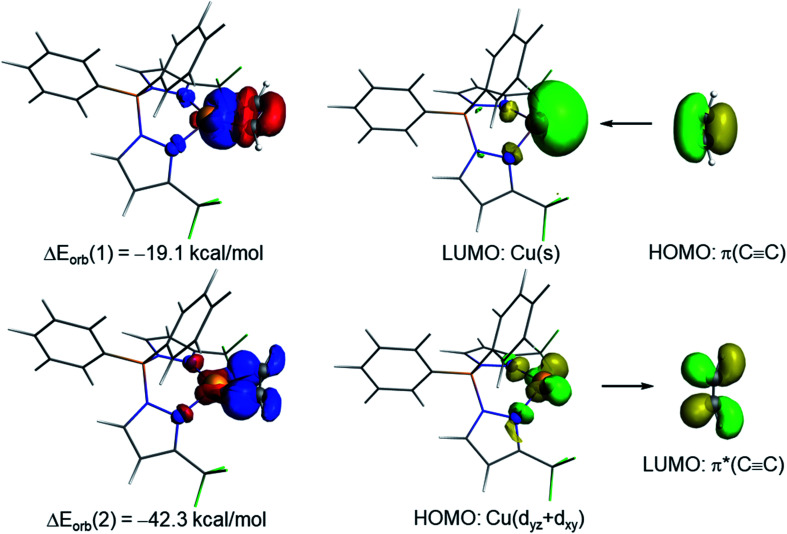
Deformation densities and the associated molecular orbitals of the dominant orbital interactions Δ*E*_orb_(1) and Δ*E*_orb_(2) in complex [Ph_2_B(3-(CF_3_)Pz)_2_]Cu(C_2_H_2_) (9). The color code used to represent the flow of charge is red → blue.

The molecular structures of [HB(3-(CF_3_),5-(Ph)Pz)_3_]Cu(C_2_H_2_) (14) and [HB(3-(CF_3_),5-(Ph)Pz)_3_]Ag(C_2_H_2_) (15) have also been investigated using X-ray crystallography (see ESI†). Unfortunately, the M(η^2^-HC≡CH) moieties of these molecules suffer significant positional disorder and therefore are not suitable for the analysis of metrical parameters. We have also observed a similar disorder in copper and silver ethylene complexes of the analogous tripodal scorpionates.^23*c*,39^ These molecules possess pockets that allow significant degrees of free motion for the small π-ligands bonded to the metal sites. Nevertheless, basic structural features and atom connectivities of 14 and 15 are clear and indicate the presence of η^2^-(HC≡CH) moieties, consistent with the spectroscopic data, and tetrahedral metal sites.

As noted above, X-ray crystallographic data on a limited number of copper(i) and silver(i) acetylene complexes are available for comparison. The C≡C bond distance of those compounds and the five uncovered in this work (Table 3 and ESI Table S19†) range from 1.092(7) Å in 7[Al(OC(CF_3_)_3_)_4_]^20^ to 1.227(4) Å in Cu_2_(μ-[4-Br-3,5-(CF_3_)_2_Pz])_2_(C_2_H_2_)_2_ (3).^10*a*^ The C≡C bond distance of Cu(i) and Ag(i) bound acetylene complexes is expected to be longer than that of the free acetylene (which is 1.20286(3) Å based on gas-phase experimental data^36^ and 1.193(6) Å from neutron diffraction data on solid acetylene),^37^ as both the σ-donation and π-backdonation interactions between the metal and acetylene causes a reduction in CC bond-order and a lengthening of the C≡C bond distance relative to that of the free acetylene. The Raman and IR data (Table 2) also support this expectation. However, most of the metal-bound C≡C bond distances of these silver and copper acetylene complexes resulting from X-ray crystallographic studies (Table 3 and ESI Table S19†) are lower than that of the free ligand. As Krossing, Scherer and co-workers have pointed out, this apparent contradiction is a result of systematic errors associated with the measurement.^20^ In small molecules such as acetylene involving multiple covalent bonds between light atoms, libration effects, incomplete deconvolution of thermal smearing and anisotropy of the electron density tend to produce bond distances that are too short from standard X-ray models.^20,40^ Such effects can be minimized by collecting data closer to absolute zero temperature and to very high angles (*e.g.*, 2*θ* = 100°).^41^ For example, the collection and analysis of the very high-resolution X-ray diffraction data of [Al(OC(CH_3_)(CF_3_)_2_)_4_]Ag(C_2_H_2_) (8) (to resolution *d* = 0.476 Å) at 10 K has produced a C≡C bond distance of 1.209(1) Å,^20^ which is in good agreement with the theoretical model (1.213 Å), while the same molecule at *d* = 0.84 Å and 90 K resulted in a length that is 0.063 Å shorter at 1.146(4) Å. This also shows the relative impact of core and valence electrons on the X-ray scattering factors (*i.e.*, scattered X-rays at higher angles are relatively less affected by the valence electrons, and therefore produce more precise nuclear or core-electron positions).^42^ Although X-ray crystal structures of 9–13 reported here do not reach the resolution level of the specialized work noted above for 8, they are quite respectable (*d* (resolution) of 0.73 to 0.60 Å at 100 K) for standard X-ray crystallography. Indeed, the analysis of the data of 9-13 at lower resolution levels (*e.g.*, using *d* = 0.84 Å, 2*θ* = 50° cut-off) produced relatively shorter C≡C bond distances (see ESI, Table S20†). Minor libration effects are also evident even at 100 K based on the TLS analysis (see ESI†).^43^ Overall, due to a combination of factors noted above, acetylene C≡C bond distances based solely on routine X-ray crystallography are not suitable for discussions of metal–ligand bonding in most Cu(i) and Ag(i) complexes, and to parse out the metal and supporting ligand effects on the acetylene moiety. Furthermore, some of the C≡C bond distance changes as a result of Cu(i) and especially Ag(i) ion coordination are also expected to be small. They are often overshadowed by the relatively high estimated standard deviations (esds) associated with the measurement and are not significantly different at the 3*σ* limit of estimated standard deviations. Similar issues have been noted also with ethylene complexes, particularly those involving silver(i).^41,44^ It is however, possible to estimate the C≡C bond distances of the metal complexes utilizing changes in C≡C vibration. As noted below in the computational section, this technique produces a more realistic estimate of the C≡C bond distance for copper and silver acetylene complexes.

Table 3 also includes structural data on a select group of Cu(i) and Ag(i) η^2^-ethylene complexes. With the availability of the analogous acetylene complexes, it is now possible to make a meaningful comparison between the two families. As expected, and despite the issues noted above with CC bond distances based on routine crystallography, the metal-bound acetylene bond distances are significantly shorter than the related ethylene bond lengths. The Cu–C and Cu–N bond distances are also shorter in the copper(i) acetylene complexes compared to their ethylene analogs. Interestingly, however, Ag–N and Ag–C distances are essentially the same in the two families. It would be interesting to see if this difference holds true also for a larger dataset.

### Computational analysis of the copper and silver acetylene complexes

Density Functional Theory (DFT) calculations at the relativistic ZORA-BP86-D3/TZ2P//RI-BP86-D3/def2-TZVPP level (see computational details in the ESI†) were carried out to understand the chemical bonding between the scorpionate-M moieties and acetylene in the above-described LM-(C_2_H_2_) complexes (L = supporting ligand; M = Cu, Ag). To this end, the combination of the Energy Decomposition Analysis (EDA) and the Natural Orbitals for Chemical Valence (NOCV) methods were applied to gain a detailed quantitative insight into the interaction between the LM and C_2_H_2_ fragments. From the data in Table 4, it becomes clear that in all cases the main contribution to the interaction between the LM and C_2_H_2_ fragments comes from the electrostatic attractions (measured by the Δ*E*_elstat_ term), which represents *ca.* 60% of the total attractive contribution. This indicates that the nature of the LM–acetylene bond is markedly ionic. Despite that, the orbital interactions (measured by the Δ*E*_orb_ term) are also significant as they contribute *ca.* 35–40% to the total interaction energy. At variance, the interactions coming from dispersion forces are much less important in the description of the bonding (<5%) and can be considered negligible.

**Table 4:** Results of the EDA-NOCV calculations (ZORA-BP86-D3/TZ2P//RI-BP86-D3/def2-TZVPP level, in kcal mol^−1^) on Cu(i)- and Ag(i)-(C_2_H_2_) complexes using LM and C_2_H_2_ as fragments (L = supporting ligand)

compound	Δ*E*_int_	Δ*E*_Pauli_	Δ*E*_elstat_^a^	Δ*E*_orb_^a^	Δ*E*_orb_(1)	Δ*E*_orb_(2)	Δ*E*_rest_	Δ*E*_disp_^a^
[H_2_B(3,5-(CF_3_)_2_Pz)_2_]Cu(C_2_H_2_) (4)	−55.2	133.9	−109.5 (57.9%)	−73.4 (38.8%)	−19.5	−41.9	−12.0	−6.2 (3.3%)
[H_2_B(3,5-(CF_3_)_2_Pz)_2_]Ag(C_2_H_2_) (4-Ag)	−32.8	115.1	−93.7 (63.3%)	−50.5 (34.1%)	−16.4	−27.5	−6.6	−3.8 (2.6%)
[H_2_B(3,5-(CH_3_)_2_Pz)_2_]Cu(C_2_H_2_) (4’)	−56.5	152.3	−119.9 (57.4%)	−83.3 (39.9%)	−18.4	−53.5	−11.4	−5.6 (2.7%)
[H_2_B(3,5-(CH_3_)_2_Pz)_2_]Ag(C_2_H_2_) (4'-Ag)	−37.1	131.7	−104.0 (61.6%)	−61.8 (36.6%)	−16.8	−35.7	−9.3	−3.0 (1.8%)
[Ph_2_B(3-(CF_3_)Pz)_2_]Cu(C_2_H_2_) (9)	−55.3	136.0	−110.4 (57.7%)	−73.4 (38.4%)	−19.1	−42.3	−12.0	−7.5 (3.9%)
[Ph_2_B(3-(CF_3_)Pz)_2_]Ag(C_2_H_2_) (10)	−35.9	119.9	−96.8 (62.1%)	−54.7 (35.1%)	−17.1	−28.2	−9.4	−4.3 (2.8%)
[{H_2_C(3,5-(CH_3_)_2_Pz)_2_}Cu(C_2_H_2_)]^+^ (11+)	−57.2	131.6	−108.4 (57.4%)	−75.2 (39.8%)	−20.4	−42.4	−12.4	−5.3 (2.8%)
[{H_2_C(3,5-(CH_3_)_2_Pz)_2_}Ag(C_2_H_2_)]^+^ (12+)	−37.0	111.6	−91.6 (61.6%)	−54.1 (36.4%)	−18.9	−25.8	−9.4	−2.9 (2.0%)
[HB(3,5-(CF_3_)_2_Pz)_3_]Cu(C_2_H_2_) (13)	−48.6	124.6	−100.1 (57.8%)	−64.7 (37.4%)	−18.6	−35.5	−10.6	−8.4 (4.8%)
[HB(3,5-(CF_3_)_2_Pz)_3_]Ag(C_2_H_2_) (5)	−30.9	104.7	−84.3 (62.2%)	−46.4 (34.2%)	−16.6	−21.2	−8.8	−4.9 (3.6%)
[HB(3-(CF_3_),5-(Ph)Pz)_3_]Cu(C_2_H_2_) (14)	−48.6	127.6	−102.0 (57.9%)	−66.1 (37.5%)	−18.0	−37.5	−10.6	−8.1 (4.6%)
[HB(3-(CF_3_),5-(Ph)Pz)_3_]Ag(C_2_H_2_) (15)	−30.8	109.0	−87.1 (62.3%)	−48.0 (34.3%)	−16.1	−23.4	−8.5	−4.7 (3.4%)
[Cu(C_2_H_2_)]^+^ (16+)	−64.7	92.1	−86.8 (55.3%)	−68.4 (43.6%)	−20.7	−30.9	−16.8	−1.7 (1.1%)
[Ag(C_2_H_2_)]^+^ (17+)	−39.8	68.6	−63.2 (58.3%)	−44.5 (41.1%)	−9.4	−25.1	−10.0	−0.7 (0.6%)

a The percentage values within parenthesis give the contribution to the total attractive interactions, Δ*E*_elstat_+ Δ*E*_orb_ + Δ*E*_disp_.

The NOCV extension of the EDA method allows us to not only identify but also quantify the main orbital interactions contributing to the total Δ*E*_orb_ term. According to the NOCV method, two main donor–acceptor orbital interactions dominate the orbital interactions in these acetylene complexes. On one hand, the σ-donation from the doubly-occupied π(C≡C) molecular orbital of the acetylene ligand to the empty s atomic orbital of the transition metal (denoted as Δ*E*_orb_(1)) and, on the other hand, the backdonation from a doubly-occupied d atomic orbital of the transition metal to the vacant π*(C≡C) molecular orbital of acetylene (denoted as Δ*E*_orb_(2), see Fig. 6 for complex 9). Interestingly, our NOCV calculations indicate that, in all cases, the backdonation from the transition metal fragment is significantly stronger (*ca.* twice as strong) than the donation from the acetylene ligand (Δ*E*_orb_(2) > Δ*E*_orb_(1)), regardless of the transition metal and the supporting ligand. In addition, our EDA-NOCV calculations confirm that both orbital interactions are stronger (in particular, the LM→ π*(C≡C) backdonation) in the Cu(i)-complexes as compared to their Ag(i)-analogues, which is in agreement with the above-commented weaker σ-bonding and π-backbonding ability of Ag(i) as compared to copper(i).^31^ Despite that, the bonding situation in these acetylene complexes can be safely described in terms of the Dewar–Chatt–Duncanson model involving two donor–acceptor interactions (σ-donation from the acetylene ligand and π-backdonation from the transition metal fragment). Note that the acetylene π/π*-orbitals perpendicular to the MC2 plane form only relatively weaker interactions with the transition metal fragment in these scorpionate ligand supported copper and silver complexes.

**Fig. 7:**
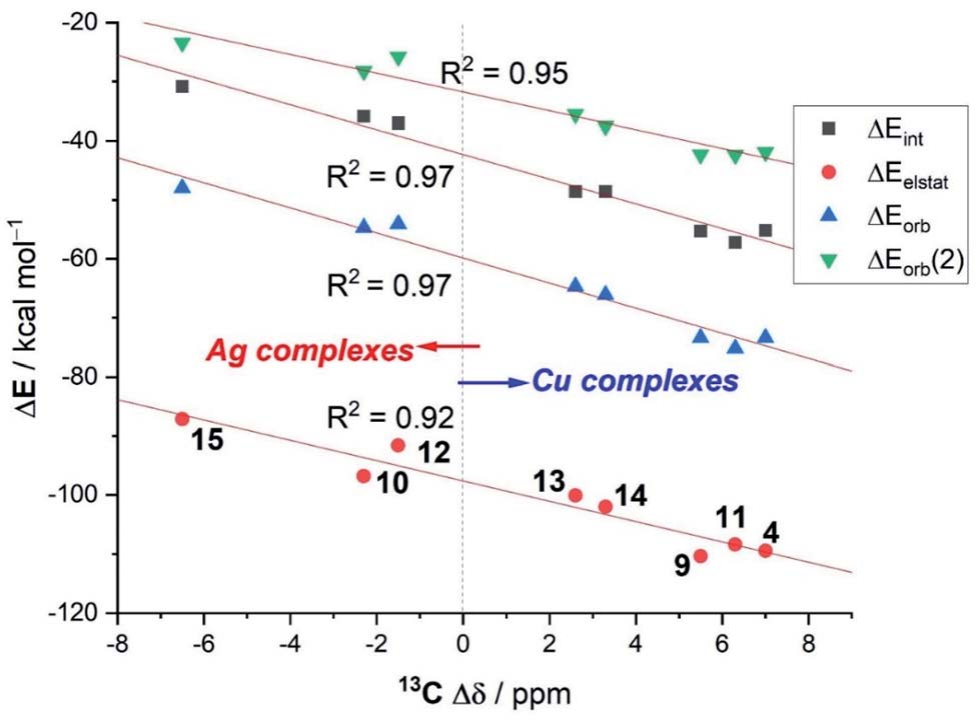
Correlations between the experimental ^13^C-NMR shifts of the acetylene carbon atom in LM–(C_2_H_2_) complexes with respect to free acetylene (Δ*δ*) *versus* the computed EDA-NOCV energy terms.

Interesting trends emerge from a closer inspection of the data gathered in Table 4. First, when comparing the copper complexes with their silver counterparts, it is found that, regardless of the supporting ligand, the interaction between the transition metal fragment and the acetylene ligand is clearly stronger in the corresponding copper complexes (Δ*E*_int_ ∼ 20 kcal mol^−1^). This is consistent with above-commented higher NMR-downfield shifts (or redshifts of the C≡C stretch), with respect to free acetylene, observed experimentally for the copper complexes. According to the data in Table 4, the enhanced interaction in the copper(i) complexes is the result of an enhancement of all the main attractive interactions (Δ*E*_elstat_, Δ*E*_orb_(1) and Δ*E*_orb_(2)) as compared to the corresponding silver(i) complexes. This finding suggests that the observed experimental shifts of these mono-acetylene complexes are closely related to the computed interaction energies (as well as their main energy contributors). To our delight, we found that indeed good linear correlations are obtained when plotting these experimental values *versus* not only the computed total interaction energies (Δ*E*_int_) but also their main EDA-NOCV contributors (see Fig. 7 for the linear relationships involving the ^13^C-NMR shifts, Δ*δ*). From the data in Fig. 7, there appears to exist a limit defining the observed shift in the ^13^C-NMR spectra with respect to free acetylene: while complexes having a LM–(C_2_H_2_) interaction Δ*E*_int_ ≥ −40 kcal mol^−1^ lead to a positive (*i.e.*, downfield) shift with respect to free acetylene (Δ*δ* > 0 ppm), complexes exhibiting lower LM–(C_2_H_2_) interaction energies provoke the opposite (*i.e.*, upfield shift) effect (Δ*δ* < 0 ppm).

Data in Table 4 also indicates that the nature of the supporting ligand also affects the LM−(C_2_H_2_) interaction. Regardless of the involved transition metal, it is found that complexes having a bidentate bis(pyrazolyl)borate/methane supporting ligand (complexes 4, 4-Ag, 9, 10, 11+, 12+) exhibit stronger LM-(C_2_H_2_) interactions than the analogous systems having a tridentate tris(pyrazolyl)borate ligand (complexes 5, 13, 14, 15). For instance, when comparing bidentate complexes 4 or 4-Ag with their tridentate counterparts 13 or 5, it becomes clear that the weaker interaction computed for the latter complexes finds its origin in the lower electrostatic and orbital (mainly the LM → π*(C≡C) backdonation, Δ*E*_orb_(2)) interactions computed for these species. Therefore, it can be concluded that supporting ligands having a lower number of donor sites lead to stronger LM-(C_2_H_2_) interactions. This is also supported by the calculations on the naked [Cu(C_2_H_2_)]^+^ and [Ag(C_2_H_2_)]^+^ cations, which exhibit the highest Δ*E*_int_ values of their corresponding series (see Table 4). Furthermore, it is found that the replacement of bidentate fluorinated bis(pyrazolyl)borate ligand (which is anionic) by the analogous non-fluorinated and neutral bis(pyrazolyl)methane ligand provokes an almost negligible effect on the LM-(C_2_H_2_) interaction (*e.g.*, compare 4 and 11+ or 4-Ag and 12+).

We have also examined the C≡C bond distances and the C≡C stretching frequencies of the copper(i) and silver(i) complexes, computationally. The expected changes to the CC distance are especially useful considering the challenges associated with measuring this parameter precisely noted above. Table 5 shows the computed C≡C distances and the corresponding stretching frequencies for the considered Cu(i) and Ag(i)-complexes together with their Au(i)-counterparts and representative group 1 and group 10 analogues.

**Table 5:** Computed C≡C bond lengths and corresponding stretching frequencies in the Cu(i), Ag(i) and Au(i)-scorpionate complexes together with representative group 10 analogues and including group 1 complexes 4-Li and 4-Na. All data have been computed at the RI-BP86/def2-TZVPP level. For comparisons, the computed CC distance of free acetylene is 1.207 Å and the frequency is 2007 cm^−1^

compound	*r*_C≡C_/Å	*ν̄*_C≡C_/cm^−1^	Δ*ν̄*_C≡C_/cm^−1^^a^
[H_2_B(3,5-(CF_3_)_2_Pz)_2_]Cu(C_2_H_2_) (4)	1.247	1811	−196
[H_2_B(3,5-(CF_3_)_2_Pz)_2_]Ag(C_2_H_2_) (4-Ag)	1.240	1835	−172
[H_2_B(3,5-(CH_3_)_2_Pz)_2_]Cu(C_2_H_2_) (4’)	1.255	1778	−229
[H_2_B(3,5-(CH_3_)_2_Pz)_2_]Ag(C_2_H_2_) (4'-Ag)	1.248	1798	−209
[Ph_2_B(3-(CF_3_)Pz)_2_]Cu(C_2_H_2_) (9)	1.248	1808	−199
[Ph_2_B(3-(CF_3_)Pz)_2_]Ag(C_2_H_2_) (10)	1.242	1829	−178
[{H_2_C(3,5-(CH_3_)_2_Pz)_2_}Cu(C_2_H_2_)]^+^ (11+)	1.249	1806	−201
[{H_2_C(3,5-(CH_3_)_2_Pz)_2_}Ag(C_2_H_2_)]^+^ (12+)	1.240	1836	−171
[HB(3,5-(CF_3_)_2_Pz)_3_]Cu(C_2_H_2_) (13)	1.241	1841	−166
[HB(3,5-(CF_3_)_2_Pz)_3_]Ag(C_2_H_2_) (5)	1.235	1861	−146
[HB(3-(CF_3_),5-(Ph)Pz)_3_]Cu(C_2_H_2_) (14)	1.242	1833	−174
[HB(3-(CF_3_),5-(Ph)Pz)_3_]Ag(C_2_H_2_) (15)	1.237	1852	−155
[H_2_B(3,5-(CF_3_)_2_Pz)_2_]Au(C_2_H_2_) (4-Au)	1.268	1726	−280
[H_2_B(3,5-(CH_3_)_2_Pz)_2_]Au(C_2_H_2_) (4’-Au)	1.276	1695	−312
[Ph_2_B(3-(CF_3_)Pz)_2_]Au(C_2_H_2_) (9-Au)	1.269	1723	−284
[{H_2_C(3,5-(CH_3_)_2_Pz)_2_}Au(C_2_H_2_)]^+^ (11+-Au)	1.268	1729	−278
[HB(3,5-(CF_3_)_2_Pz)_3_]Au(C_2_H_2_) (13-Au)	1.265	1741	−265
[HB(3-(CF_3_),5-(Ph)Pz)_3_]Au(C_2_H_2_) (14-Au)	1.266	1737	−269
[{H_2_B(3,5-(CF_3_)_2_Pz)_2_}Ni(C_2_H_2_)]^−^ (4-Ni-)	1.287	1651	−356
[{H_2_B(3,5-(CF_3_)_2_Pz)_2_}Ni(C_2_H_2_)]^−^ (4-Pd-)	1.285	1656	−351
[{H_2_B(3,5-(CF_3_)_2_Pz)_2_}Ni(C_2_H_2_)]^−^ (4-Pt-)	1.304	1594	−412
[{H_2_C(3,5-(CH_3_)_2_Pz)_2_}Ni(C_2_H_2_)] (11-Ni)	1.291	1638	−369
[{H_2_C(3,5-(CH_3_)_2_Pz)_2_}Pd(C_2_H_2_)] (11-Pd)	1.285	1656	−350
[{H_2_C(3,5-(CH_3_)_2_Pz)_2_}Pt(C_2_H_2_)] (11-Pt)	1.305	1592	−415
[H_2_B(3,5-(CF_3_)_2_Pz)_2_]Li(C_2_H_2_) (4-Li)	1.210	1989	−18
[H_2_B(3,5-(CF_3_)_2_Pz)_2_]Na(C_2_H_2_) (4-Na)	1.209	1995	−12

a Δ*ν̄*_C≡C_ = *ν̄*_C≡C_ (metal complex) – *ν̄*_C≡C_ (free acetylene).

**Fig. 8:**
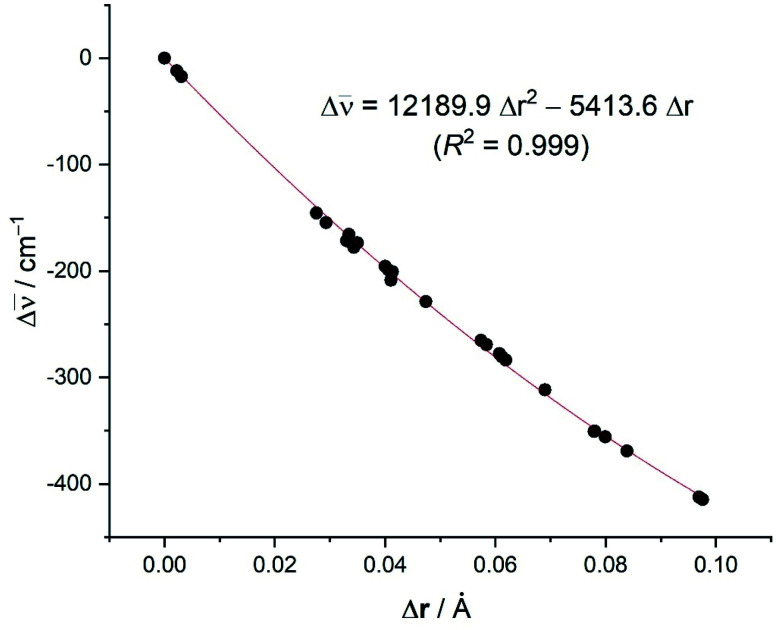
Plot of the computed difference in the C≡C bond distances *versus* the shift of the *ν̄*(C≡C) stretch (with respect to free acetylene: *r*_C≡C_ = 1.207 Å; *ν̄*_C≡C_ = 2007 cm^−1^).

From the data in Table 5, it becomes evident that, in all cases, the Cu(i)-complexes exhibit longer C≡C distances than their corresponding Ag(i)-analogues, which is translated into a higher redshift of the *ν̄*_C≡C_ stretching frequency. This is therefore fully consistent with the experimental findings and with the higher LM-(C_2_H_2_) interaction energies computed for the Cu(i)-complexes as compared to their Ag(i)-congeners (see above). This effect is even higher in the corresponding Au(i)-complexes which exhibit the longest C≡C distances in the entire group 11 series. Not surprisingly, even longer distances (associated with higher redshifts, *i.e.*, larger negative Δ*ν̄*_C≡C_ values) are found when considering the neutral group 10 transition metal as a consequence of a significantly stronger π-backdonation. In contrast, the analogous group 1 complexes, where the backbonding is minimal, present values rather similar to free acetylene. In addition, data presented in Table 5 show that fluorinated substituents on the supporting ligand L have a noticeable effect on *ν̄*_C≡C_ (see for example 4*vs.*4’; 4-Ag*vs.*4'-Ag). For this reason, it is not surprising that an excellent correlation was found when plotting the difference in the computed C≡C distances *vs.* the shift in the *ν̄*_C≡C_ stretching mode with respect to free acetylene (correlation coefficient of 0.999, see Fig. 8), including Δ*ν̄*_C≡C_ = 0 and Δ*r*_C≡C_ = 0 for free acetylene. The computed relationship presented in Fig. 8 can be then used to estimate the C≡C distances in the real systems (r(spec), where spec = spectroscopic, eqn (1)) and check the reliability of the X-ray derived data by simply adding the experimental C≡C distance in acetylene to the calculated distance change (Δ*r*(calc)) using the equation in Fig. 8 and the experimental Δ*ν̄*_C≡C_ value (Excel file to compute *r*(spec) from Δ*ν̄*_C≡C_ is provided in ESI†). A similar method has been utilized successfully by Krossing and co-workers^41^ to estimate the C=C bond distances (*i.e.*, to obtain spectroscopically assessed bond distances, *r*(spec)), of silver ethylene complexes.

1 *r*(spec) = 1.20286 + Δ*r*(calc)

As an example, the estimated C≡C bond distances (r(spec)) of 9 and 13 based on the experimental Raman data (Table 2, Δ*ν̄*_C≡C_ = −167 and −129 cm^−1^, respectively) are 1.236 and 1.228 Å, respectively. They are longer than that of the free acetylene, which is more reasonable and expected based on vibrational and computational data. The eqn (1) can also be used to estimate the C≡C bond distance of 8 (*i.e.*, using experimentally observed Δ*ν̄*_C≡C_ = −60 cm^−1^ to afford *r*(spec) = 1.214 Å), which is very close to the experimental X-ray model value of 1.209(1) Å based on high-resolution data.

**Fig. 9:**
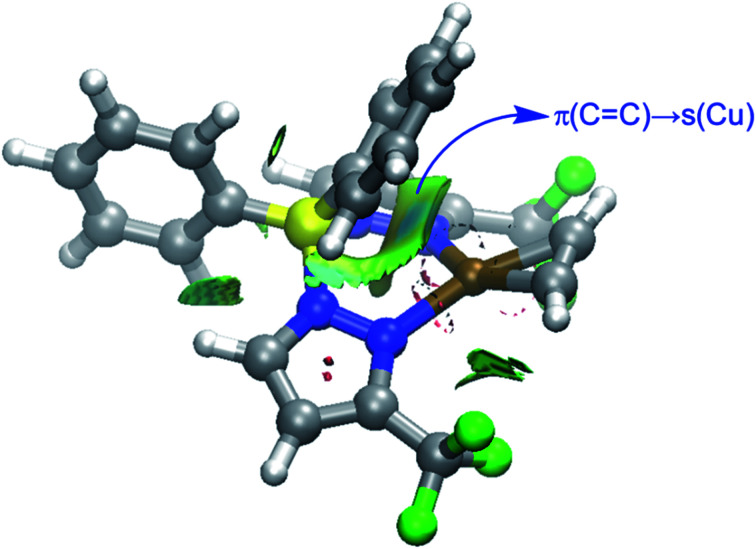
Contour plots of the reduced density gradient isosurfaces (density cutoff of 0.04 a.u.) for complex 9. The green surfaces indicate attractive noncovalent interactions.

Finally, we were curious to analyze the nature of the weak yet noticeable interaction between one of the phenyl groups attached to the boron atom and the transition metal in complexes 9 and 10 (see above). The NCIPLOT^45^ method clearly confirms the occurrence of a significant noncovalent attractive interaction (greenish surface in Fig. 9) between this aryl group and the transition metal. According to the Natural Orbital Bond (NBO)^46^ method, this stabilizing noncovalent interaction finds its origin in the donation of electron density from the closest π(C=C) molecular orbital of the phenyl group to the vacant s atomic orbital of the transition metal (associated stabilizing energy, Δ*E*(2) = −1.2 and −1.1 kcal mol^−1^, for complexes 9 and 10, respectively).

## Summary and conclusions

In summary, we have presented the isolation and complete characterization of several new acetylene adducts of Cu(i) and Ag(i) supported by [Ph_2_B(3-(CF_3_)Pz)_2_]^−^, [HB(3,5-(CF_3_)_2_Pz)_3_]^−^, [HB(3-(CF_3_),5-(Ph)Pz)_3_]^−^, and bis(pyrazolyl)methane H_2_C(3,5-(CH_3_)_2_Pz)_2_, as well as details on their ^1^H, ^13^C, and ^19^F NMR spectroscopy, Raman spectroscopy, and X-ray crystallography. According to our DFT calculations, the bonding situation in these complexes can be described in terms of the traditional Dewar–Chatt–Duncanson model involving two donor–acceptor interactions, namely σ-donation from the acetylene ligand to the transition metal and π-backdonation from the transition metal fragment to the π*(C≡C) molecular orbital of acetylene (the latter being markedly stronger than the former). Interestingly, the copper complexes exhibit a downfield shift for acetylenic carbons in their ^13^C NMR spectra and a more notable reduction in *ν̄*_C≡C_ relative to the free acetylene. This can be ascribed to a stronger interaction between the transition metal fragment and the acetylene ligand in the Cu(i)-complexes than that in their Ag(i)-counterparts as confirmed by our EDA-NOCV calculations (Δ*E*_int_ ∼ 20 kcal mol^−1^). Furthermore, it is found that while the replacement of bidentate fluorinated bis(pyrazolyl)borate ligand by the analogous non-fluorinated bis(pyrazolyl)methane ligand on M(I) ions provokes an almost negligible effect on the LM-(C_2_H_2_) interaction, the related tridentate tris(pyrazolyl)borate supporting ligand weakens the LM-(C_2_H_2_) interaction, which is reflected into less significant NMR/Raman shifts. The C≡C distance of these copper and silver acetylene complexes resulting from routine X-ray models suffers due to incomplete deconvolution of thermal smearing and anisotropy of the electron density in acetylene and is shorter than expected. Although it is possible to minimize this issue by collecting X-ray data at near absolute zero and to very high angles, it is not practical for routine work. However, the experimentally observed *ν̄*_C≡C_ values can be utilized to provide C≡C bond distances of Cu(i) and Ag(i) complexes that are more realistic. Molecules presented herein represent the largest collection of isolable copper(i) and silver(i) complexes featuring the terminal, η^2^-HC≡CH ligand. We believe that the contents of the present work contribute significantly to the development of acetylene chemistry.

## Data availability

The details and data supporting this article have been uploaded as the ESI.† Crystallographic data can be obtained from the CCDC.

## Author contributions

Project administration and conceptualization: HVRD; Writing – original draft and funding: HVRD and IF; Synthesis and spectroscopic characterizations: AN-P and SGR; X-ray crystallography: HVRD; Computational work: IF; Data analysis, review and editing: all authors.

## Conflicts of interest

There are no conflicts to declare.

## Supplementary Material

SC-013-D2SC02377F-s001

SC-013-D2SC02377F-s002

SC-013-D2SC02377F-s003
