# Supplementary material for: Isolable acetylene complexes of copper and silver
Source: Chem Sci. 2022 May 20;13(24):7190–203. doi: 10.1039/d2sc02377f (PMC9214850; doi:10.1039/d2sc02377f)
Supplement: SC-013-D2SC02377F-s001 [file SC-013-D2SC02377F-s001.pdf]

Electronic Supplementary Material (ESI) for Chemical Science.
This journal is © The Royal Society of Chemistry 2022

Isolable acetylene complexes of copper and silver

Anurag Noonikara-Poyil,[a] Shawn G. Ridlen,[a] Israel Fernández,[b,*] and H. V. Rasika Dias[a,*]

Affiliations:

[a] Department of Chemistry and Biochemistry, The University of Texas at Arlington, Arlington, Texas 76019, United States.

[b] Departamento de Química Orgánica I and Centro de Innovación en Química Avanzada (ORFEO-CINQA), Facultad de Ciencias Químicas, Universidad Complutense de Madrid, 28040-Madrid, Spain.

Table of Contents

General Information.......... S-2

Synthesis and characterization of metal complexes.......... S-3

^{1}H, ^{13}C, ^{19}F NMR, and Raman Spectra of Metal Complexes.......... S-8

Compound stability tests.......... S-24

X-ray Data Collection and Structure Determinations.......... S-25

Computational Details.......... S-56

References.......... S-73

General Information

All manipulations were carried out under an atmosphere of purified nitrogen using standard Schlenk techniques or in a MBraun glovebox equipped with a -25 °C refrigerator. Solvents were purchased from commercial sources and purified before use. NMR spectra were recorded at 25 °C on a JEOL Eclipse 500 spectrometer (^{1}H, 500.16 MHz ^{13}C, 125.78 MHz, and ^{19}F, 470.62 MHz) unless otherwise noted. ^{1}H and ^{13}C NMR spectra are referenced to the solvent peak (^{1}H; $CDCl_3$ δ 7.26, CD_2Cl_2 δ 5.32, $(CD_3)_2CO$ δ 2.05, ^{13}C; $CDCl_3$ δ 77.16, CD_2Cl_2 δ 53.84, $(CD_3)_2CO$ δ 29.84). ^{1}H NMR coupling constants (J) are reported in Hertz (Hz) and multiplicities are indicated as follows: s (singlet), d (doublet), t (triplet), q (quartet), m (multiplet). ^{19}F NMR values were referenced to external $CFCl_3$. Melting points were obtained on a Mel-Temp II apparatus and were not corrected. Elemental analyses were performed using a Perkin-Elmer Model 2400 CHN analyzer. IR spectra were collected at room temperature on a Shimadzu IR Prestige-21 FTIR containing an ATR attachment using pure liquid or solid materials, with instrument resolution at 2 cm^{-1}. Raman data were collected on a Thermo Scientific DXR3 Raman microscope with a HeNe laser source of 633 nm, by placing pure solid materials on a glass slide. $[Ph_2B(3\text{-}(CF_3)Pz)_2]Cu(C_2H_4)$,[1] $[HB(3,5\text{-}(CF_3)_2Pz)_3]Cu(C_2H_4)$,[2] $[\{H_2C(3,5\text{-}(CH_3)_2Pz)_2\}Cu(NCMe)][BF_4]$,[3] $[\{H_2C(3,5\text{-}(CH_3)_2Pz)_2\}Ag(C_2H_4)][SbF_6]$,[3] $[Ph_2B(3\text{-}(CF_3)_2Pz)_2]Tl$,[1] and $[HB(3\text{-}(CF_3),5\text{-}(Ph)Pz)_3]Na(THF)$[4] were synthesized according to the literature procedure. All other reactants and reagents were purchased from commercial sources. Heating was accomplished by either a heating mantle or a silicone oil bath.

***Warning**. Copper and silver in combination with acetylene gas can lead to potentially explosive materials and should be prepared in small quantities and handled with appropriate precautions. While no difficulties were encountered with the complexes reported herein, due caution should be exercised. Due care must also be taken when working with acetylene gas. It is known to produce explosive combinations with oxygen.*

Synthesis and Characterization of Metal complexes

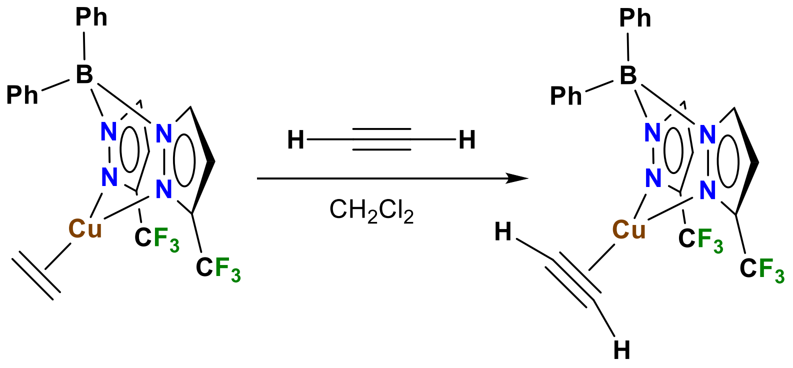

[$Ph_2B(3-(CF_3)Pz)_2$]Cu(C_2H_2) (9): [$Ph_2B(3-(CF_3)_2Pz)_2$]Cu(C_2H_4) (0.15 g, 0.28 mmol) was dissolved in 5 mL dichloromethane and stirred for ~3-5 min while bubbling acetylene. The reaction mixture was concentrated with continuous flow of acetylene and kept at -20 °C to obtain X-ray quality colorless crystals of [$Ph_2B(3-(CF_3)_2Pz)_2$]Cu(C_2H_2). Yield: 98%. M.P.: 118-120 °C (decomposition). Anal. Calc. $C_{22}H_{16}BCuF_6N_4$: C, 50.36; H, 3.07%; N, 10.68%. Found: C, 50.10%; H, 3.25%; N, 10.28%. ^{1}H NMR ($CDCl_3$, 500.16 MHz, 298 K): δ (ppm) 7.64 (s, br, 2H, Pz*H*), 7.25-7.23 (m, 6H, Ph*H*), 6.90 (br, 4H, Ph*H*), 6.56 (d, J = 2.0 Hz, 2H, Pz*H*), 4.22 (s, 2H, C_2H_2). ^{19}F NMR ($CDCl_3$, 470.62 MHz, 298 K): δ (ppm) -60.3 (s). $^{13}C\{^{1}H\}$ NMR ($CDCl_3$, 125.77 MHz, 298 K): δ (ppm) 146.3 (br), 142.8 (q, $^{2}J_{C\text{-}F}$ = 37.2 Hz, C-3), 138.3, 134.5 (br), 127.6, 127.4, 121.1 (q, $^{1}J_{C\text{-}F}$ =269.9 Hz, CF_3), 103.9 (C-4), 78.7 (C≡C). IR (cm^{-1}): 3202, 2932, 2212, 1807 (C≡C), 1523, 1496, 1433, 1369, 1275, 1259, 1211, 1084, 1078, 1012, 1003, 976. Raman (cm^{-1}): 3149, 3050, 1807(C≡C), 1593, 1522, 1372, 1176, 1143, 1032, 1000, 978.

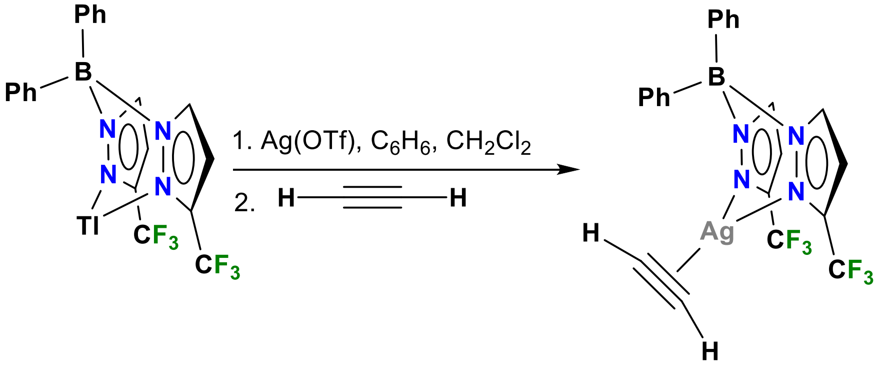

[$Ph_2B(3-(CF_3)Pz)_2$]Ag(C_2H_2) (10): [$Ph_2B(3-(CF_3)_2Pz)_2$]Tl (0.10 g, 0.16 mmol) and Ag(OTf) (0.044 g, 0.17 mmol) were placed in a Schlenk flask under nitrogen atmosphere. CH_2Cl_2 (10 mL) and benzene (1.0 mL) were combined in a separate Schlenk flask and slowly added via syringe. The resulting solution was stirred for 4 h at room temperature and filtered through a bed of

celite to remove a white precipitate. The resulting solution was concentrated to ~25% volume under reduced pressure and purified acetylene was gently bubbled through the solution for 2 minutes. The resulting mixture was placed in a freezer maintained at -20 °C to obtain colorless X-ray quality crystals of $[Ph_2B(3\text{-}(CF_3)_2Pz)_2]Ag(C_2H_2)$.Yield: 52%. M.P.: 109-112 °C (decomposition). ^{1}H NMR ($CDCl_3$, 500.16 MHz, 298 K): δ (ppm) 7.71 (s, br, 2H, Pz*H*), 7.25 (m, br, 6H, Ph*H*), 6.91 (br, 4H, Ph*H*), 6.50 (d, J = 2.3 Hz, 2H, Pz*H*), 2.13 (s, 2H, C_2H_2). ^{19}F NMR ($CDCl_3$, 470.62 MHz, 298 K): δ (ppm) -61.3 (s). $^{13}C\{^1H\}$ NMR ($CDCl_3$, 125.77 MHz, 298 K): δ (ppm) 147.3 (br), 142.7 (q, $^2J_{C\text{-}F}$ = 35.6 Hz, C-3), 138.7, 134.6 (br), 127.6, 127.0, 121.5 (q, $^1J_{C\text{-}F}$ =268.7 Hz, CF_3), 102.8 (C-4), 70.9 (C≡C). IR (cm^{-1}): 3009, 2928, 1531, 1524, 1495, 1433, 1424, 1370, 1274, 1268, 1258, 1194, 1184, 1166, 1159, 1077, 1013, 976, 955. Note: This compound loses acetylene in solution. The NMR data were collected in excess acetylene (some acetylene was added to the headspace of NMR tube) to minimize the formation of acetylene dissociated product. NMR spectra show two different species, desired product and acetylene dissociated molecule.

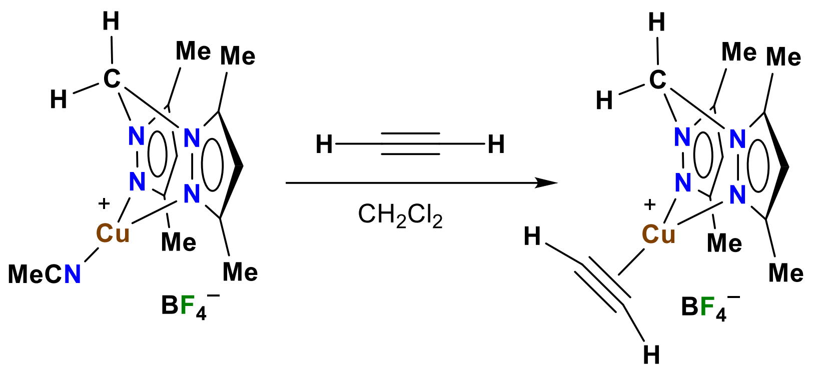

$[\{H_2C(3,5\text{-}(CH_3)_2Pz)_2\}Cu(C_2H_2)][BF_4]$ (11): $[\{H_2C(3,5\text{-}(CH_3)_2Pz)_2\}Cu(NCMe)][BF_4]$ (0.15 g, 0.38 mmol) was dissolved in 10 mL dichloromethane and stirred for ~3-5 min while bubbling acetylene. The reaction mixture was concentrated with continuous flow of acetylene and kept at -20 °C to obtain X-ray quality colorless crystals of $[\{H_2C(3,5\text{-}(CH_3)_2Pz)_2\}Cu(C_2H_2)][BF_4]$.Yield: 97%. M.P.: 113.-115 °C (decomposition). Anal. Calc. $C_{13}H_{18}BCuF_4N_4$: C, 41.02; H, 4.77%; N, 14.72%. Found: C, 40.61%; H, 4.45%; N, 14.12%. ^{1}H NMR ($(CD_3)_2CO$, 500.16 MHz, 298 K): δ (ppm) 6.56 (s, 2H, C*H*$_2$), 6.20 (s, 2H, Pz*H*), 5.14 (s, br, 2H, C_2H_2), 2.56 (s, 6H, C*H*$_3$), 2.33 (s, 6H, C*H*$_3$). $^{13}C\{^1H\}$ NMR ($(CD_3)_2CO$, 125.77 MHz, 298 K): δ (ppm) 153.0 (C-3/C-5), 143.9 (C-3/C-5), 108.0 (C-4), 79.5 (C≡C), 57.2 (*C*H$_2$), 13.8 (*C*H$_3$), 10.9 (*C*H$_3$). IR (cm^{-1}): 3233, 3257, 2927, 2854, 1812 (C≡C), 1557, 1468, 1441, 1437, 1420, 1393, 1385, 1284, 1275, 1148, 1051, 1038, 1003, 976, 961. Raman (cm^{-1}): 2976, 2957, 2933, 1812 (C≡C), 1762, 1699, 1658, 1461, 1389, 1275, 1254, 1052.

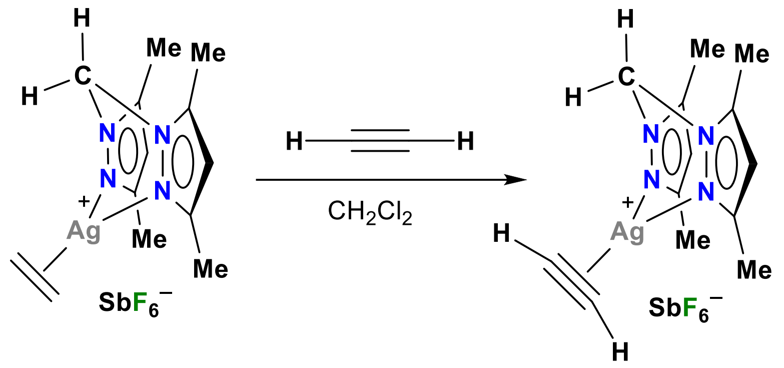

[{$H_2C(3,5-(CH_3)_2Pz)_2$}$Ag(C_2H_2)$][SbF_6] (12): [{$H_2C(3,5-(CH_3)_2Pz)_2$}$Ag(C_2H_4)$][SbF_6] (0.15 g, 0.26 mmol) was dissolved in 10 mL dichloromethane and stirred for ~2-3 min while bubbling acetylene. The reaction mixture was concentrated with continuous flow of acetylene and kept at -20 °C to obtain X-ray quality colorless crystals of [{$H_2C(3,5-(CH_3)_2Pz)_2$}$Ag(C_2H_2)$][SbF_6]. Yield: 93%. M.P.:119-122 °C (decomposition). Anal. Calc. $C_{13}H_{18}AgF_6N_4Sb$: C, 27.21; H, 3.16%; N, 9.76%. Found: C, 26.91%; H, 2.90%; N, 9.71%. ^{1}H NMR (CD_2Cl_2, 500.16 MHz, 298 K): δ (ppm) 6.35 (s, 2H, CH_2), 6.22 (s, 2H, Pz*H*), 2.39 (s, 6H, CH_3), 2.31 (s, 6H, CH_3), 2.25 (s, 2H, C_2H_2). $^{13}C\{^1H\}$ NMR (CD_2Cl_2, 125.77 MHz, 298 K): δ (ppm) 153.2 (C-3/C-5), 145.3 (C-3/C-5), 109.1 (C-4), 71.7 (C≡C), 61.2 (CH_2), 15.1 (CH_3), 11.8 (CH_3). IR (cm^{-1}): 3198, 2933, 2860, 2366, 1557, 1466, 1422, 1392, 1384, 1285, 1232, 1155, 1043, 981.

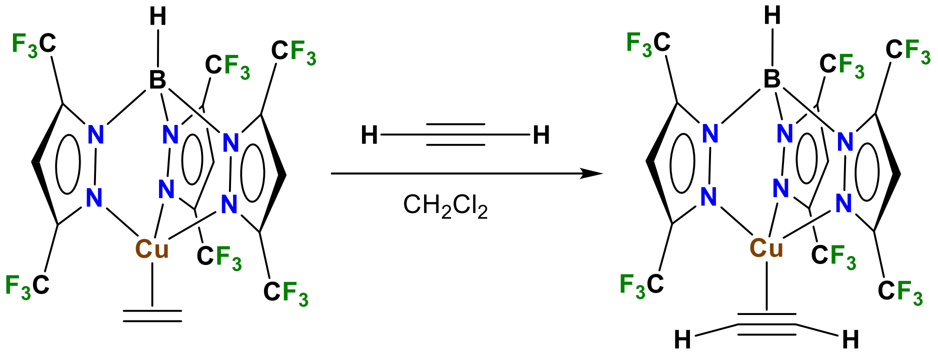

[$HB(3,5-(CF_3)_2Pz)_3$]$Cu(C_2H_2)$ (13): [$HB(3,5-(CF_3)_2Pz)_3$]$Cu(C_2H_4)$ (0.15 g, 0.21 mmol) was dissolved in 5 mL dichloromethane and stirred for ~3-5 min while bubbling acetylene. The remaining solvent was evaporated to obtain [$HB(3,5-(CF_3)_2Pz)_2$]$Cu(C_2H_2)$ as white powder. X-ray quality colorless crystals of [$HB(3,5-(CF_3)_2Pz)_3$]$Cu(C_2H_2)$ was obtained in acetylene saturated toluene at -20 °C. Yield: 98%. M.P.: 135-138 °C (decomposition). Anal. Calc. $C_{17}H_6BCuF_{18}N_6$: C, 28.73; H, 0.85%; N, 11.83%. Found: C, 29.15%; H, 1.10%; N, 12.20%. ^{1}H NMR ($CDCl_3$, 500.16 MHz, 298 K): δ (ppm) 6.94 (s, 3H, Pz*H*), 4.50 (s, 2H, C_2H_2). ^{19}F NMR ($CDCl_3$, 470.62 MHz, 298 K): δ (ppm) -59.2(s), -60.7(s). $^{13}C\{^1H\}$ NMR ($CDCl_3$, 125.77 MHz, 298 K): δ (ppm) 143.5 (q, $^2J_{C\text{-}F}$ = 39.6 Hz, C-3/C-5), 140.0 (q, $^2J_{C\text{-}F}$ = 38.4 Hz, C-3/C-5), 120.0 (q, $^1J_{C\text{-}F}$ =269.9 Hz, CF_3),

119.1 (q, $^1J_{C\text{-}F}$ =271.1 Hz, CF_3), 107.1 (C-4), 75.8 (C≡C). IR (cm^{-1}): 3235, 2932, 2623 (B-H), 1849 (C≡C), 1558, 1497, 1396, 1368, 1265, 1247, 1178, 1134, 1098, 1080, 1042, 999, 989. Raman (cm^{-1}):3321, 3224, 3169, 2704, 1845 (C≡C), 1762, 1699, 1658, 1500, 1472, 1386, 1259, 993.

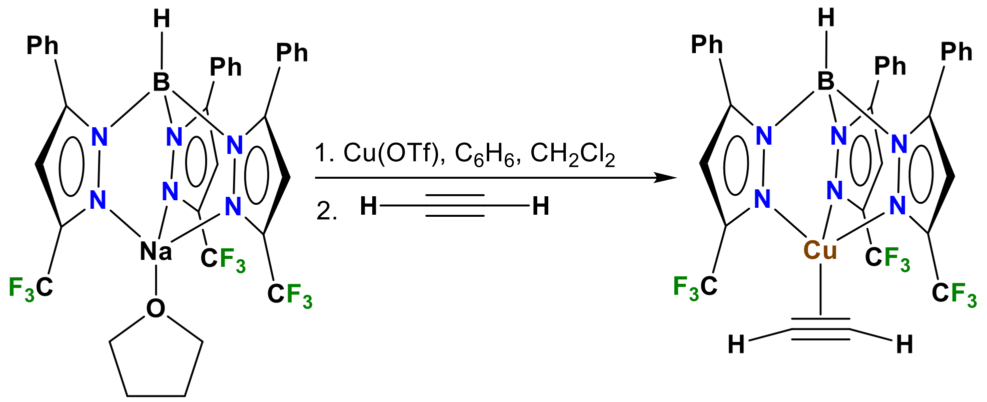

[HB(3-(CF_3),5-(Ph)Pz)$_3$]Cu(C_2H_2) (14): [HB(3-(CF_3),5-(Ph)Pz)$_3$]Na(THF) (0.200 g, 0.270 mmol) and [CuOTf]$_2$·C_6H_6 (0.076 g, 0.151 mmol) were placed in a Schlenk flask under nitrogen atmosphere. CH_2Cl_2 (20 mL) and benzene (2.0 mL) were combined in a separate Schlenk flask and slowly added via syringe. The resulting solution was stirred overnight at room temperature and filtered through a bed of Celite to remove a white precipitate. The resulting solution was concentrated to ~25% volume under reduced pressure and purified acetylene was gently bubbled through the solution for 2 minutes. The resulting mixture was placed in a freezer maintained at -20 °C to obtain colorless X-ray quality crystals. Yield: 0.136 g, 69%. M.P.: 103-154 °C (slowly decomposes over this wide temperature range with a final melting point of the residue at 225 °C). Analysis Calcd. for $C_{32}H_{21}CuBF_9N_6$: C, 52.30; H, 2.88; N, 11.44%. Found. C, 52.43; H, 2.76; N, 11.59%. ^{1}H NMR ($CDCl_3$, 500.16 MHz, 298 K): δ (ppm) 7.27 (t, 3H, Ph-*H*, J = 7.5 Hz), 6.94 (t, 6H, Ph-*H*, J =7.7 Hz), 6.87 (d, 6H, Ph-*H*, J = 7.2 Hz), 6.56 (s, 3H, Pz*H*), 4.66 (s, 2H, C_2H_2). ^{19}F NMR (CD_2Cl_2, 470.62 MHz, 298 K): δ (ppm) -60.0 (s). ^{13}C{^{1}H} NMR ($CDCl_3$, 125.77 MHz, 298 K) δ (ppm): 150.4 (s, *C*(C_6H_5)), 142.7 (q, $^2J_{C\text{-}F}$ = 38.4 Hz, *C*CF_3), 130.6 (Ph), 129.8 (Ph), 128.5 (Ph), 128.0 (Ph), 121.2 (q, $^1J_{C\text{-}F}$ = 272.3 Hz, *C*F_3), 105.3 (C-4), 76.5 (*C*$_2H_2$). ^{1}H NMR (CD_2Cl_2, 500.16 MHZ, 298 K): δ (ppm) 7.28 (t, 3H, Ph*H*, J = 7.5 Hz), 6.96 (t, 6H, Ph*H*, J = 7.7 Hz), 6.89 (d, 6H, Ph*H*, J = 7.5 Hz), 6.60 (s, 3H, C*H*), 4.72 (s, 2H, C_2H_2). ^{19}F NMR (CD_2Cl_2, 470.62 MHz, 298 K): δ (ppm) -60.3 (s). ^{13}C{^{1}H} NMR (CD_2Cl_2, 125.77 MHz, 298 K): δ (ppm) 151.0 (*C*(C_6H_5)), 142.7 (q, $^2J_{C\text{-}F}$ = 38.4 Hz, *C*CF_3), 130.7 (Ph), 130.0 (Ph), 128.9 (Ph), 128.3 (Ph), 121.6 (q, $^1J_{C\text{-}F}$ = 272.3 Hz, *C*F_3), 105.5 (C-4), 76.8 (*C*$_2H_2$). IR (Selected peaks, cm^{-1}): 2644 (B-H). Raman (Selected peaks, cm^{-1}): 1829 (C≡C).

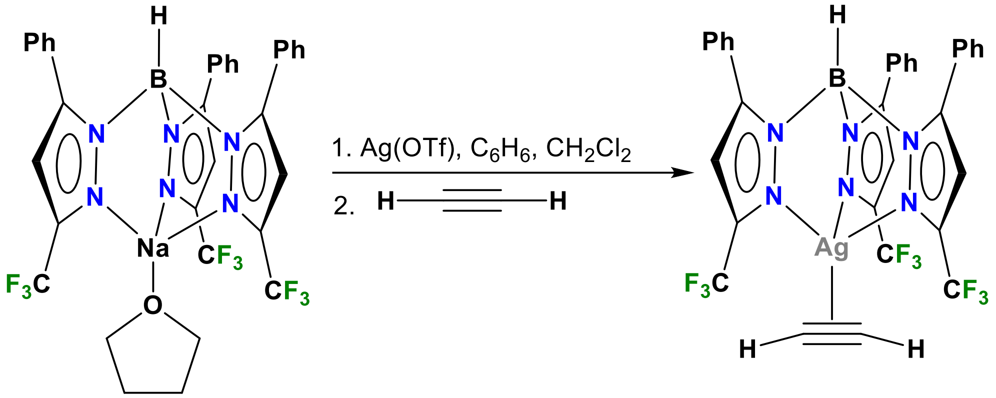

[HB(3-(CF₃),5-(Ph)Pz)₃]Ag(C₂H₂) (15): [HB(3-(CF₃),5-(Ph)Pz)₃]Na(THF) (0.25 g, 0.34 mmol) and AgOTf (0.095 g, 0.37 mmol) were placed in a Schlenk flask under nitrogen atmosphere. CH_2Cl_2 (20 mL) and benzene (2.0 mL) were combined in a separate Schlenk flask and slowly added via syringe. The resulting solution was stirred overnight at room temperature and filtered through a bed of Celite to remove an orange precipitate. The resulting solution was concentrated to ~25% volume under reduced pressure and purified acetylene was gently bubbled through the solution for 2 minutes. The resulting mixture was placed in a freezer maintained at -20 °C to obtain colorless X-ray quality crystals. Yield: 0.186 g, 71%. M.P.: 103-148 °C (slowly decomposes over this wide temperature range with a final melting point of the residue at 225 °C). Analysis Calcd. for $C_{32}H_{21}AgBF_9N_6$: C, 49.32; H, 2.72; N, 10.79%. Found. C, 49.70; H, 2.64; N, 10.53%. ^{1}H NMR (CD_2Cl_2, 500.16 MHz, 298 K): δ (ppm) 7.23 (t, 3H, Ph*H*, J = 7.5 Hz), 6.94 (t, 6H, Ph*H*, J = 7.5 Hz), 6.89 (d, 6H, Ph*H*, J = 6.9 Hz), 6.56 (s, 3H, C*H*), 4.72 (br, 1H, B*H*), 3.59 (s, 2H, C_2H_2). ^{13}C{^{1}H} NMR (CD_2Cl_2, 125.77 MHz, 298 K): δ (ppm) 151.9 (s, *C*(C_6H_5)), 142.9 (q, $^2J_{C\text{-}F}$ = 37.2 Hz, *C*CF_3), 131.3 (Ph), 130.0 (Ph), 128.5 (Ph), 128.2 (Ph), 122.0 (q, $^1J_{C\text{-}F}$ = 268.7 Hz, *C*F_3), 104.9 (*C*H), 66.7 (s, C_2H_2). ^{19}F NMR (CD_2Cl_2, 470.62 MHz, 298 K): δ (ppm) -61.3 (s). IR (Selected peaks, cm^{-1}): 2629 (B-H). Raman (Selected peaks, cm^{-1}): 1895 (C≡C).

^{1}H, ^{13}C, ^{19}F NMR, and Raman Spectra of Metal Complexes

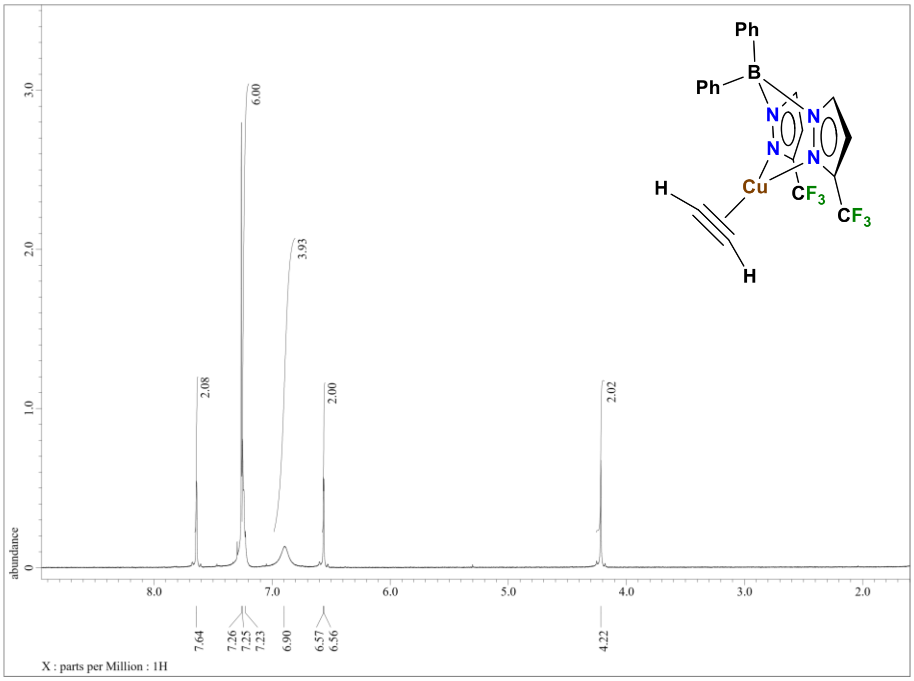

Figure S1: ^{1}H NMR Spectrum of $[Ph_2B(3\text{-}(CF_3)Pz)_2]Cu(C_2H_2)$ **(9)** in $CDCl_3$.

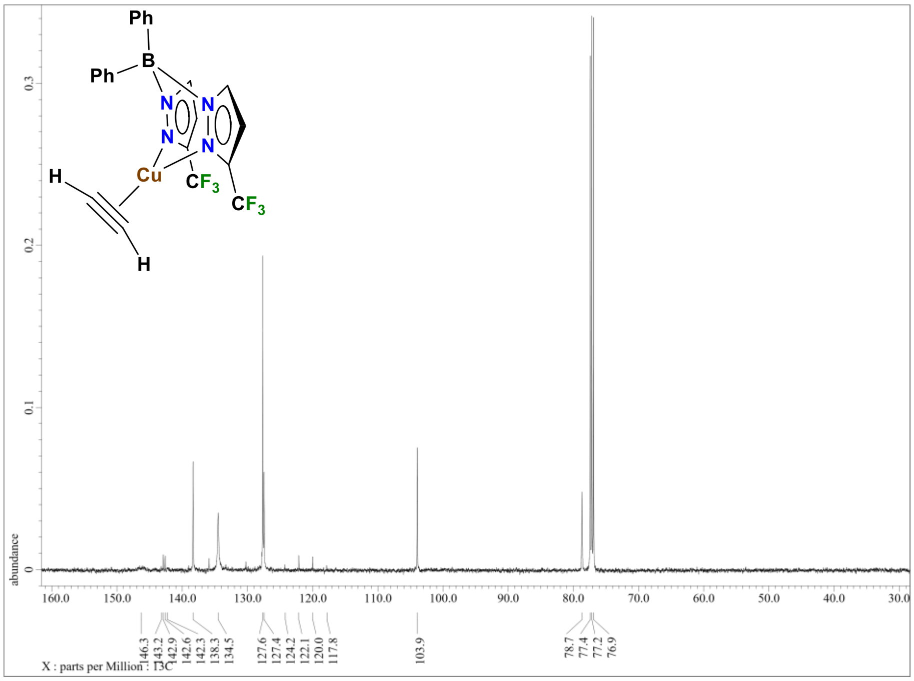

Figure S2: ^{13}C NMR Spectrum of $[Ph_2B(3\text{-}(CF_3)Pz)_2]Cu(C_2H_2)$ **(9)** in $CDCl_3$.

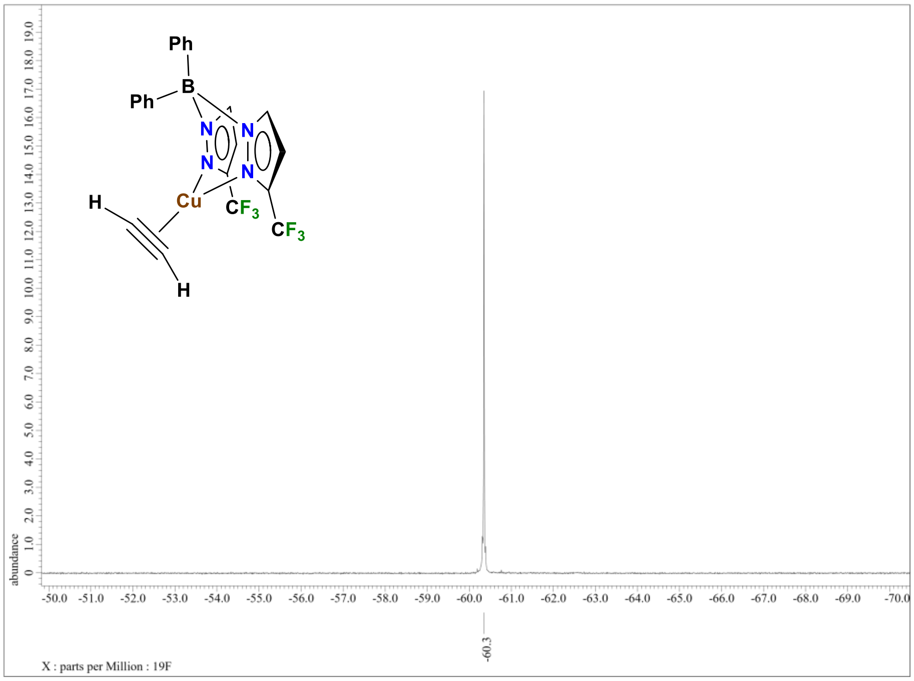

Figure S3: ^{19}F NMR Spectrum of $[Ph_2B(3\text{-}(CF_3)Pz)_2]Cu(C_2H_2)$ **(9)** in $CDCl_3$.

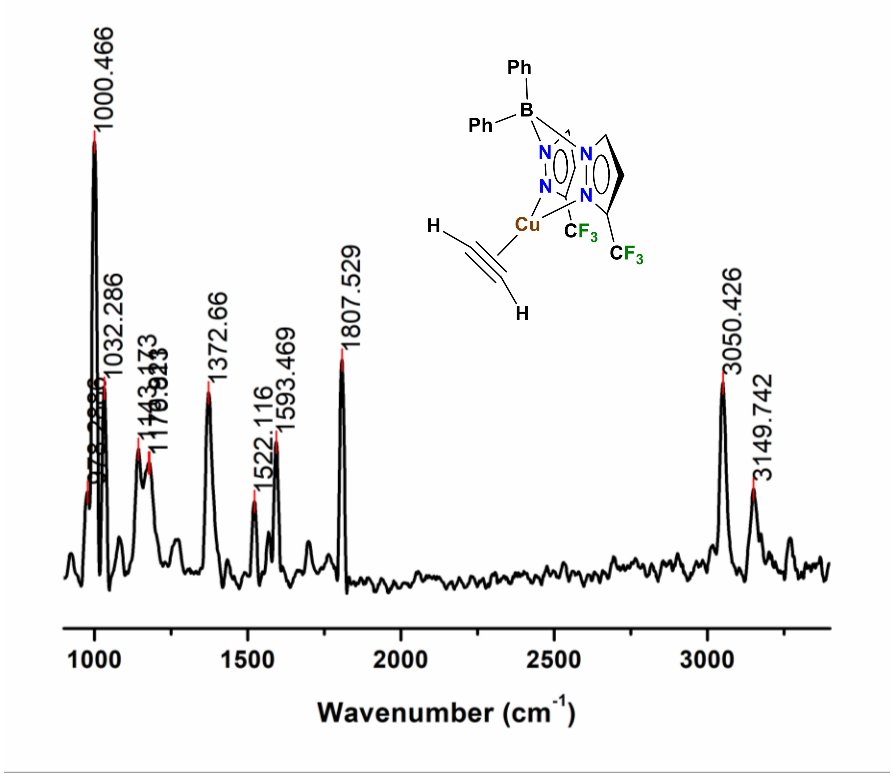

Figure S4: Raman Spectrum of $[Ph_2B(3\text{-}(CF_3)Pz)_2]Cu(C_2H_2)$ **(9)**.

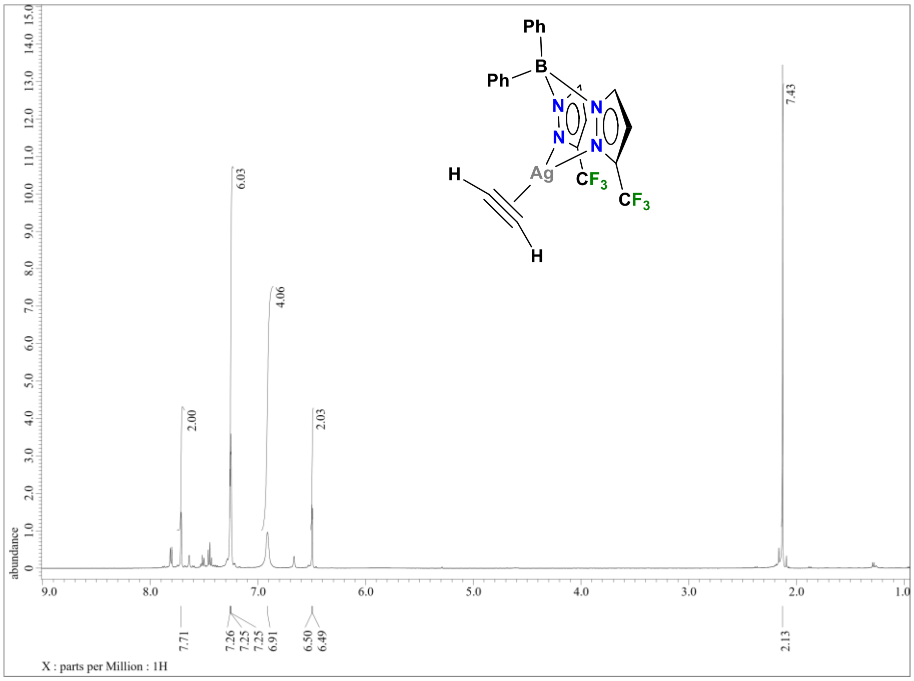

Figure S5: ^{1}H NMR Spectrum of $[Ph_2B(3\text{-}(CF_3)Pz)_2]Ag(C_2H_2)$ **(10)** in $CDCl_3$. The NMR data were collected in the presence of excess acetylene (some C_2H_2 was added to the headspace of the NMR tube).

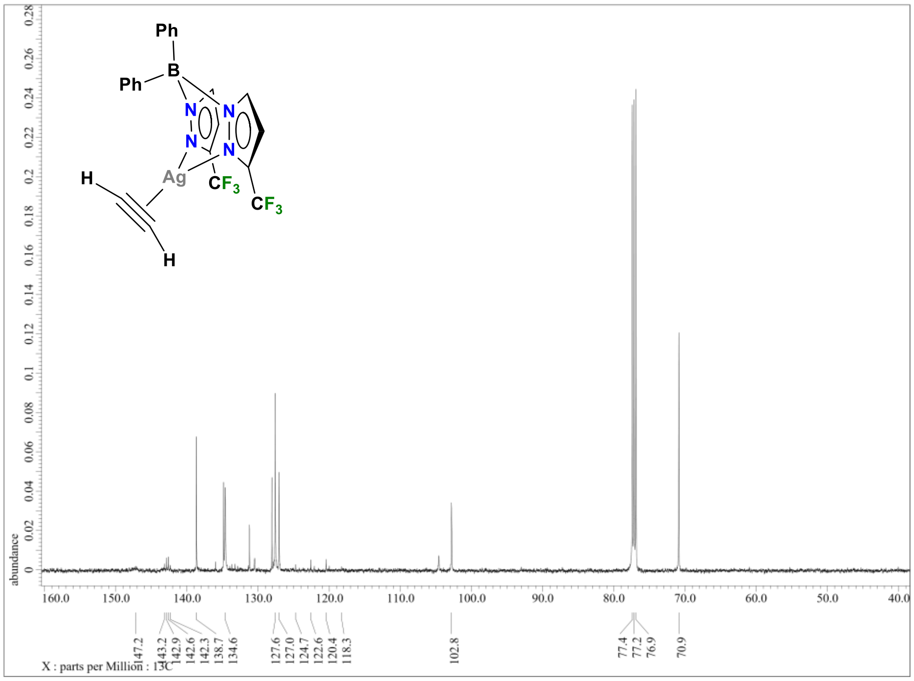

Figure S6: ^{13}C NMR Spectrum of $[Ph_2B(3\text{-}(CF_3)Pz)_2]Ag(C_2H_2)$ **(10)** in $CDCl_3$. The NMR data were collected in the presence of excess acetylene (some C_2H_2 was added to the headspace of the NMR tube).

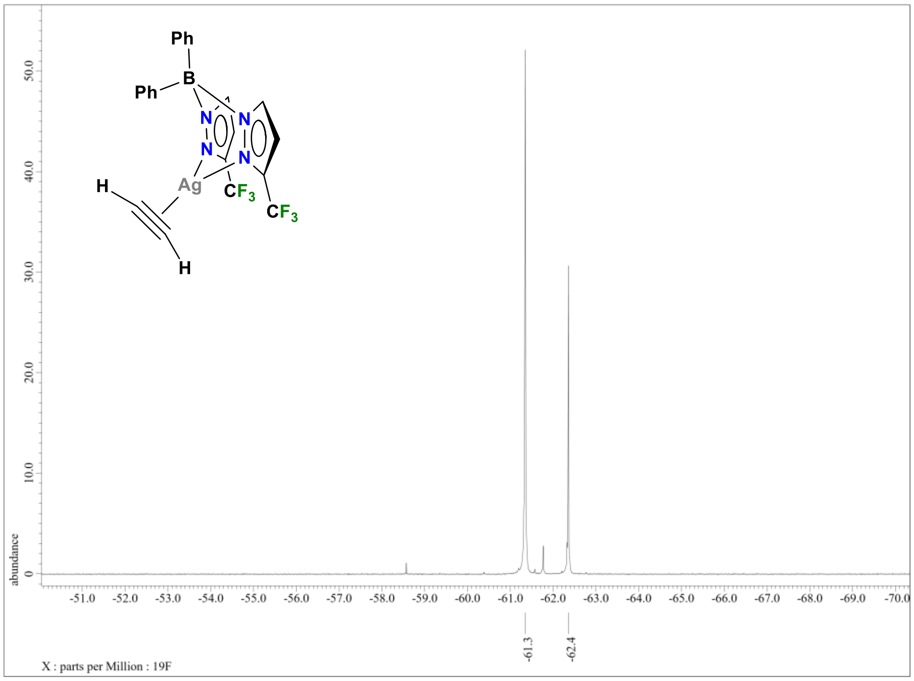

Figure S7: ^{19}F NMR Spectrum of $[Ph_2B(3\text{-}(CF_3)Pz)_2]Ag(C_2H_2)$ **(10)** in $CDCl_3$. The peak at -62.4 ppm belongs to the acetylene dissociated product. The NMR data were collected in the presence of excess acetylene (some C_2H_2 was added to the headspace of the NMR tube).

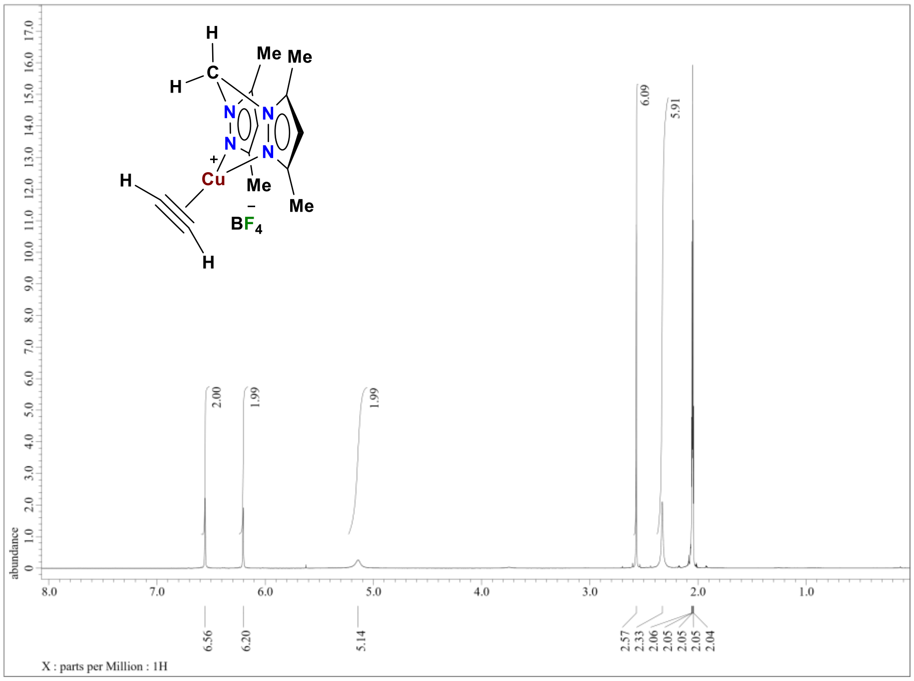

Figure S8: ^{1}H NMR Spectrum of $[\{H_2C(3,5\text{-}(CH_3)_2Pz)_2\}Cu(C_2H_2)][BF_4]$ **(11)** in $(CD_3)_2CO$.

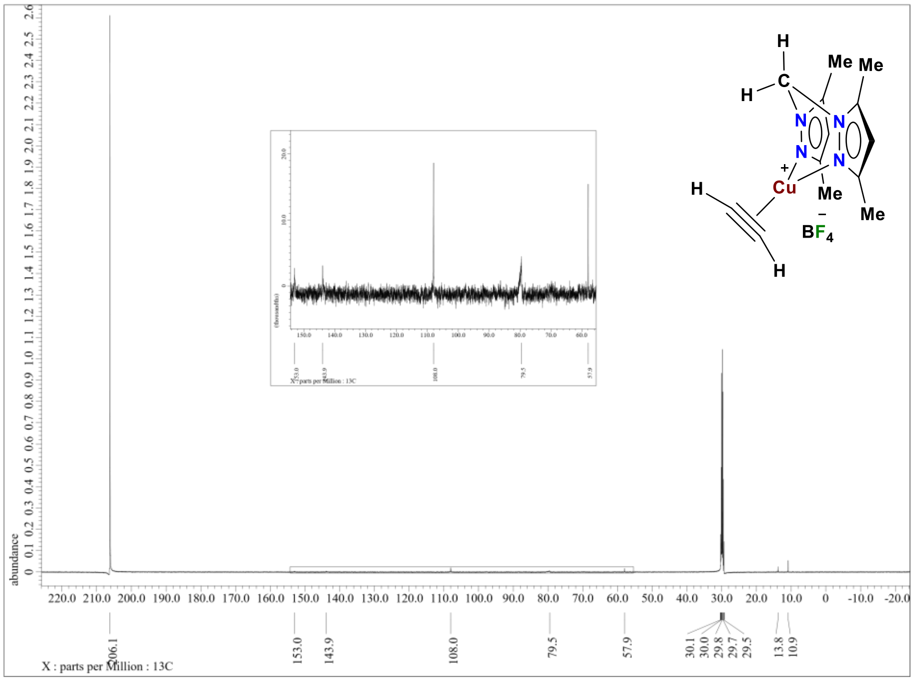

Figure S9: ^{13}C NMR Spectrum of [{$H_2C(3,5\text{-}(CH_3)_2Pz)_2$}$Cu(C_2H_2)$][$BF_4$] **(11)** in $(CD_3)_2CO$.

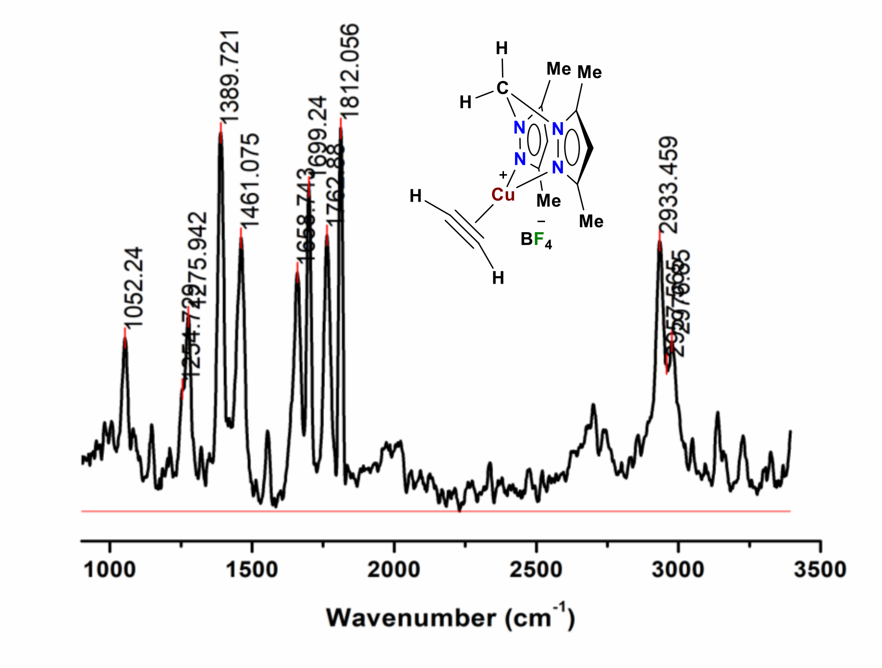

Figure S10: Raman Spectrum of [{$H_2C(3,5\text{-}(CH_3)_2Pz)_2$}$Cu(C_2H_2)$][$BF_4$] **(11)**.

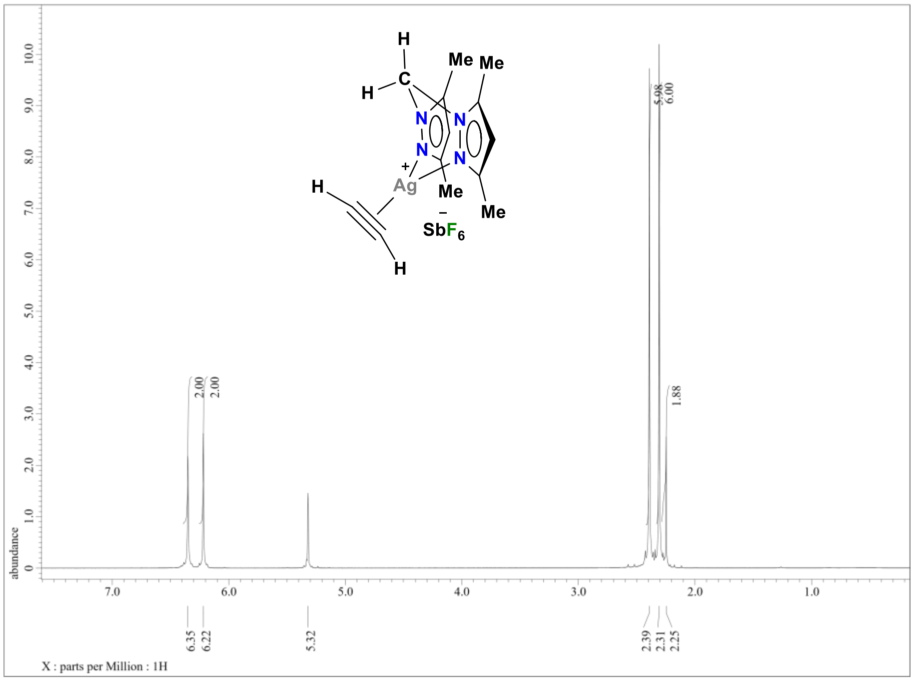

Figure S11: ^{1}H NMR Spectrum of [{$H_2C(3,5-(CH_3)_2Pz)_2$}$Ag(C_2H_2)$][SbF_6] **(12)** in CD_2Cl_2.

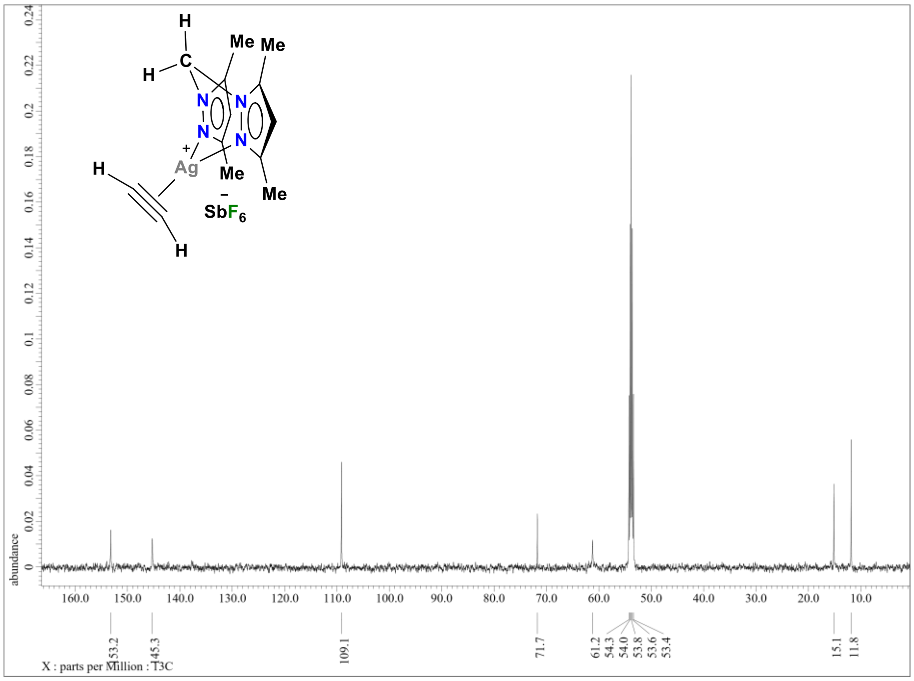

Figure S12: ^{13}C NMR Spectrum of [{$H_2C(3,5-(CH_3)_2Pz)_2$}$Ag(C_2H_2)$][SbF_6] **(12)** in CD_2Cl_2.

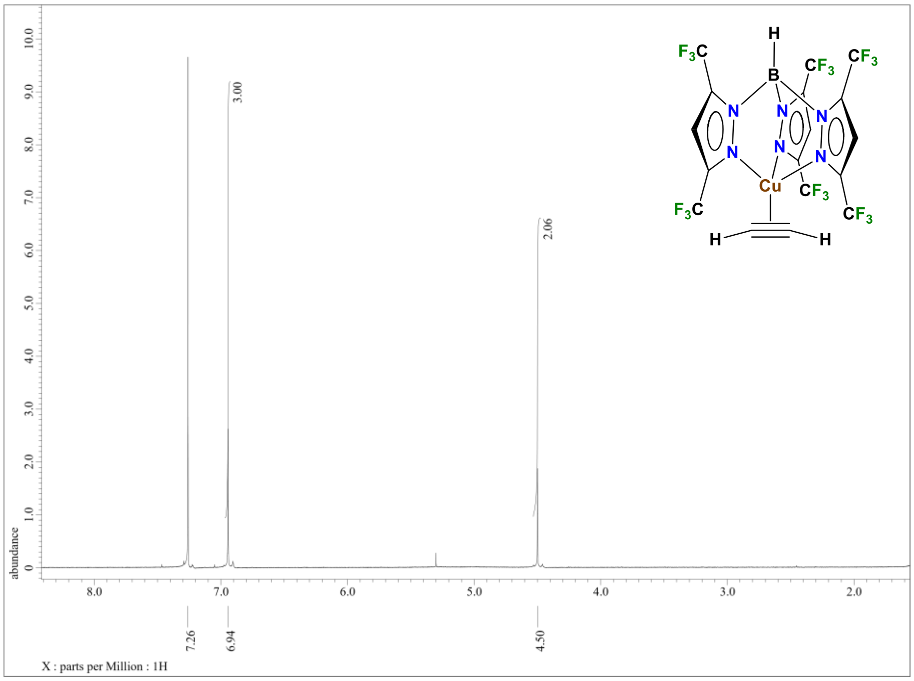

Figure S13: ^{1}H NMR Spectrum of [HB(3,5-$(CF_3)_2$Pz$)_3$]Cu(C_2H_2) **(13)** in $CDCl_3$.

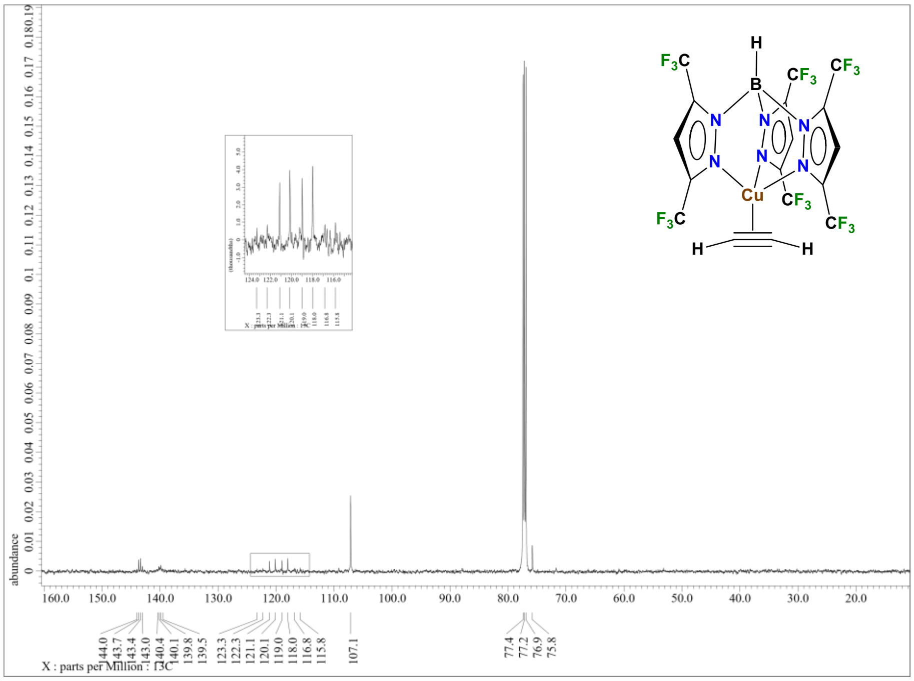

Figure S14: ^{13}C NMR Spectrum of [HB(3,5-$(CF_3)_2$Pz$)_3$]Cu(C_2H_2) **(13)** in $CDCl_3$.

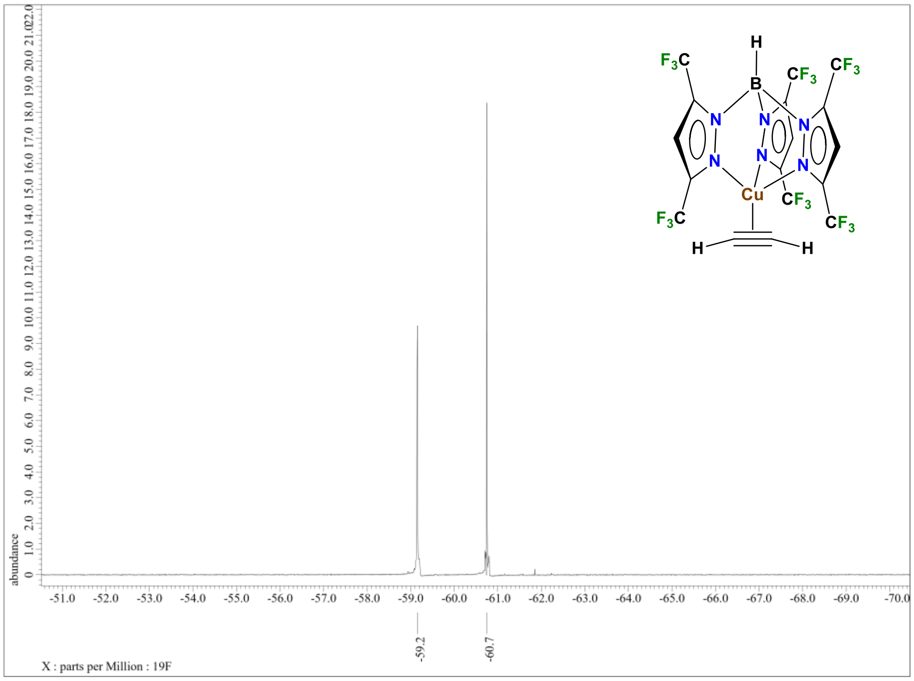

Figure S15: ^{19}F NMR Spectrum of $[HB(3,5\text{-}(CF_3)_2Pz)_3]Cu(C_2H_2)$ **(13)** in $CDCl_3$.

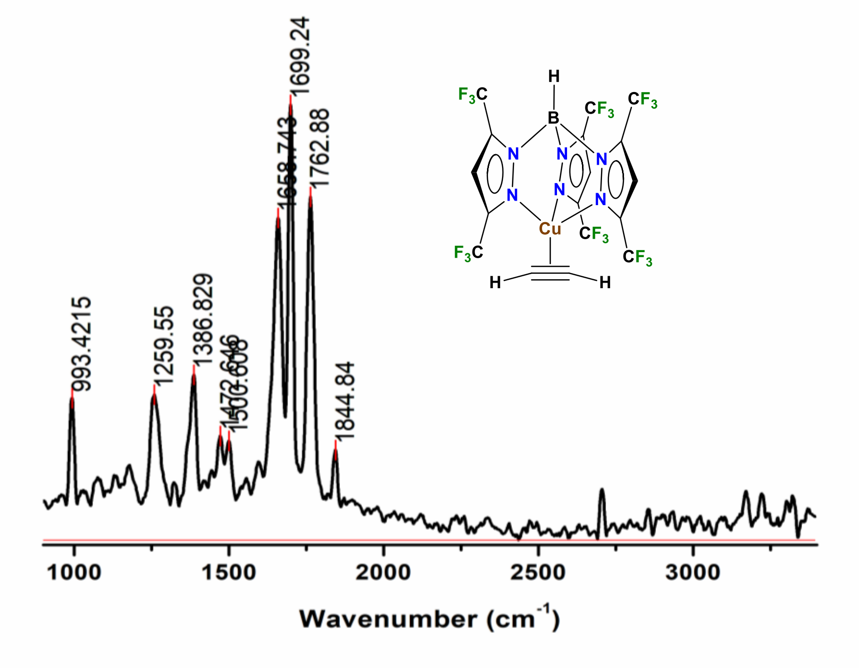

Figure S16: Raman Spectrum of $[HB(3,5\text{-}(CF_3)_2Pz)_3]Cu(C_2H_2)$ **(13)**.

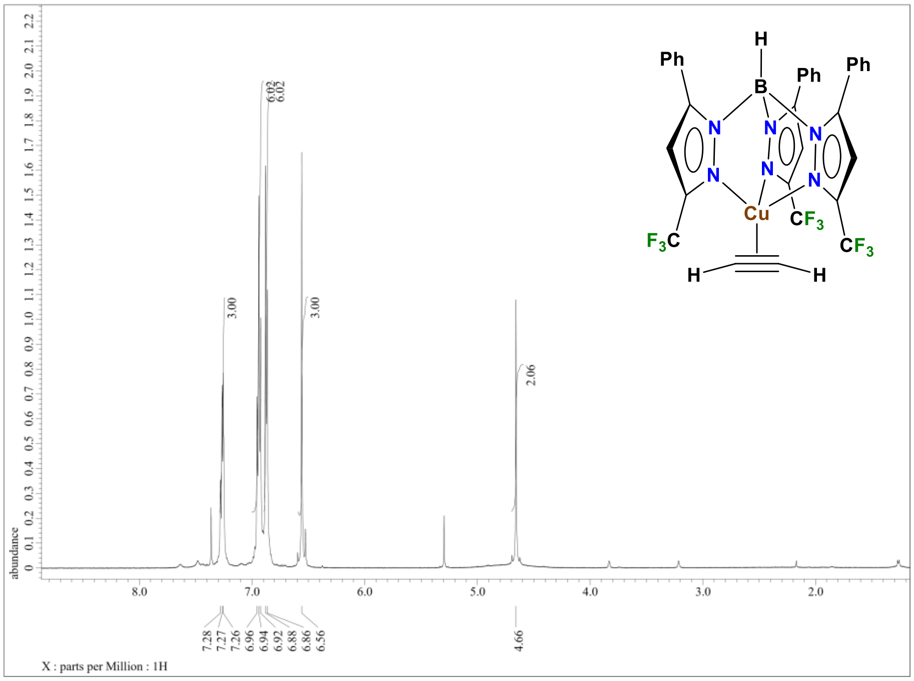

Figure S17: ^{1}H NMR Spectrum of [HB(3-(CF_3),5-(Ph)Pz)$_3$]Cu(C_2H_2) **(14)** in $CDCl_3$.

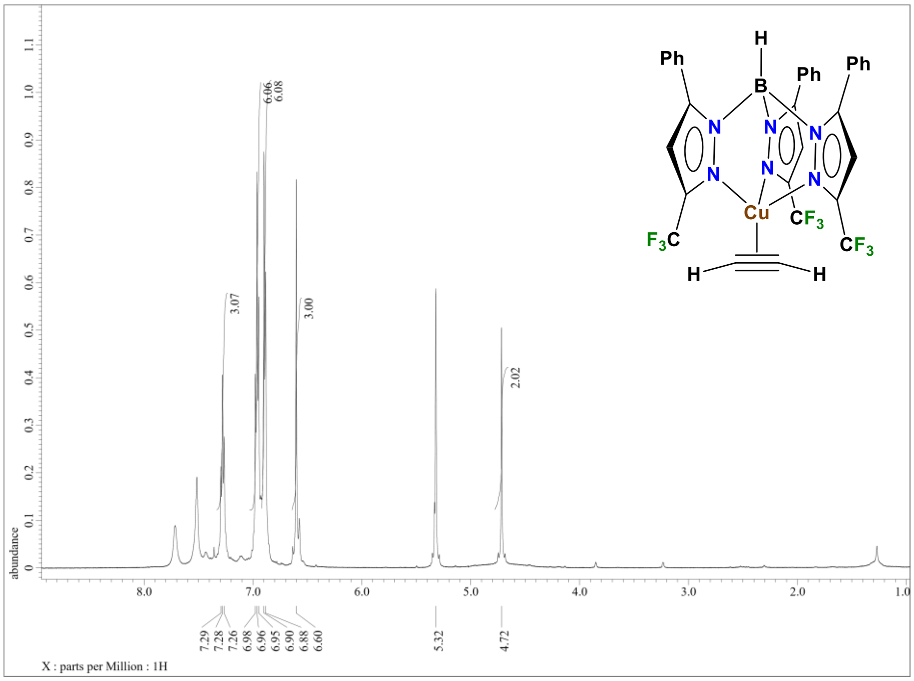

Figure S18: ^{1}H NMR Spectrum of [HB(3-(CF_3),5-(Ph)Pz)$_3$]Cu(C_2H_2) **(14)** in CD_2Cl_2.

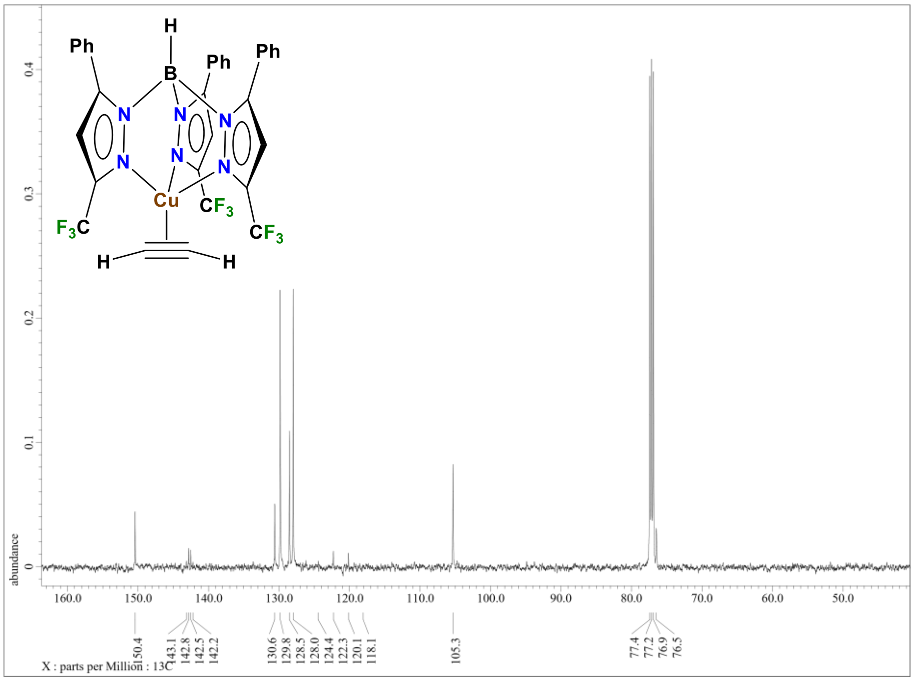

Figure S19: ^{13}C NMR Spectrum of [HB(3-(CF_3),5-(Ph)Pz)$_3$]Cu(C_2H_2) **(14)** in $CDCl_3$.

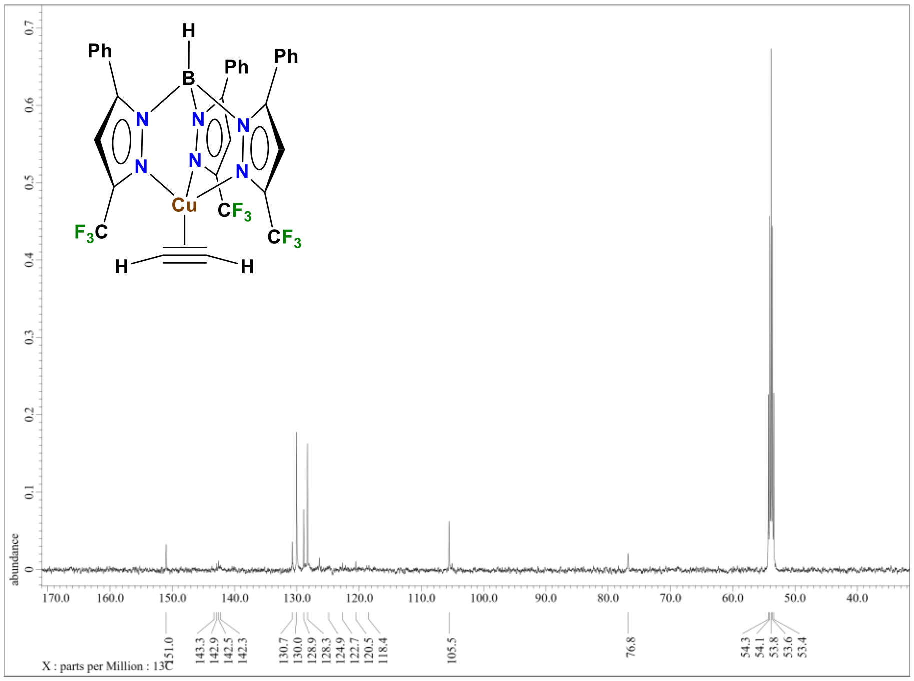

Figure S20: ^{13}C NMR Spectrum of [HB(3-(CF_3),5-(Ph)Pz)$_3$]Cu(C_2H_2) **(14)** in CD_2Cl_2.

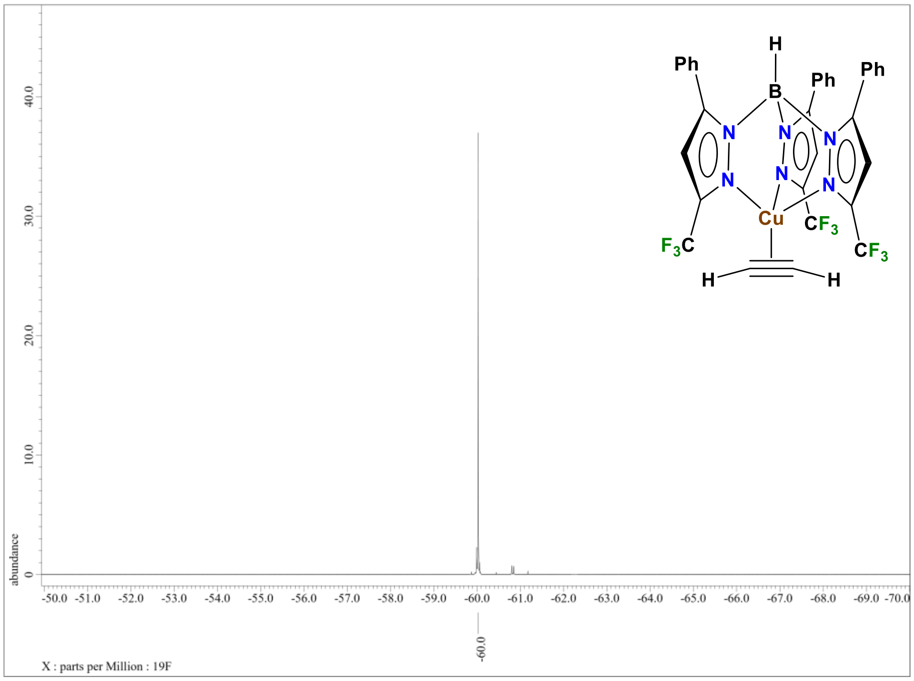

Figure S21: ^{19}F NMR Spectrum of [HB(3-(CF_3),5-(Ph)Pz)$_3$]Cu(C_2H_2) **(14)** in $CDCl_3$

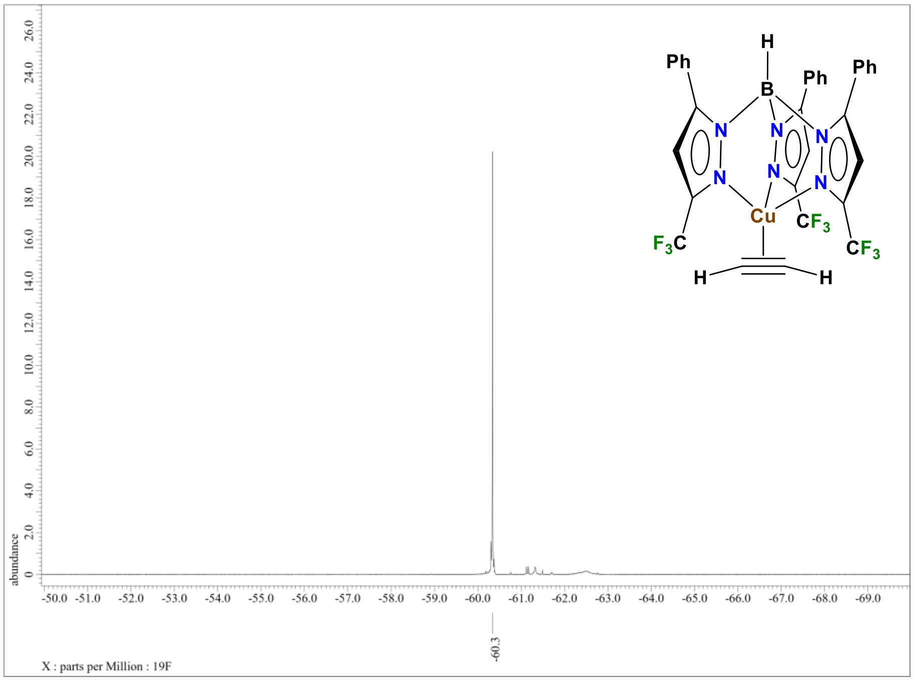

Figure S22: ^{19}F NMR Spectrum of [HB(3-(CF_3),5-(Ph)Pz)$_3$]Cu(C_2H_2) **(14)** in CD_2Cl_2.

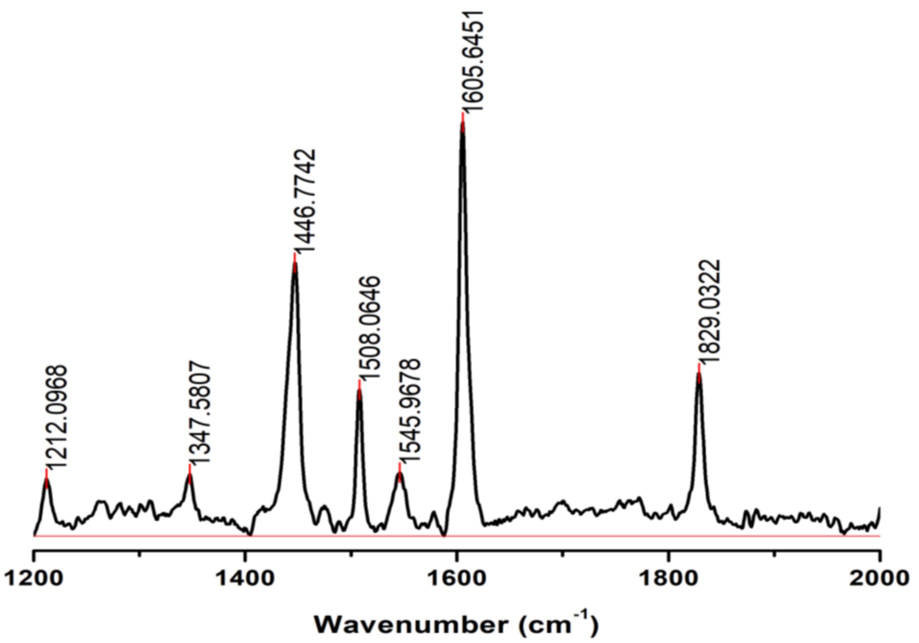

Figure S23: Raman Spectrum of [HB(3-(CF_3),5-(Ph)Pz)$_3$]Cu(C_2H_2) **(14)**.

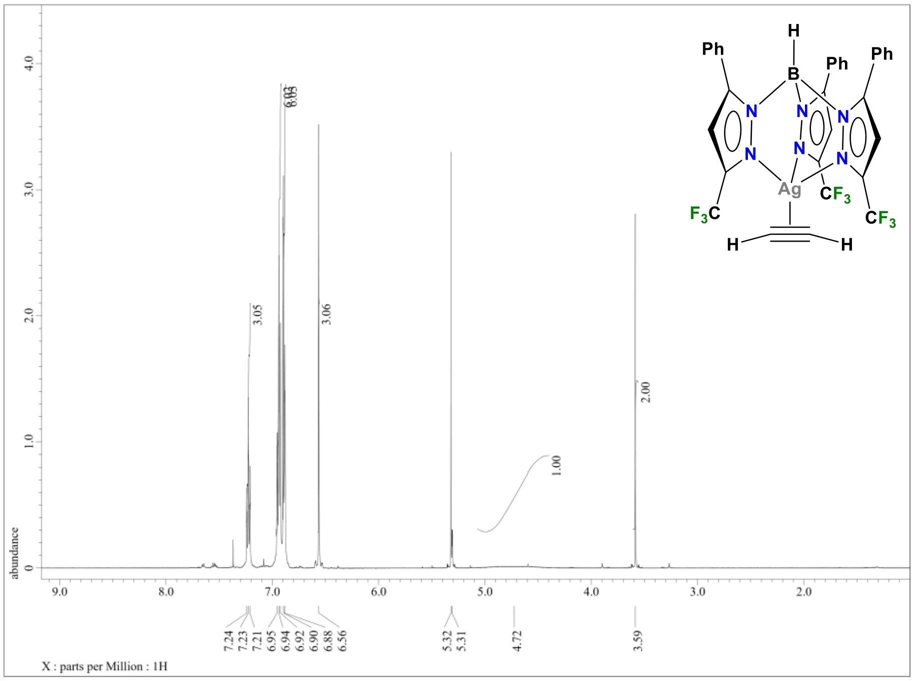

Figure S24: ^{1}H NMR Spectrum of [HB(3-(CF_3),5-(Ph)Pz)$_3$]Ag(C_2H_2) **(15)** in CD_2Cl_2.

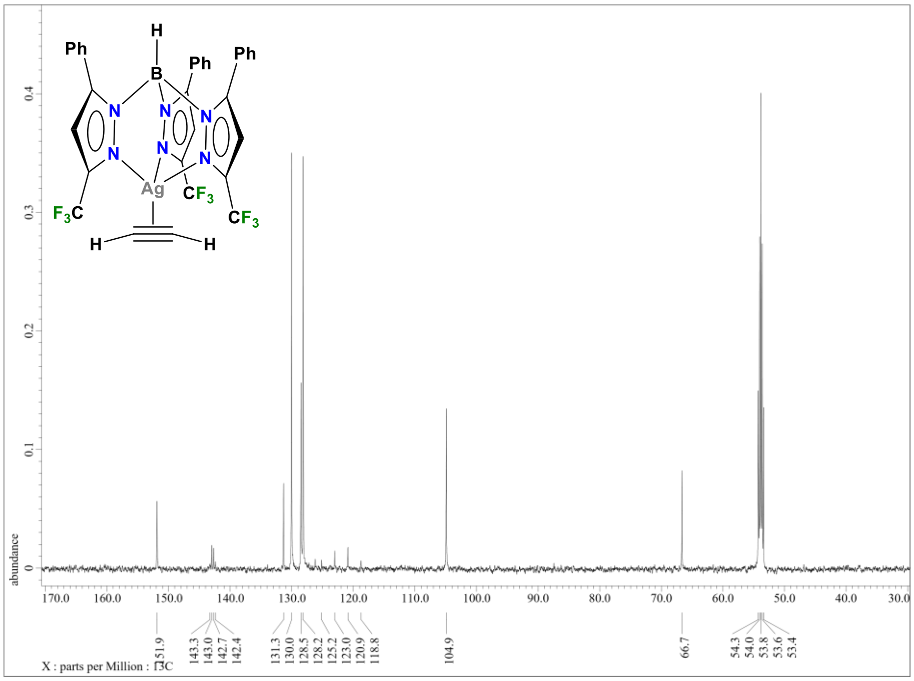

Figure S25: ^{13}C NMR Spectrum of $[HB(3\text{-}(CF_3),5\text{-}(Ph)Pz)_3]Ag(C_2H_2)$ **(15)** in CD_2Cl_2.

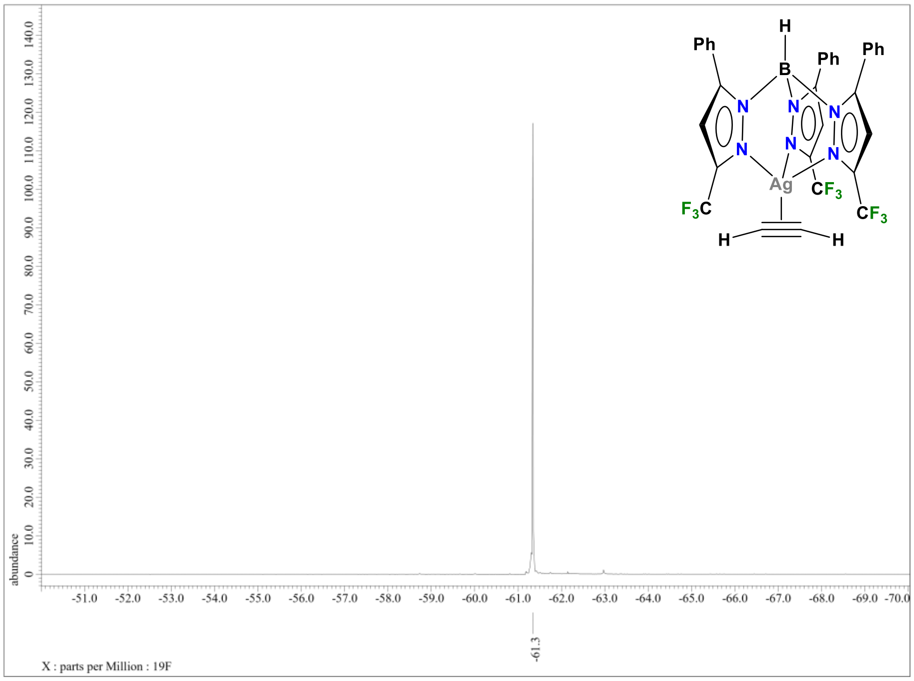

Figure S26: ^{19}F NMR Spectrum of $[HB(3\text{-}(CF_3),5\text{-}(Ph)Pz)_3]Ag(C_2H_2)$ **(15)** in CD_2Cl_2

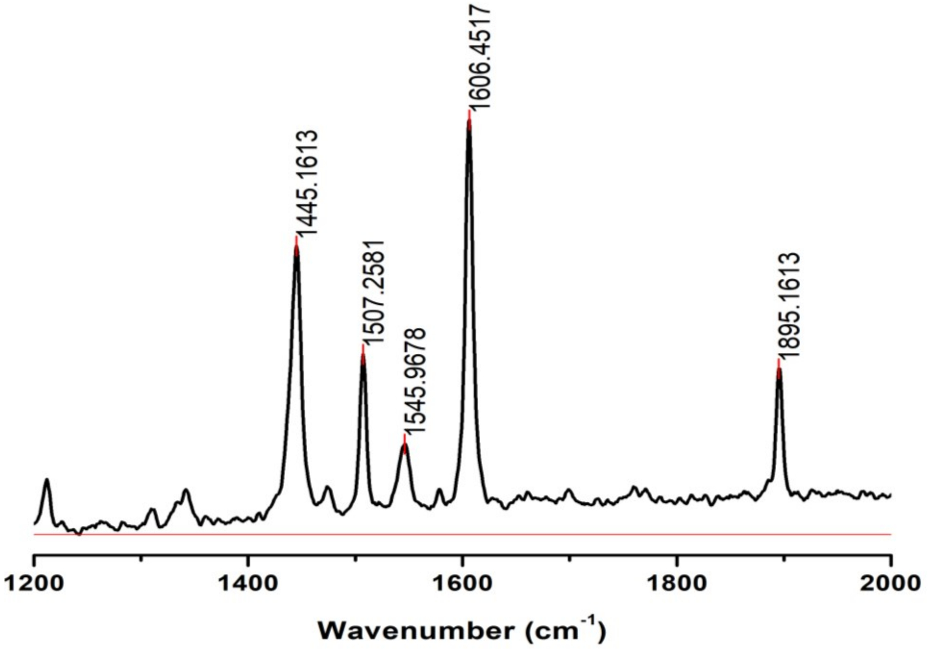

Figure S27: Raman Spectrum of [HB(3-(CF_3),5-(Ph)Pz$)_3$]Ag(C_2H_2) **(15)**.

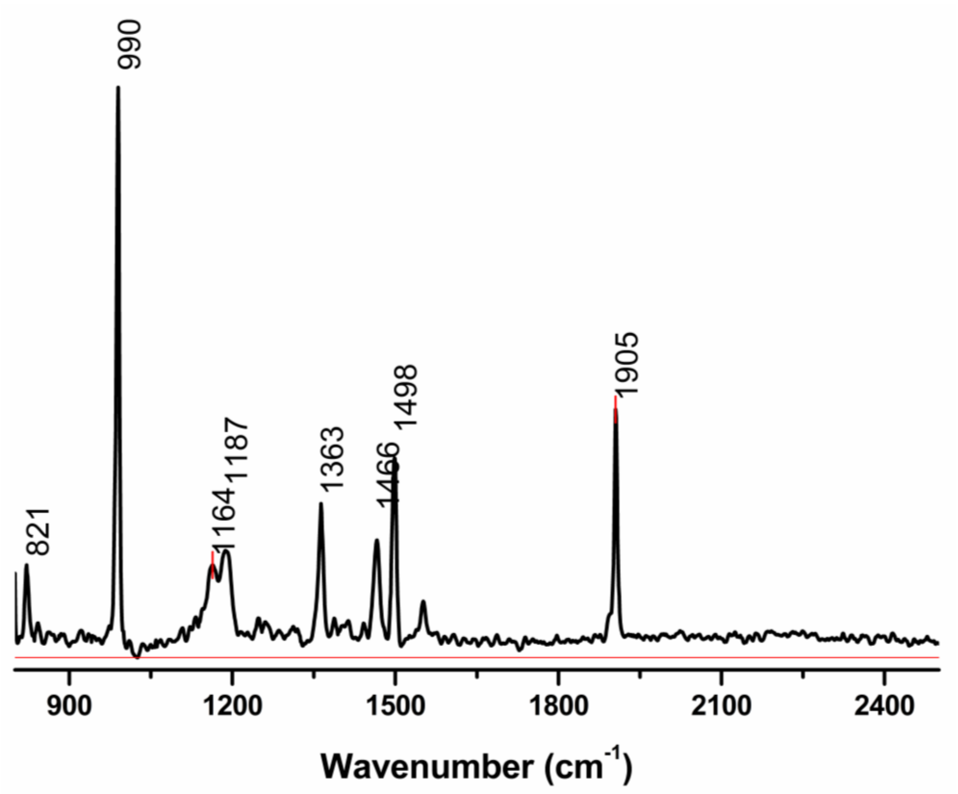

Figure S28: Raman Spectrum of [HB(3,5-(CF_3)$_2$Pz)$_3$]Ag(C_2H_2) **(5)**. This complex was synthesized as previously reported.[5]

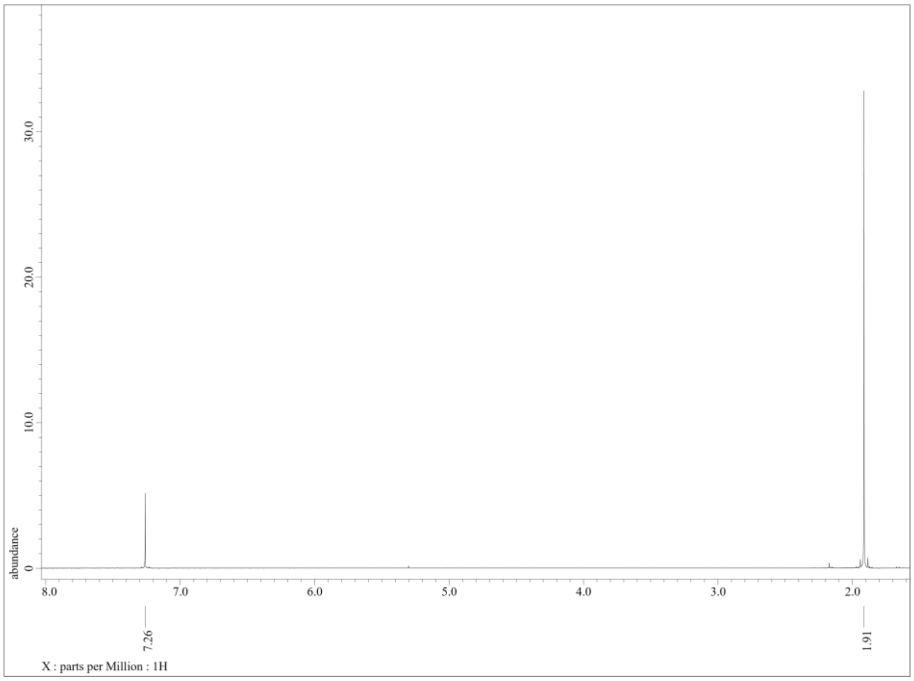

Figure S29: ^{1}H NMR Spectrum of free acetylene in $CDCl_3$.

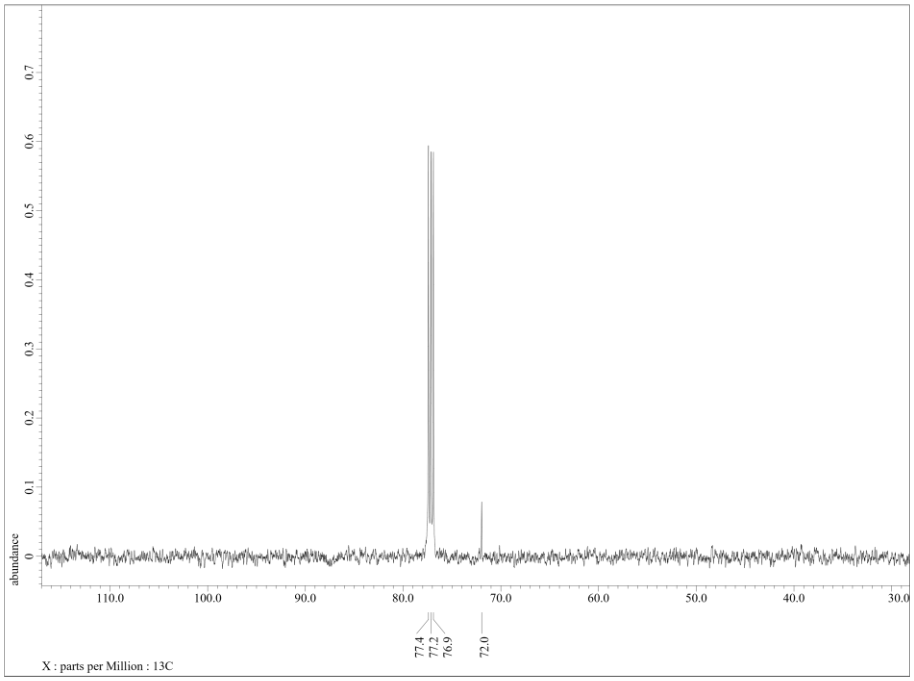

Figure S30: ^{13}C NMR Spectrum of free acetylene in $CDCl_3$.

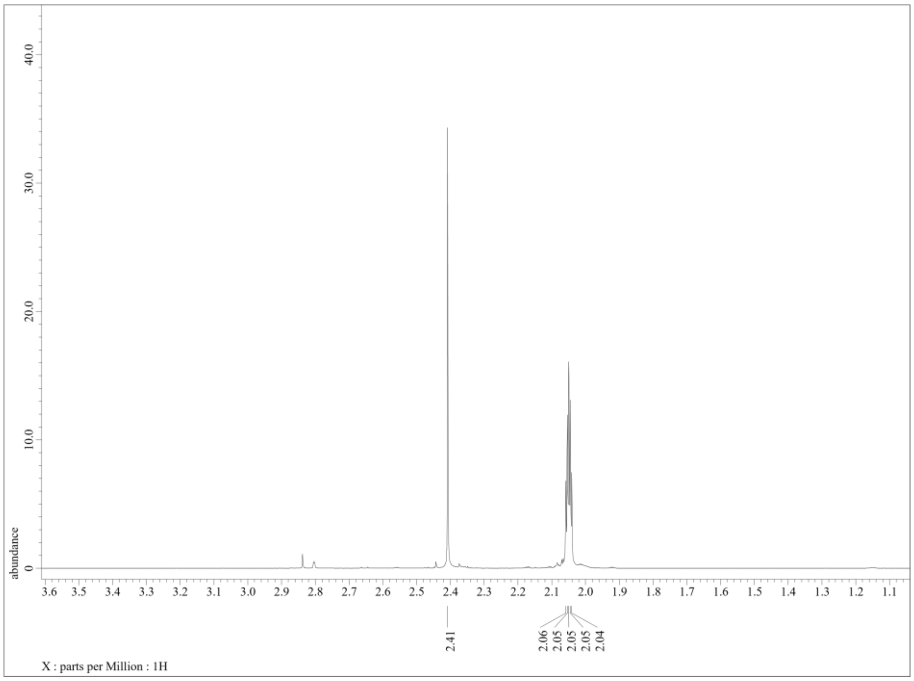

Figure S31: ^{1}H NMR Spectrum of free acetylene in $(CD_3)_2CO$.

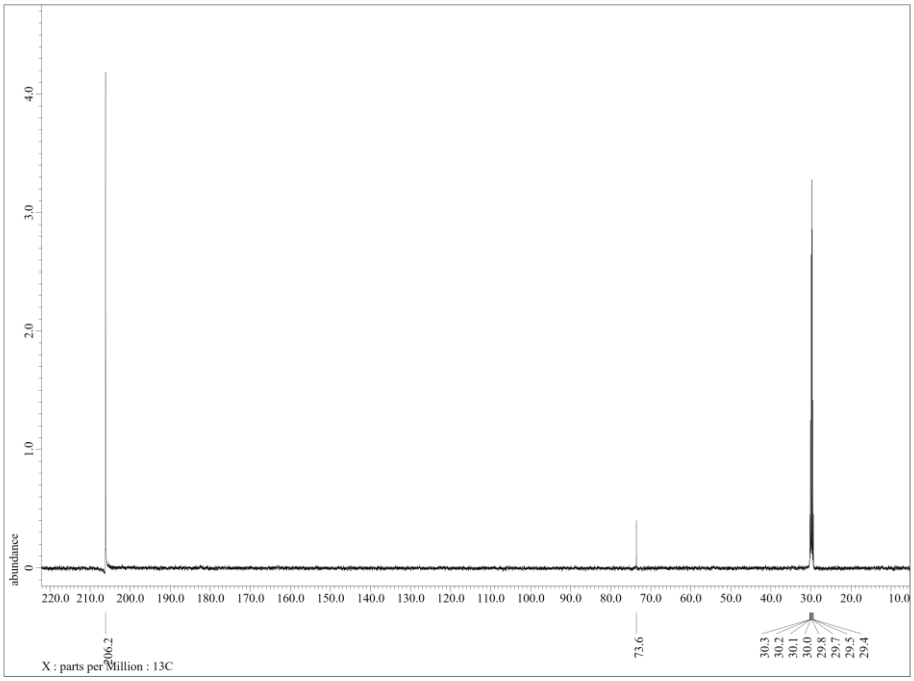

Figure S32: ^{13}C NMR Spectrum of free acetylene in $(CD_3)_2CO$.

Compound stability tests

<u>Solids</u>: About 5 mg of each complex was placed in a vial and kept either open to air or under N_2 atmosphere or acetylene gas at room temperature for 16 h. The stability of solids under reduced pressure was tested by placing samples under vacuum (~5 torr) for 1 h at room temperature. After the specified time, samples were dissolved in $CDCl_3$ and analyzed by ^{1}H NMR. These spectra were compared to the spectra obtained for each sample at 0 h in the same solvent. Note that solid samples can be handled in air for short periods to prepare NMR samples without any signs of decomposition.

<u>Solutions</u>: About 5 mg of each complex was dissolved in $CDCl_3$ (~0.6 mL) and ^{1}H NMR data were collected immediately afterward. None of the samples (except **10**, which shows some C_2H_2 dissociation) show signs of decomposition or acetylene loss until the first data collection point (about 15 mins to collect ^{1}H NMR data). These NMR tubes were stored either open to air or under N_2 at room temperature for 16 h and tested again using ^{1}H NMR spectrum to record the observations. Some copper samples turned to blue/green while certain silver adducts showed darkening (black deposits) as indicated below as color change but their NMR data did not indicate signs of significant ligand decomposition.

Table S1. Stability of copper and silver acetylene complexes under different conditions at ambient temperature.

**Metal complex**	**Solid**				**Solution ($CDCl_3$)**		
	Under C_2H_2	Under N_2 after 16 h	Open air after 16 h	Vacuum after 1 h	After 15 min	Under N_2 after 16 h	Open air after 16 h
$[HB(3,5-(CF_3)_2Pz)_3]Cu(C_2H_2)$ (**13**)	stable	stable	stable	stable	stable	stable	stable
$[HB(3,5-(CF_3)_2Pz)_3]Ag(C_2H_2)$ (**5**)	stable	stable	stable	stable	stable	stable	stable
$[HB(3-(CF_3),5-(Ph)Pz)_3]Cu(C_2H_2)$ (**14**)	stable	stable	stable	stable	stable	stable	stable
$[HB(3-(CF_3),5-(Ph)Pz)_3]Ag(C_2H_2)$ (**15**)	stable	stable	complete C_2H_2 dissociation	stable	stable	stable / color change	complete C_2H_2 dissociation / color change
$[Ph_2B(3-(CF_3)Pz)_2]Cu(C_2H_2)$ (**9**)	stable	stable	partial C_2H_2 dissociation / color change	stable	stable	stable	partial C_2H_2 dissociation / color change
$[Ph_2B(3-(CF_3)Pz)_2]Ag(C_2H_2)$ (**10**)	stable	partial C_2H_2 dissociation	complete C_2H_2 dissociation	complete C_2H_2 dissociation	partial C_2H_2 dissociation	partial C_2H_2 dissociation	complete C_2H_2 dissociation
$[\{H_2C(3,5-(CH_3)_2Pz)_2\}Cu(C_2H_2)][BF_4]$ (**11**)	stable	partial C_2H_2 dissociation	complete C_2H_2 dissociation / color change	complete C_2H_2 dissociation	stable	partial C_2H_2 dissociation	complete C_2H_2 dissociation / color change
$[\{H_2C(3,5-(CH_3)_2Pz)_2\}Ag(C_2H_2)][SbF_6]$ (**12**)	stable	partial C_2H_2 dissociation	complete C_2H_2 dissociation	complete C_2H_2 dissociation	stable	complete C_2H_2 dissociation	complete C_2H_2 dissociation

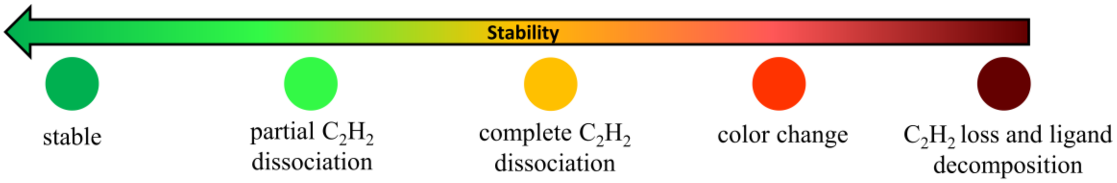

X-ray Data Collection and Structure Determinations

A suitable crystal covered with a layer of hydrocarbon/Paratone-N oil was selected and mounted on a Cryo-loop, and immediately placed in the low temperature nitrogen stream. The X-ray intensity data were measured at 100(2) K on a Bruker D8 Quest with a Photon 100 ($[Ph_2B(3-(CF_3)Pz)_2]Ag(C_2H_2)$, $[\{H_2C(3,5-(Me)_2Pz)_2\}Cu(C_2H_2)][BF_4]$, $[\{H_2C(3,5-(Me)_2Pz)_2\}Ag(C_2H_2)][SbF_6]$) or Photon II ($[Ph_2B(3-(CF_3)Pz)_2]Cu(C_2H_2)$, $[HB(3,5-(CF_3)_2Pz)_3]Cu(C_2H_2)$) detector equipped with an Oxford Cryosystems 700 series cooler, a Triumph monochromator, and a Mo Kα fine-focus sealed tube (λ = 0.71073 Å). Intensity data were processed using the Bruker Apex3 or Apex4 program suite. Absorption corrections were applied by using SADABS. Initial atomic positions were located by direct methods using XT, and the structures of the compounds were refined by the least-squares method using SHELXL[6, 7] within Olex2[8] GUI. The $[HB(3,5-(CF_3)_2Pz)_3]Cu(C_2H_2)$ crystallized with two molecules of toluene and sits on a crystallographic mirror plane containing B, Cu, a pyrazolyl ring, and centroid of the acetylene ligand. All the non-hydrogen atoms were refined anisotropically. Hydrogen atoms were included at calculated positions and refined riding on corresponding carbons, except hydrogen atoms on acetylene carbons of $[Ph_2B(3-(CF_3)Pz)_2]Cu(C_2H_2)$, $[Ph_2B(3-(CF_3)Pz)_2]Ag(C_2H_2)$, and $[\{H_2C(3,5-(Me)_2Pz)_2\}Cu(C_2H_2)][BF_4]$, which were located from difference map and refined freely. X-ray structural figures were generated using Olex2. CCDC **2152321-2152325** files contain the supplementary crystallographic data. These data can be obtained free of charge via http://www.ccdc.cam.ac.uk/conts/retrieving.html or from the Cambridge Crystallographic Data Centre (CCDC), 12 Union Road, Cambridge, CB2 1EZ, UK). Further details are given in the CIF.

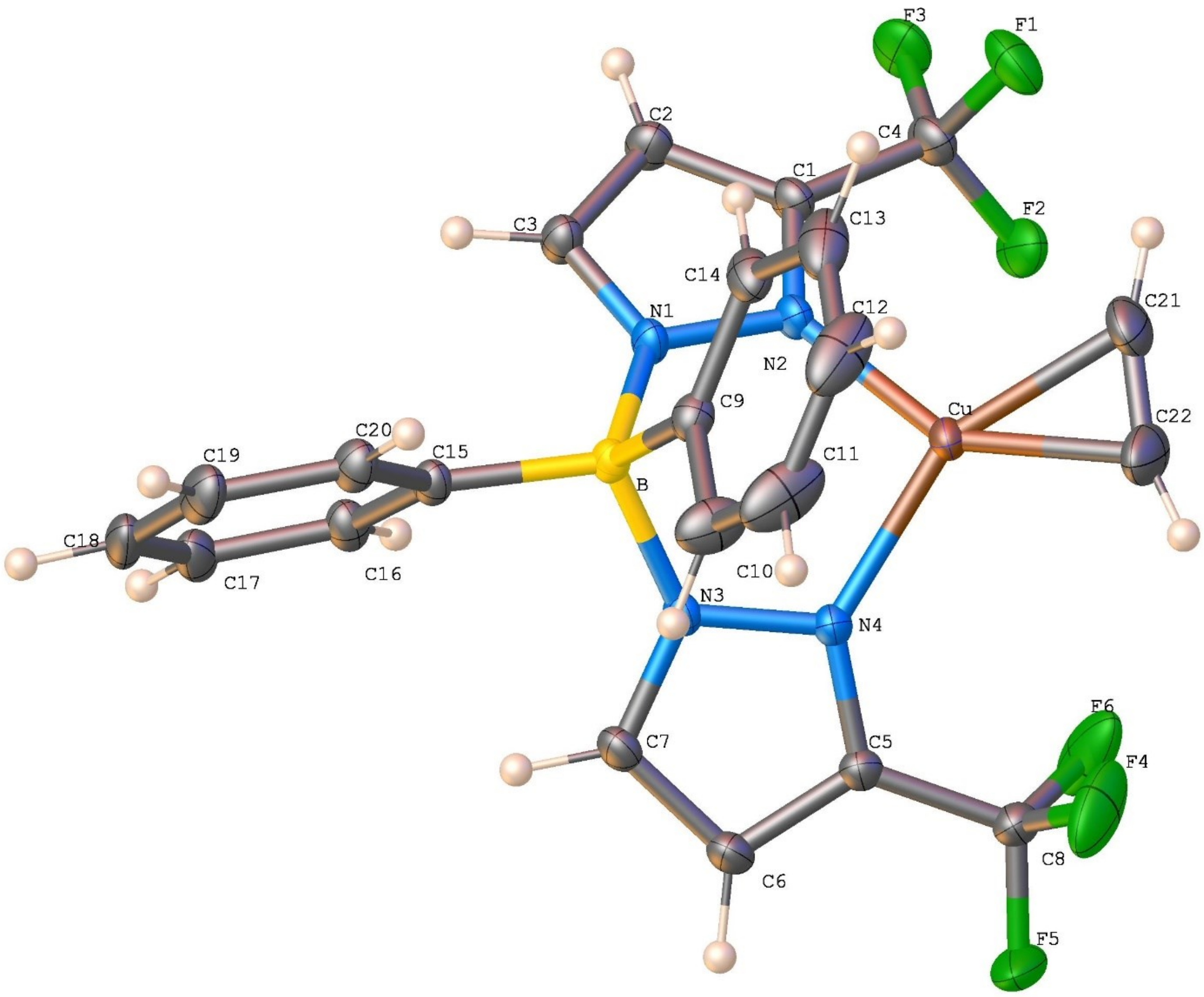

Figure S33: X-ray crystal structure and atom labelling of $[Ph_2B(3\text{-}(CF_3)Pz)_2]Cu(C_2H_2)$ **(9)**.

Table S2: Crystal data and structure refinement for $[Ph_2B(3\text{-}(CF_3)Pz)_2]Cu(C_2H_2)$ **(9)**.

Identification code	hrd16
Empirical formula	$C_{22}H_{16}BCuF_6N_4$
Formula weight	524.74
Temperature/K	100.0
Crystal system	triclinic
Space group	P-1
a/Å	10.1272(2)
b/Å	10.3873(2)
c/Å	12.0427(3)

α/°	75.5420(10)
β/°	65.6390(10)
γ/°	76.9360(10)
Volume/Å^3	1106.56(4)
Z	2
ρ_{calc}g/cm^3	1.575
μ/mm^{-1}	1.055
F(000)	528.0
Crystal size/mm^3	0.33 × 0.3 × 0.19
Radiation	MoKα (λ = 0.71073)
2Θ range for data collection/°	5.256 to 66.282
Index ranges	-15 ≤ h ≤ 15, -15 ≤ k ≤ 15, -18 ≤ l ≤ 17
Reflections collected	21089
Independent reflections	8239 [R_{int} = 0.0152, R_{sigma} = 0.0175]
Data/restraints/parameters	8239/0/316
Goodness-of-fit on F^2	1.077
Final R indexes [I>=2σ (I)]	R_1 = 0.0331, wR_2 = 0.0824
Final R indexes [all data]	R_1 = 0.0373, wR_2 = 0.0857
Largest diff. peak/hole / e Å^{-3}	1.21/-0.86

Table S3: Bond Lengths for [$Ph_2B(3-(CF_3)Pz)_2$]Cu(C_2H_2) **(9)**.

Atom	Atom	Length/Å	Atom	Atom	Length/Å
Cu	N2	1.9714(10)	C2	C3	1.3879(17)
Cu	N4	1.9697(10)	C5	C6	1.3899(17)
Cu	C21	1.9629(14)	C5	C8	1.4874(17)
Cu	C22	1.9567(15)	C6	C7	1.3833(18)
F1	C4	1.3460(17)	C9	C10	1.4032(19)
F2	C4	1.3373(16)	C9	C14	1.3993(18)
F3	C4	1.3374(16)	C9	B	1.6153(18)
F4	C8	1.3332(17)	C10	C11	1.389(2)
F5	C8	1.3154(15)	C11	C12	1.386(3)
F6	C8	1.3106(17)	C12	C13	1.380(3)

Table S3: Bond Lengths for $[Ph_2B(3\text{-}(CF_3)Pz)_2]Cu(C_2H_2)$ **(9)**.

Atom	Atom	Length/Å	Atom	Atom	Length/Å
N1	N2	1.3602(13)	C13	C14	1.399(2)
N1	C3	1.3431(15)	C15	C16	1.4053(17)
N1	B	1.5767(16)	C15	C20	1.3998(17)
N2	C1	1.3404(15)	C15	B	1.6168(17)
N3	N4	1.3602(13)	C16	C17	1.3931(18)
N3	C7	1.3467(15)	C17	C18	1.389(2)
N3	B	1.5900(16)	C18	C19	1.383(2)
N4	C5	1.3415(15)	C19	C20	1.3970(18)
C1	C2	1.3885(17)	C21	C22	1.217(3)
C1	C4	1.4877(17)			

Table S4: Bond Angles for $[Ph_2B(3\text{-}(CF_3)Pz)_2]Cu(C_2H_2)$ **(9)**.

Atom	Atom	Atom	Angle/°	Atom	Atom	Atom	Angle/°
N4	Cu	N2	95.51(4)	C7	C6	C5	103.91(11)
C21	Cu	N2	113.32(6)	N3	C7	C6	109.11(11)
C21	Cu	N4	151.14(6)	F4	C8	C5	112.37(12)
C22	Cu	N2	149.48(6)	F5	C8	F4	105.32(13)
C22	Cu	N4	115.01(6)	F5	C8	C5	111.48(11)
C22	Cu	C21	36.17(8)	F6	C8	F4	105.34(14)
N2	N1	B	120.21(9)	F6	C8	F5	108.66(14)
C3	N1	N2	109.77(9)	F6	C8	C5	113.18(11)
C3	N1	B	129.51(10)	C10	C9	B	119.42(11)
N1	N2	Cu	117.47(7)	C14	C9	C10	116.92(12)
C1	N2	Cu	136.29(8)	C14	C9	B	123.66(11)
C1	N2	N1	105.93(10)	C11	C10	C9	121.89(16)
N4	N3	B	119.69(9)	C12	C11	C10	119.87(17)
C7	N3	N4	109.55(10)	C13	C12	C11	119.80(15)
C7	N3	B	130.03(10)	C12	C13	C14	120.11(16)
N3	N4	Cu	117.00(7)	C13	C14	C9	121.39(15)

Table S4: Bond Angles for $[Ph_2B(3\text{-}(CF_3)Pz)_2]Cu(C_2H_2)$ **(9)**.

Atom	Atom	Atom	Angle/°	Atom	Atom	Atom	Angle/°
C5	N4	Cu	136.56(8)	C16	C15	B	121.66(10)
C5	N4	N3	106.15(9)	C20	C15	C16	116.80(11)
N2	C1	C2	111.57(10)	C20	C15	B	121.48(11)
N2	C1	C4	120.97(11)	C17	C16	C15	121.98(12)
C2	C1	C4	127.45(11)	C18	C17	C16	119.63(13)
C3	C2	C1	103.65(11)	C19	C18	C17	119.84(12)
N1	C3	C2	109.07(11)	C18	C19	C20	120.12(13)
F1	C4	C1	112.65(11)	C19	C20	C15	121.57(13)
F2	C4	F1	106.27(12)	C22	C21	Cu	71.64(10)
F2	C4	C1	112.98(11)	C21	C22	Cu	72.20(10)
F3	C4	F1	106.57(11)	N1	B	N3	106.61(9)
F3	C4	F2	107.52(11)	N1	B	C9	109.10(9)
F3	C4	C1	110.49(11)	N1	B	C15	109.98(9)
N4	C5	C6	111.28(11)	N3	B	C9	106.44(9)
N4	C5	C8	121.14(10)	N3	B	C15	108.36(9)
C6	C5	C8	127.58(11)	C9	B	C15	115.90(9)

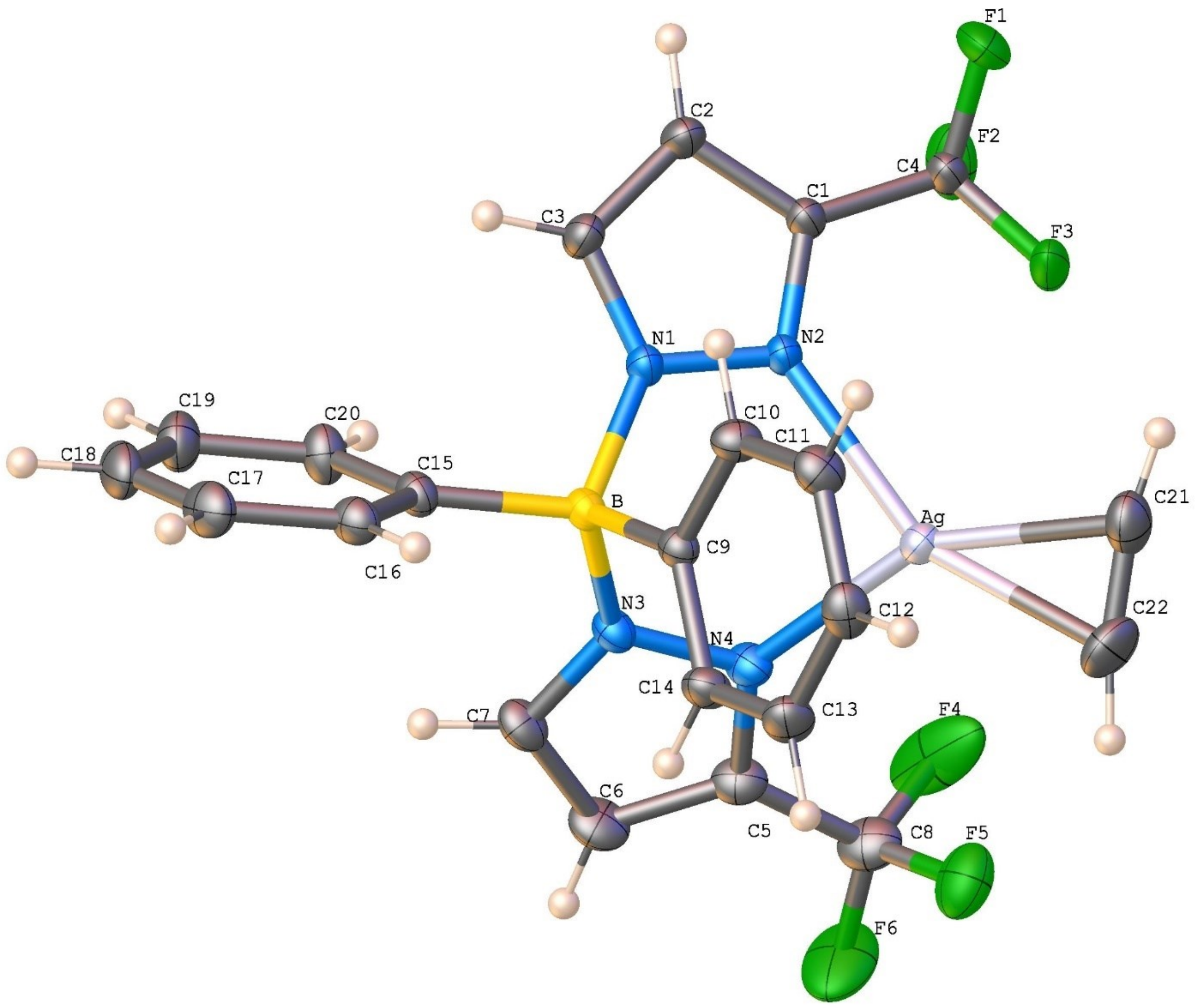

Figure S34: X-ray crystal structure and atom labelling of $[Ph_2B(3\text{-}(CF_3)Pz)_2]Ag(C_2H_2)$ **(10)**.

Table S5: Crystal data and structure refinement for $[Ph_2B(3\text{-}(CF_3)Pz)_2]Ag(C_2H_2)$ **(10)**.

Identification code	Rad830
Empirical formula	$C_{22}H_{16}AgBF_6N_4$
Formula weight	569.07
Temperature/K	100.0
Crystal system	monoclinic
Space group	$P2_1/n$
a/Å	14.6979(6)
b/Å	10.2199(5)
c/Å	15.7068(7)

α/°	90
β/°	105.8120(10)
γ/°	90
Volume/Å^3	2270.06(18)
Z	4
ρ_{calc}g/cm^3	1.665
μ/mm^{-1}	0.955
F(000)	1128.0
Crystal size/mm^3	0.36 × 0.29 × 0.04
Radiation	MoKα (λ = 0.71073)
2Θ range for data collection/°	5.392 to 72.634
Index ranges	-24 ≤ h ≤ 24, -17 ≤ k ≤ 16, -26 ≤ l ≤ 26
Reflections collected	41532
Independent reflections	10964 [R_{int} = 0.0350, R_{sigma} = 0.0325]
Data/restraints/parameters	10964/0/316
Goodness-of-fit on F^2	1.053
Final R indexes [I>=2σ (I)]	R_1 = 0.0354, wR_2 = 0.0748
Final R indexes [all data]	R_1 = 0.0501, wR_2 = 0.0812
Largest diff. peak/hole / e Å^{-3}	0.85/-0.84

Table S6: Bond Lengths for [$Ph_2B(3\text{-}(CF_3)Pz)_2$]Ag($C_2H_2$) **(10)**.

Atom	Atom	Length/Å	Atom	Atom	Length/Å
Ag	N2	2.2665(12)	C2	C3	1.382(2)
Ag	N4	2.2415(14)	C5	C6	1.389(3)
Ag	C21	2.2653(19)	C5	C8	1.483(2)
Ag	C22	2.2531(19)	C6	C7	1.382(2)
F1	C4	1.335(2)	C9	C10	1.403(2)
F2	C4	1.351(2)	C9	C14	1.403(2)

Table S6: Bond Lengths for $[Ph_2B(3-(CF_3)Pz)_2]Ag(C_2H_2)$ **(10)**.

Atom	Atom	Length/Å	Atom	Atom	Length/Å
F3	C4	1.3324(19)	C9	B	1.619(2)
F4	C8	1.329(2)	C10	C11	1.392(2)
F5	C8	1.328(2)	C11	C12	1.389(2)
F6	C8	1.333(2)	C12	C13	1.393(2)
N1	N2	1.3551(17)	C13	C14	1.389(2)
N1	C3	1.3489(19)	C15	C16	1.397(2)
N1	B	1.584(2)	C15	C20	1.405(2)
N2	C1	1.3342(19)	C15	B	1.615(2)
N3	N4	1.3583(18)	C16	C17	1.399(2)
N3	C7	1.347(2)	C17	C18	1.382(3)
N3	B	1.586(2)	C18	C19	1.389(3)
N4	C5	1.336(2)	C19	C20	1.392(2)
C1	C2	1.395(2)	C21	C22	1.193(3)
C1	C4	1.485(2)			

Table S7: Bond Angles for $[Ph_2B(3-(CF_3)Pz)_2]Ag(C_2H_2)$ **(10)**.

Atom	Atom	Atom	Angle/°	Atom	Atom	Atom	Angle/°
N4	Ag	N2	82.76(5)	C7	C6	C5	104.02(14)
N4	Ag	C21	154.02(7)	N3	C7	C6	108.85(15)
N4	Ag	C22	123.44(7)	F4	C8	F6	108.03(18)
C21	Ag	N2	123.21(6)	F4	C8	C5	112.66(16)
C22	Ag	N2	153.77(7)	F5	C8	F4	104.98(19)
C22	Ag	C21	30.63(8)	F5	C8	F6	106.04(17)
N2	N1	B	119.76(11)	F5	C8	C5	113.42(14)
C3	N1	N2	109.65(12)	F6	C8	C5	111.24(17)
C3	N1	B	129.84(12)	C10	C9	C14	116.24(14)
N1	N2	Ag	115.42(9)	C10	C9	B	121.26(13)
C1	N2	Ag	136.04(10)	C14	C9	B	122.49(13)
C1	N2	N1	106.19(12)	C11	C10	C9	122.38(15)

Table S7: Bond Angles for $[Ph_2B(3-(CF_3)Pz)_2]Ag(C_2H_2)$ **(10)**.

Atom	Atom	Atom	Angle/°	Atom	Atom	Atom	Angle/°
N4	N3	B	120.18(12)	C12	C11	C10	119.89(15)
C7	N3	N4	109.64(13)	C11	C12	C13	119.18(15)
C7	N3	B	129.43(13)	C14	C13	C12	120.24(15)
N3	N4	Ag	115.63(9)	C13	C14	C9	122.06(15)
C5	N4	Ag	137.27(12)	C16	C15	C20	116.71(14)
C5	N4	N3	106.19(13)	C16	C15	B	121.37(14)
N2	C1	C2	111.53(13)	C20	C15	B	121.92(13)
N2	C1	C4	119.53(13)	C15	C16	C17	121.43(16)
C2	C1	C4	128.88(14)	C18	C17	C16	120.34(16)
C3	C2	C1	103.52(13)	C17	C18	C19	119.80(16)
N1	C3	C2	109.09(13)	C18	C19	C20	119.33(17)
F1	C4	F2	105.32(14)	C19	C20	C15	122.38(16)
F1	C4	C1	111.88(14)	C22	C21	Ag	74.12(13)
F2	C4	C1	112.43(14)	C21	C22	Ag	75.25(13)
F3	C4	F1	108.44(14)	N1	B	N3	107.70(11)
F3	C4	F2	105.84(14)	N1	B	C9	107.68(12)
F3	C4	C1	112.48(13)	N1	B	C15	108.87(12)
N4	C5	C6	111.29(15)	N3	B	C9	107.41(12)
N4	C5	C8	119.62(16)	N3	B	C15	109.20(12)
C6	C5	C8	129.09(15)	C15	B	C9	115.70(12)

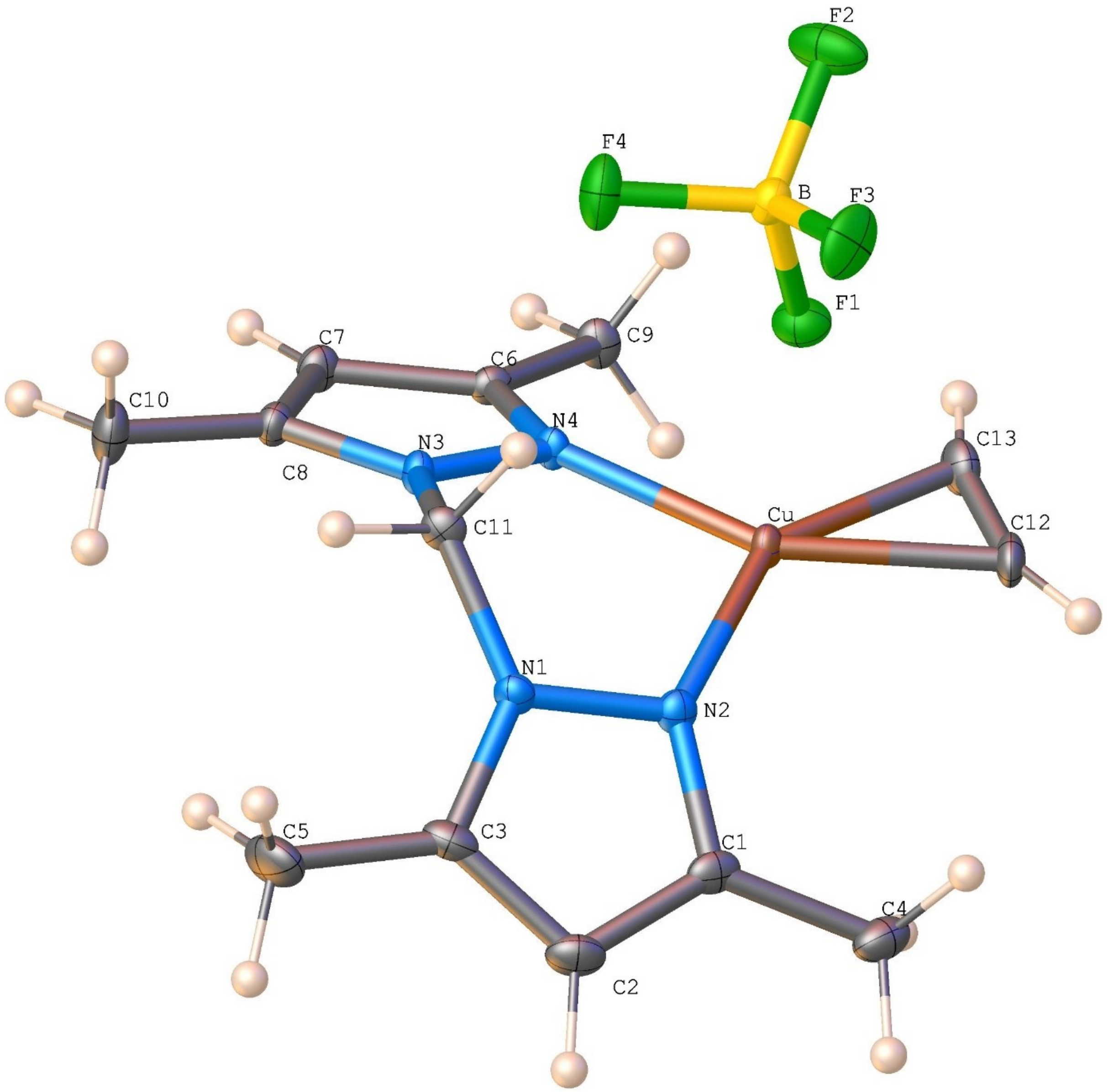

Figure S35: X-ray crystal structure and atom labelling of [{$H_2C(3,5\text{-}(Me)_2Pz)_2$}$Cu(C_2H_2)$][$BF_4$] **(11)**.

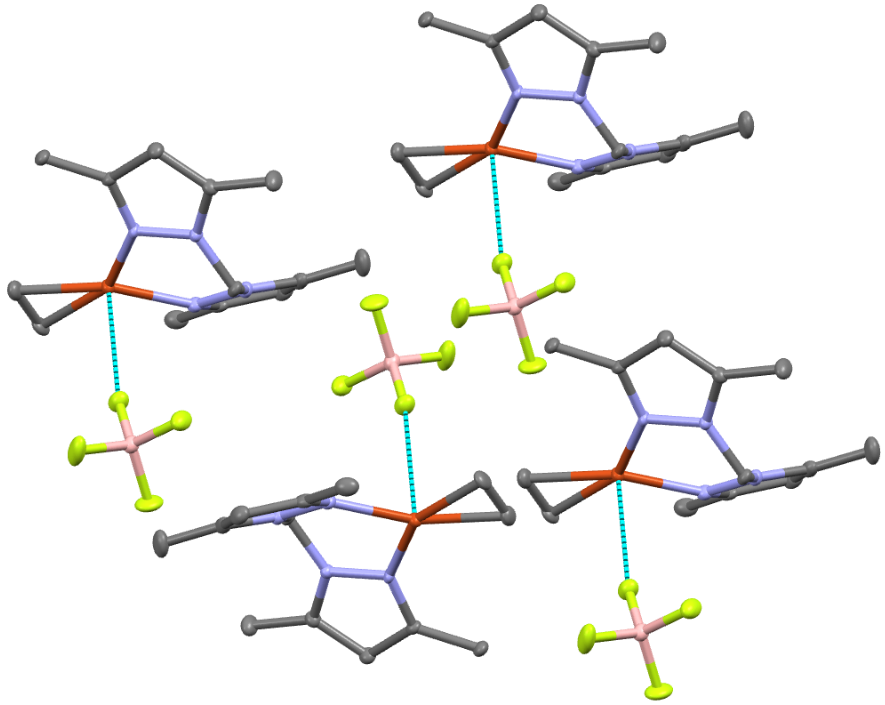

Figure S36: A view of the crystal packing diagram of [{$H_2C(3,5-(Me)_2Pz)_2$}$Cu(C_2H_2)$][BF_4] (**11**) showing fluorine atoms of [BF_4]$^-$ close to copper sites.

Table S8: Crystal data and structure refinement for [{$H_2C(3,5-(Me)_2Pz)_2$}$Cu(C_2H_2)$][BF_4] **(11)**.

Identification code	rad840
Empirical formula	$C_{13}H_{18}BCuF_4N_4$
Formula weight	380.66
Temperature/K	100.0
Crystal system	triclinic
Space group	P-1
a/Å	7.9267(5)
b/Å	10.4752(7)
c/Å	10.6827(7)
α/°	107.079(2)
β/°	100.3190(10)
γ/°	102.0130(10)
Volume/Å^3	801.49(9)

Z	2
ρ_{calc}g/cm^3	1.577
μ/mm^{-1}	1.405
F(000)	388.0
Crystal size/mm^3	0.2 × 0.16 × 0.02
Radiation	Mo Kα (λ = 0.71073)
2Θ range for data collection/°	5.436 to 58.256
Index ranges	-10 ≤ h ≤ 10, -14 ≤ k ≤ 14, -14 ≤ l ≤ 14
Reflections collected	27886
Independent reflections	4311 [R_{int} = 0.0609, R_{sigma} = 0.0442]
Data/restraints/parameters	4311/0/220
Goodness-of-fit on F^2	1.072
Final R indexes [I>=2σ (I)]	R_1 = 0.0492, wR_2 = 0.0855
Final R indexes [all data]	R_1 = 0.0678, wR_2 = 0.0931
Largest diff. peak/hole / e Å^{-3}	0.68/-0.64

Table S9: Bond Lengths for [{$H_2C(3,5-(Me)_2Pz)_2$}Cu(C_2H_2)][BF_4] **(11)**.

Atom	Atom	Length/Å	Atom	Atom	Length/Å
Cu	N4	1.978(2)	N1	C11	1.449(3)
Cu	N2	1.977(2)	N4	C6	1.342(3)
Cu	C13	1.970(3)	N2	C1	1.341(4)
Cu	C12	1.971(3)	C13	C12	1.203(4)
F1	B	1.394(4)	C9	C6	1.488(4)
F3	B	1.387(3)	C6	C7	1.397(4)
F2	B	1.387(4)	C8	C7	1.377(4)
F4	B	1.387(3)	C8	C10	1.486(4)
N3	N4	1.372(3)	C1	C2	1.394(4)
N3	C8	1.358(3)	C1	C4	1.490(4)

Table S9: Bond Lengths for [{$H_2C(3,5-(Me)_2Pz)_2$}$Cu(C_2H_2)$][BF_4] **(11)**.

Atom	Atom	Length/Å	Atom	Atom	Length/Å
N3	C11	1.445(3)	C3	C2	1.372(4)
N1	N2	1.367(3)	C3	C5	1.490(4)
N1	C3	1.358(3)			

Table S10: Bond Angles for [{$H_2C(3,5-(Me)_2Pz)_2$}$Cu(C_2H_2)$][BF_4] **(11)**.

Atom	Atom	Atom	Angle/˚	Atom	Atom	Atom	Angle/˚
N2	Cu	N4	97.14(9)	N4	C6	C7	109.9(2)
C13	Cu	N4	114.63(11)	C7	C6	C9	128.0(2)
C13	Cu	N2	147.43(11)	N3	C8	C7	106.2(2)
C13	Cu	C12	35.55(13)	N3	C8	C10	122.8(2)
C12	Cu	N4	150.11(11)	C7	C8	C10	131.0(3)
C12	Cu	N2	112.67(11)	N2	C1	C2	109.7(3)
N4	N3	C11	119.5(2)	N2	C1	C4	121.3(3)
C8	N3	N4	111.4(2)	C2	C1	C4	129.0(3)
C8	N3	C11	128.8(2)	N1	C3	C2	106.2(2)
N2	N1	C11	117.8(2)	N1	C3	C5	123.4(3)
C3	N1	N2	111.3(2)	C2	C3	C5	130.4(3)
C3	N1	C11	130.8(2)	N3	C11	N1	111.2(2)
N3	N4	Cu	117.81(16)	C3	C2	C1	107.1(3)
C6	N4	Cu	135.33(18)	C8	C7	C6	106.9(2)
C6	N4	N3	105.6(2)	F3	B	F1	110.5(2)
N1	N2	Cu	117.55(17)	F3	B	F2	109.7(3)
C1	N2	Cu	131.1(2)	F3	B	F4	108.7(2)
C1	N2	N1	105.8(2)	F2	B	F1	109.2(2)
C12	C13	Cu	72.24(19)	F2	B	F4	109.6(2)
C13	C12	Cu	72.20(19)	F4	B	F1	109.0(2)

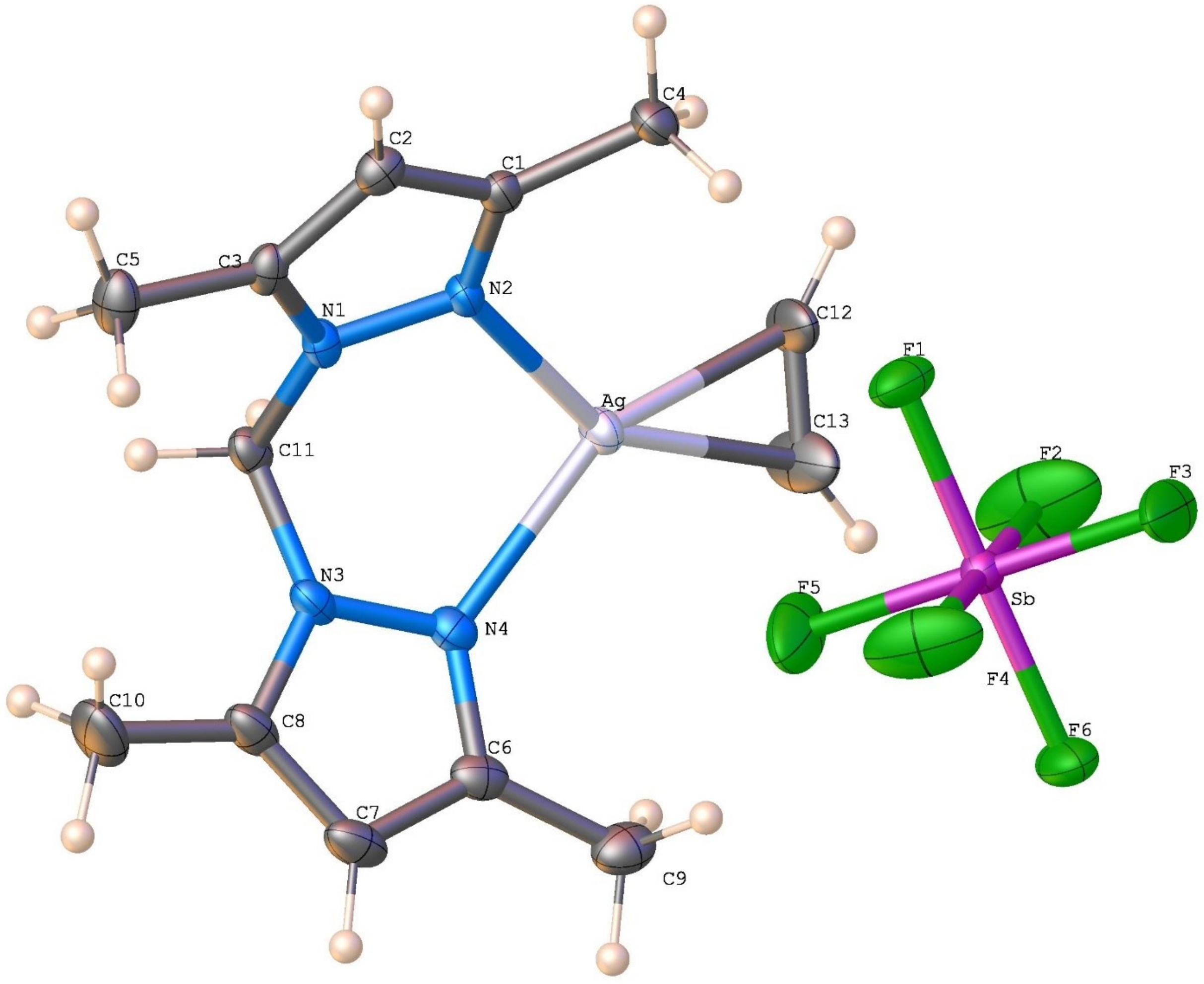

Figure S37: X-ray crystal structure and atom labelling of [{$H_2C(3,5\text{-}(Me)_2Pz)_2$}$Ag(C_2H_2)$][$SbF_6$] **(12)**.

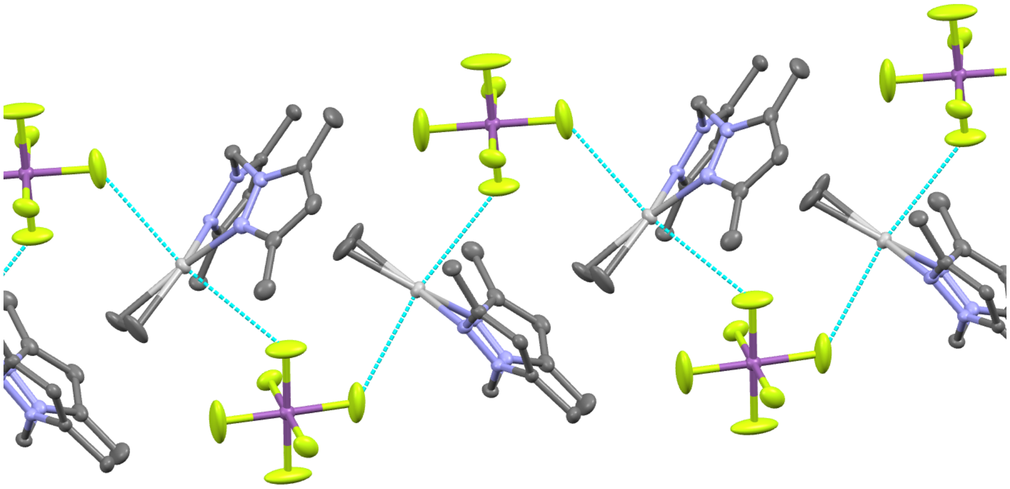

Figure S38: A view of the crystal packing diagram of [{$H_2C(3,5-(Me)_2Pz)_2$}Ag(C_2H_2)][SbF_6] (**12**) showing fluorine atoms of [SbF_6]$^-$ close to silver sites.

Table S11: Crystal data and structure refinement for [{$H_2C(3,5-(Me)_2Pz)_2$}Ag(C_2H_2)][SbF_6] **(12)**.

Identification code	rad838
Empirical formula	$C_{13}H_{18}AgF_6N_4Sb$
Formula weight	573.93
Temperature/K	100.0
Crystal system	monoclinic
Space group	$P2_1/n$
a/Å	11.7240(5)
b/Å	12.2179(5)
c/Å	12.8999(6)
α/°	90
β/°	93.091(2)
γ/°	90
Volume/Å^3	1845.13(14)
Z	4
ρ_{calc}g/cm^3	2.066
μ/mm^{-1}	2.586

F(000)	1104.0
Crystal size/mm^3	0.46 × 0.36 × 0.17
Radiation	Mo Kα (λ = 0.71073)
2Θ range for data collection/°	4.818 to 64.06
Index ranges	-17 ≤ h ≤ 17, -18 ≤ k ≤ 18, -19 ≤ l ≤ 19
Reflections collected	89480
Independent reflections	6436 [R_{int} = 0.0430, R_{sigma} = 0.0168]
Data/restraints/parameters	6436/0/231
Goodness-of-fit on F^2	1.022
Final R indexes [I>=2σ (I)]	R_1 = 0.0352, wR_2 = 0.0678
Final R indexes [all data]	R_1 = 0.0429, wR_2 = 0.0744
Largest diff. peak/hole / e Å^{-3}	1.65/-1.44

Table S12: Bond Lengths for [{$H_2C(3,5-(Me)_2Pz)_2$}Ag(C_2H_2)][SbF_6] **(12)**.

Atom	Atom	Length/Å	Atom	Atom	Length/Å
Sb	F1	1.867(2)	N3	N4	1.366(4)
Sb	F2	1.853(3)	N3	C8	1.360(4)
Sb	F3	1.857(2)	N3	C11	1.448(4)
Sb	F4	1.865(3)	N4	C6	1.335(4)
Sb	F5	1.862(2)	C1	C2	1.404(4)
Sb	F6	1.874(2)	C1	C4	1.492(4)
Ag	N2	2.220(2)	C2	C3	1.381(4)
Ag	N4	2.235(3)	C3	C5	1.494(4)
Ag	C12	2.251(3)	C6	C7	1.407(5)
Ag	C13	2.237(4)	C6	C9	1.491(5)
N1	N2	1.366(3)	C7	C8	1.375(5)
N1	C3	1.355(4)	C8	C10	1.487(5)
N1	C11	1.444(4)	C12	C13	1.203(5)
N2	C1	1.334(4)			

Table S13: Bond Angles for [{$H_2C(3,5\text{-}(Me)_2Pz)_2$}Ag($C_2H_2$)][$SbF_6$] **(12)**.

Atom	Atom	Atom	Angle/°	Atom	Atom	Atom	Angle/°
F1	Sb	F6	178.14(11)	C1	N2	Ag	132.80(19)
F2	Sb	F1	90.72(13)	C1	N2	N1	106.0(2)
F2	Sb	F3	90.3(2)	N4	N3	C11	118.6(2)
F2	Sb	F4	179.1(2)	C8	N3	N4	111.4(3)
F2	Sb	F5	89.8(2)	C8	N3	C11	130.0(3)
F2	Sb	F6	90.40(13)	N3	N4	Ag	117.77(18)
F3	Sb	F1	89.72(11)	C6	N4	Ag	133.7(2)
F3	Sb	F4	90.6(2)	C6	N4	N3	106.0(3)
F3	Sb	F5	179.92(19)	N2	C1	C2	110.1(3)
F3	Sb	F6	88.79(12)	N2	C1	C4	120.3(3)
F4	Sb	F1	89.56(12)	C2	C1	C4	129.5(3)
F4	Sb	F6	89.34(12)	C3	C2	C1	106.1(3)
F5	Sb	F1	90.32(11)	N1	C3	C2	106.6(2)
F5	Sb	F4	89.30(18)	N1	C3	C5	123.0(3)
F5	Sb	F6	91.18(11)	C2	C3	C5	130.3(3)
N2	Ag	N4	88.66(9)	N4	C6	C7	109.8(3)
N2	Ag	C12	121.48(12)	N4	C6	C9	121.2(3)
N2	Ag	C13	151.67(13)	C7	C6	C9	129.0(3)
N4	Ag	C12	149.78(12)	C8	C7	C6	106.5(3)
N4	Ag	C13	119.27(13)	N3	C8	C7	106.3(3)
C13	Ag	C12	31.10(14)	N3	C8	C10	123.0(3)
N2	N1	C11	117.8(2)	C7	C8	C10	130.7(3)
C3	N1	N2	111.3(2)	N1	C11	N3	112.8(2)
C3	N1	C11	130.8(2)	C13	C12	Ag	73.8(2)
N1	N2	Ag	118.89(17)	C12	C13	Ag	75.1(2)

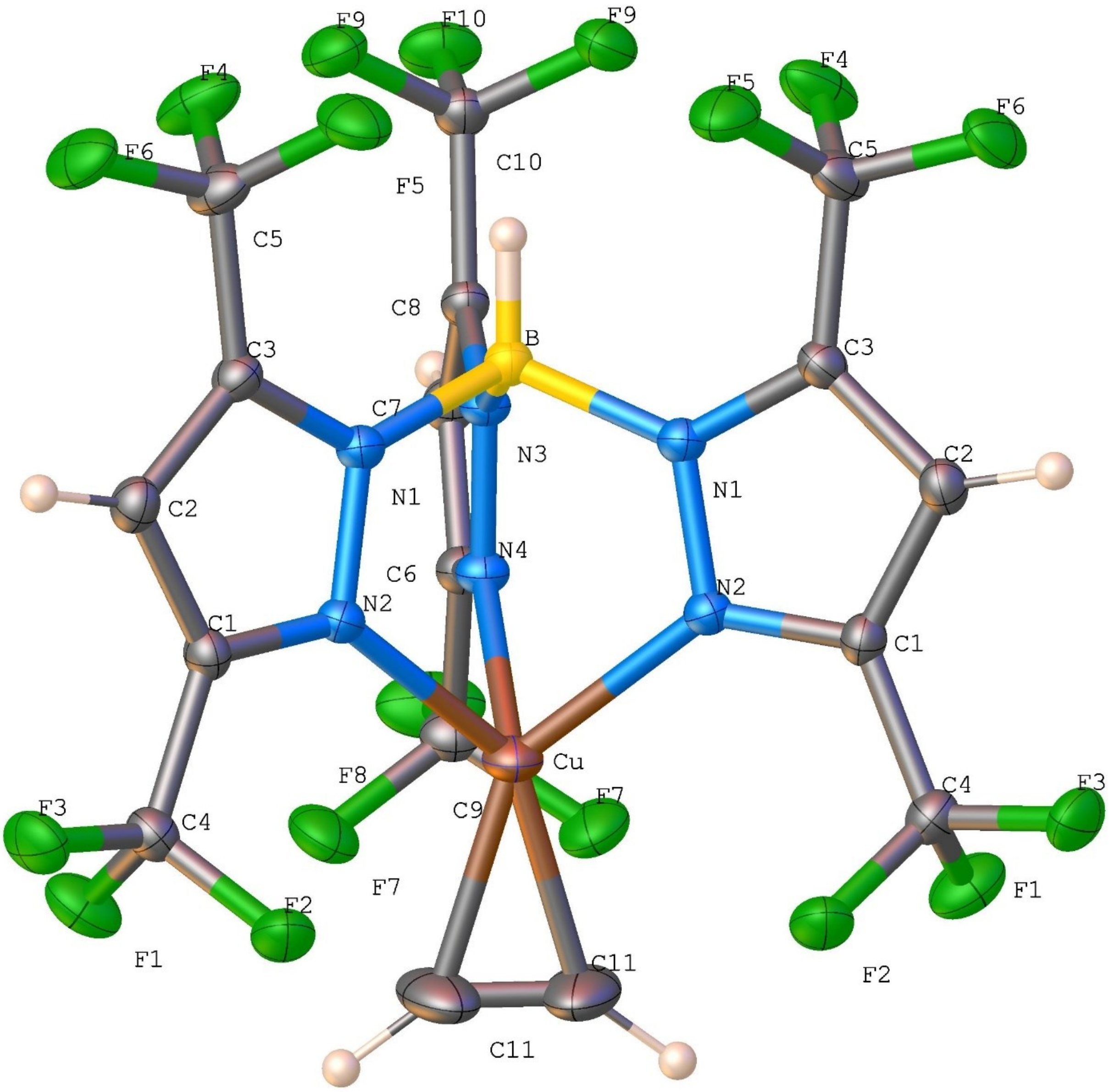
F9
F10
F9
F4
F5
F4
F6
F5
F6
C5
C10
C5
C5
C8
B
C3
C3
C7
N3
C2
N1
N1
C2
N4
C6
N2
N2
C1
C1
Cu
F8
F7
C4
C9
F3
C4
F3
F2
F7
F1
F1
C11
F2
C11

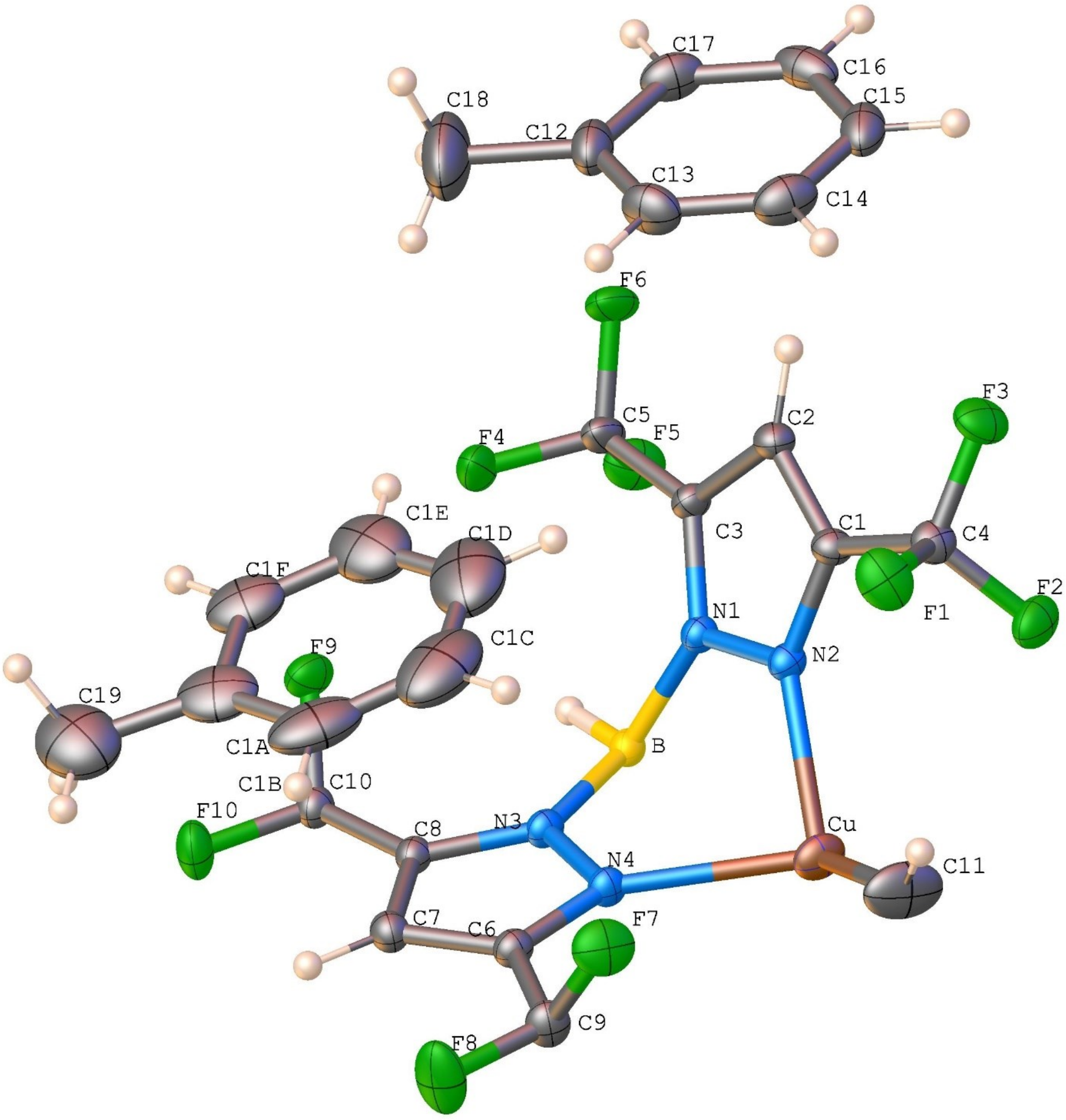

Figure S39: X-ray crystal structure and atom labelling of the asymmetric unit of [HB(3,5-$(CF_3)_2$Pz$)_3$]Cu(C_2H_2) **(13)** with toluene present in the crystal lattice.

Table S14: Crystal data and structure refinement for [HB(3,5-$(CF_3)_2$Pz$)_3$]Cu(C_2H_2) **(13)**.

Identification code	hrd36
Empirical formula	$C_{31}H_{22}BCuF_{18}N_6$
Formula weight	894.89

Temperature/K	100.0
Crystal system	monoclinic
Space group	$P2_1/m$
a/Å	11.0640(5)
b/Å	13.6074(6)
c/Å	11.4278(5)
α/°	90
β/°	91.989(2)
γ/°	90
Volume/Å^3	1719.44(13)
Z	2
ρ_{calc}g/cm^3	1.728
μ/mm^{-1}	0.768
F(000)	892.0
Crystal size/mm^3	0.27 × 0.25 × 0.03
Radiation	MoKα (λ = 0.71073)
2Θ range for data collection/°	5.038 to 64.058
Index ranges	$-16 \le h \le 16$, $-20 \le k \le 20$, $-16 \le l \le 17$
Reflections collected	30468
Independent reflections	6197 [R_{int} = 0.0333, R_{sigma} = 0.0269]
Data/restraints/parameters	6197/136/319
Goodness-of-fit on F^2	1.039
Final R indexes [I>=2σ (I)]	R_1 = 0.0534, wR_2 = 0.1104
Final R indexes [all data]	R_1 = 0.0842, wR_2 = 0.1365
Largest diff. peak/hole / e Å^{-3}	1.39/-1.48

Table S15: Bond Lengths for [HB(3,5-$(CF_3)_2$Pz$)_3$]Cu(C_2H_2) **(13)**.

Atom	Atom	Length/Å	Atom	Atom	Length/Å
Cu	N2[1]	2.0466(17)	C2	C1	1.398(3)
Cu	N2	2.0466(17)	C2	C3	1.378(3)
Cu	N4	2.179(3)	C1	C4	1.493(3)
Cu	C11[1]	1.986(3)	C6	C7	1.390(4)
Cu	C11	1.986(3)	C6	C9	1.501(4)
F9	C10	1.336(2)	C7	C8	1.370(4)
F3	C4	1.330(3)	C3	C5	1.495(3)
F6	C5	1.336(3)	C8	C10	1.492(4)
F2	C4	1.338(3)	C13	C12	1.396(6)
F10	C10	1.340(4)	C13	C14	1.377(6)
F1	C4	1.344(3)	C12	C17	1.385(6)
F5	C5	1.339(3)	C12	C18	1.506(6)
F7	C9	1.334(2)	C14	C15	1.375(6)
F4	C5	1.334(3)	C17	C16	1.384(6)
F8	C9	1.328(4)	C15	C16	1.374(6)
N2	N1	1.358(2)	C11	C11[1]	1.134(7)
N2	C1	1.332(3)	C19	C1A	1.501(10)
N3	N4	1.361(3)	C1A	C1B	1.381(8)
N3	C8	1.362(4)	C1A	C1F	1.371(8)
N3	B	1.552(4)	C1B	C1C	1.393(9)
N4	C6	1.330(4)	C1C	C1D	1.425(9)
N1	C3	1.357(3)	C1D	C1E	1.330(9)
N1	B	1.565(3)	C1E	C1F	1.392(9)

[1]+X,3/2-Y,+Z

Table S16: Bond Angles for [HB(3,5-$(CF_3)_2$Pz$)_3$]Cu(C_2H_2) **(13)**.

Atom	Atom	Atom	Angle/°	Atom	Atom	Atom	Angle/°
N2[1]	Cu	N2	90.17(10)	N3	C8	C10	124.1(3)
N2	Cu	N4	88.25(7)	C7	C8	C10	127.0(3)

Table S16: Bond Angles for $[HB(3,5\text{-}(CF_3)_2Pz)_3]Cu(C_2H_2)$ **(13)**.

Atom	Atom	Atom	Angle/°	Atom	Atom	Atom	Angle/°
N2[1]	Cu	N4	88.25(7)	F9[1]	C10	F9	106.5(2)
C11	Cu	N2[1]	144.71(12)	F9[1]	C10	F10	107.06(17)
C11[1]	Cu	N2[1]	114.34(10)	F9	C10	F10	107.06(17)
C11[1]	Cu	N2	144.71(12)	F9[1]	C10	C8	113.13(16)
C11	Cu	N2	114.34(10)	F9	C10	C8	113.13(16)
C11	Cu	N4	116.08(13)	F10	C10	C8	109.6(3)
C11[1]	Cu	N4	116.08(13)	C14	C13	C12	120.9(4)
C11[1]	Cu	C11	33.16(19)	C13	C12	C18	121.6(5)
N1	N2	Cu	116.83(13)	C17	C12	C13	118.0(3)
C1	N2	Cu	136.69(14)	C17	C12	C18	120.4(5)
C1	N2	N1	106.47(16)	F6	C5	F5	107.49(18)
N4	N3	C8	109.4(2)	F6	C5	C3	109.84(19)
N4	N3	B	118.9(2)	F5	C5	C3	112.44(19)
C8	N3	B	131.7(2)	F4	C5	F6	107.60(19)
N3	N4	Cu	115.61(18)	F4	C5	F5	107.10(19)
C6	N4	Cu	138.7(2)	F4	C5	C3	112.14(19)
C6	N4	N3	105.7(2)	F7	C9	F7[1]	106.4(3)
N2	N1	B	120.48(18)	F7	C9	C6	112.22(17)
C3	N1	N2	108.85(16)	F7[1]	C9	C6	112.22(17)
C3	N1	B	130.67(18)	F8	C9	F7	107.62(18)
C3	C2	C1	103.07(18)	F8	C9	F7[1]	107.62(18)
N2	C1	C2	111.86(18)	F8	C9	C6	110.5(3)
N2	C1	C4	120.35(18)	C15	C14	C13	120.4(4)
C2	C1	C4	127.79(19)	C16	C17	C12	120.7(3)
N4	C6	C7	112.1(3)	C16	C15	C14	119.5(4)
N4	C6	C9	120.0(3)	C15	C16	C17	120.5(3)
C7	C6	C9	127.9(3)	C11[1]	C11	Cu	73.42(9)
C8	C7	C6	103.9(3)	N3	B	N1	108.81(16)
N1	C3	C2	109.76(18)	N3	B	N1[1]	108.81(16)
N1	C3	C5	123.64(19)	N1	B	N1[1]	108.1(2)

Table S16: Bond Angles for [HB(3,5-$(CF_3)_2$Pz$)_3$]Cu(C_2H_2) **(13)**.

Atom	Atom	Atom	Angle/°	Atom	Atom	Atom	Angle/°
C2	C3	C5	126.60(19)	C1B	C1A	C19	129.8(7)
F3	C4	F2	107.51(18)	C1F	C1A	C19	111.7(7)
F3	C4	F1	107.33(18)	C1F	C1A	C1B	118.6(7)
F3	C4	C1	110.99(18)	C1A	C1B	C1C	120.1(8)
F2	C4	F1	106.19(18)	C1B	C1C	C1D	120.8(8)
F2	C4	C1	112.45(18)	C1E	C1D	C1C	116.8(8)
F1	C4	C1	112.06(18)	C1D	C1E	C1F	123.1(8)
N3	C8	C7	108.9(3)	C1A	C1F	C1E	120.6(7)

[1]+X,3/2-Y,+Z

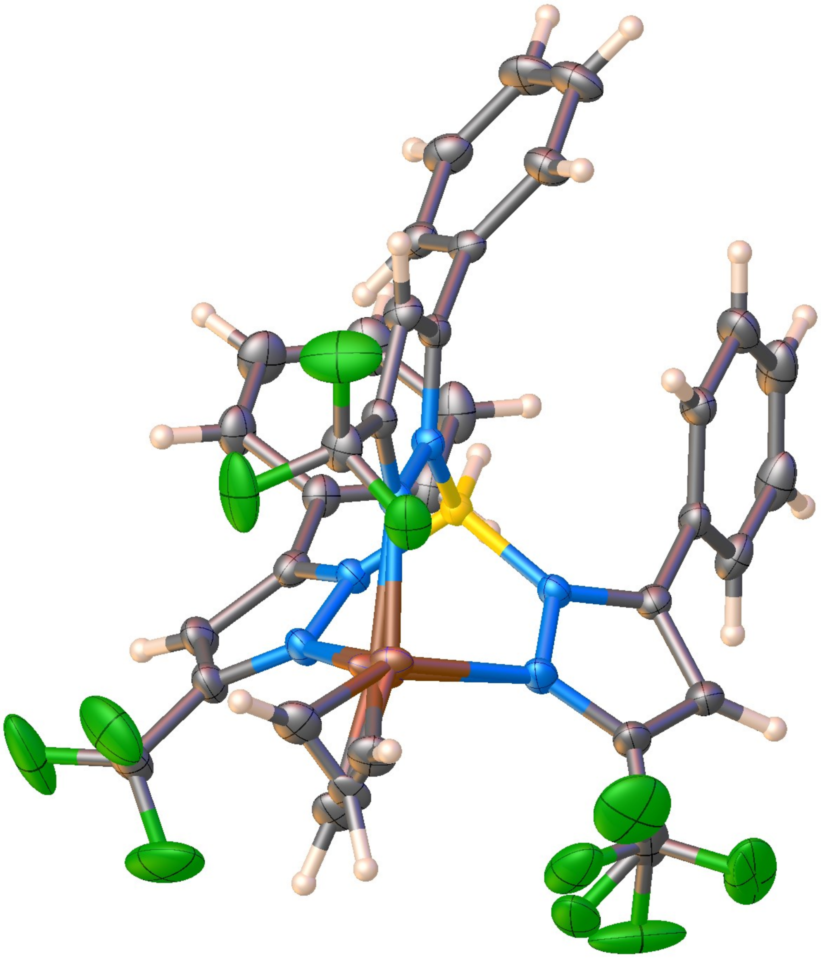

Figure S40: X-ray crystal structure of [HB(3-(CF_3),5-(Ph)Pz)$_3$]Cu(C_2H_2) **(14)**.

Although the atom connectivities are clear, the crystal quality and data of [HB(3-(CF_3),5-(Ph)Pz)$_3$]Cu(C_2H_2) are poor and the copper-acetylene moiety disorder is significant, and therefore, the structure is not suitable for detailed analysis of the metrical parameters

Table S17: Crystal data and structure refinement for [HB(3-(CF_3),5-(Ph)Pz)$_3$]Cu(C_2H_2)•CH_2Cl_2 **(14•CH_2Cl_2)**.

Identification code	rad360_0m_a-DISORD
Empirical formula	$C_{33}H_{23}BCl_2CuF_9N_6$
Formula weight	819.82
Temperature/K	100.05
Crystal system	orthorhombic
Space group	Pbcn

a/Å	23.4988(11)
b/Å	16.9738(8)
c/Å	16.9704(8)
α/°	90
β/°	90
γ/°	90
Volume/Å^3	6768.9(6)
Z	8
ρ_{calc}g/cm^3	1.609
μ/mm^{-1}	0.888
F(000)	3296.0
Crystal size/mm^3	0.23 × 0.2 × 0.07
Radiation	Mo Kα (λ = 0.71073)
2Θ range for data collection/°	5.64 to 53.462
Index ranges	$-29 \le h \le 29$, $-21 \le k \le 21$, $-21 \le l \le 21$
Reflections collected	61854
Independent reflections	6497 [R_{int} = 0.3051, R_{sigma} = 0.2445]
Data/restraints/parameters	6497/103/552
Goodness-of-fit on F^2	0.979
Final R indexes [I>=2σ (I)]	R_1 = 0.0640, wR_2 = 0.1318
Final R indexes [all data]	R_1 = 0.1601, wR_2 = 0.1477
Largest diff. peak/hole / e Å^{-3}	1.52/-1.04

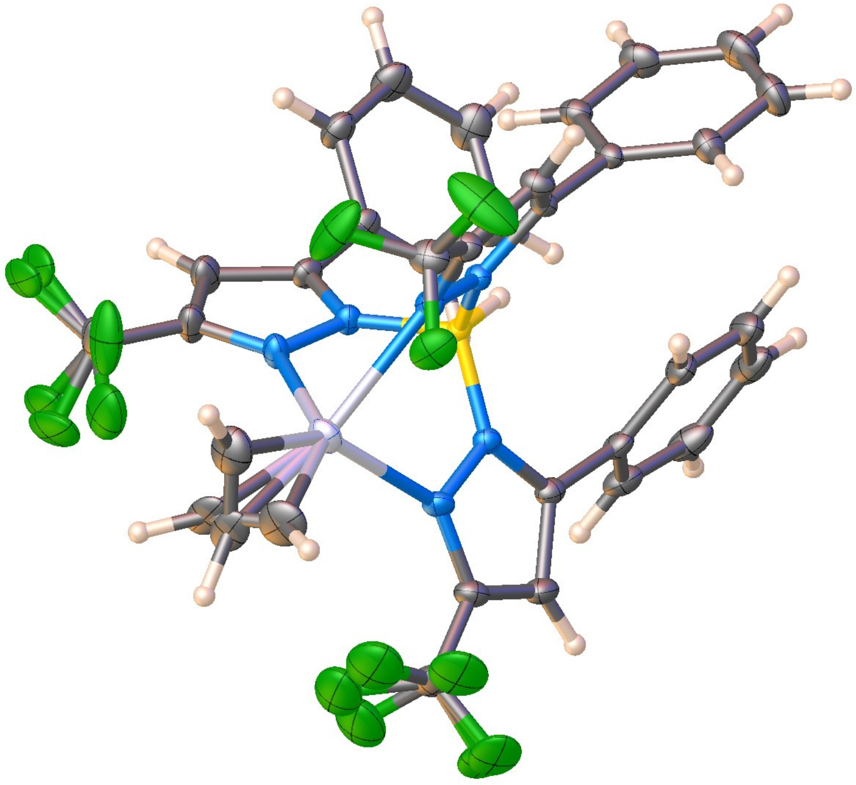

Figure S41: X-ray crystal structure of [HB(3-(CF_3),5-(Ph)Pz)$_3$]Ag(C_2H_2) **(15)**.

Although the atom connectivities are clear and the refinement is reasonably good for most standards, the silver-acetylene moiety disorder is significant, and therefore, the structure is not suitable for detailed analysis of the metrical parameters

Table S18: Crystal data and structure refinement for [HB(3-(CF_3),5-(Ph)Pz)$_3$]Ag(C_2H_2)•CH_2Cl_2 **(15•CH_2Cl_2).**

Identification code	rad356a_Disord
Empirical formula	$C_{33}H_{23}AgBCl_2F_9N_6$
Formula weight	864.15
Temperature/K	99.99
Crystal system	orthorhombic
Space group	Pbcn
a/Å	23.6982(8)
b/Å	16.9056(6)
c/Å	17.0298(6)
α/°	90
β/°	90

γ/°	90
Volume/Å^3	6822.7(4)
Z	8
ρ_{calc}g/cm^3	1.683
μ/mm^{-1}	0.832
F(000)	3440.0
Crystal size/mm^3	0.39 × 0.2 × 0.2
Radiation	Mo Kα (λ = 0.71073)
2Θ range for data collection/°	5.626 to 57.398
Index ranges	$-32 \le h \le 32$, $-22 \le k \le 22$, $-23 \le l \le 23$
Reflections collected	126066
Independent reflections	8813 [R_{int} = 0.0706, R_{sigma} = 0.0213]
Data/restraints/parameters	8813/182/571
Goodness-of-fit on F^2	1.052
Final R indexes [I>=2σ (I)]	R_1 = 0.0467, wR_2 = 0.1245
Final R indexes [all data]	R_1 = 0.0577, wR_2 = 0.1314
Largest diff. peak/hole / e Å^{-3}	2.07/-1.78

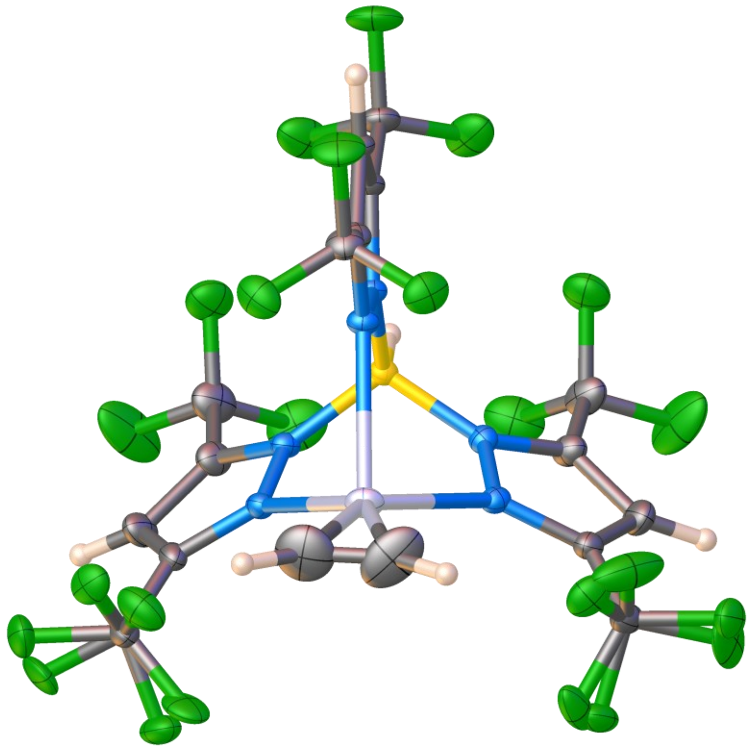

Figure S42: X-ray crystal structure of [HB(3,5-$(CF_3)_2$Pz$)_3$]Ag(C_2H_2) (**5**) at 100 K.

A fresh high-resolution dataset of [HB(3,5-$(CF_3)_2$Pz$)_3$]Ag(C_2H_2) (**5**) has been collected at 100 K (compared to the reported data at 183 K) to see if it is possible to minimize the libration and thermal smearing effects on the acetylene CC bond. However, the improvements were not noticeable as this molecule also suffers from a minor disorder of the acetylene moiety. The acetylene CC distance is 1.126(6) Å. This new dataset has been deposited at CCDC and can be obtained using the deposition number **2173359**.

Crystal Data for $C_{17}H_6AgBF_{18}N_6$ (M =754.96 g/mol): orthorhombic, space group $P2_12_12_1$ (no. 19), a = 8.1157(7) Å, b = 14.9432(14) Å, c = 19.7639(18) Å, V = 2396.9(4) Å^3, Z = 4, T = 99.84 K, μ(Mo Kα) = 1.003 mm^{-1}, $Dcalc$ = 2.092 g/cm^3, 27721 reflections measured (3.416° ≤ 2Θ ≤ 61.016°), 7115 unique (R_{int} = 0.0176, R_{sigma} = 0.0156) which were used in all calculations. The final R_1 was 0.0256 (I > 2σ(I)) and wR_2 was 0.0649 (all data).

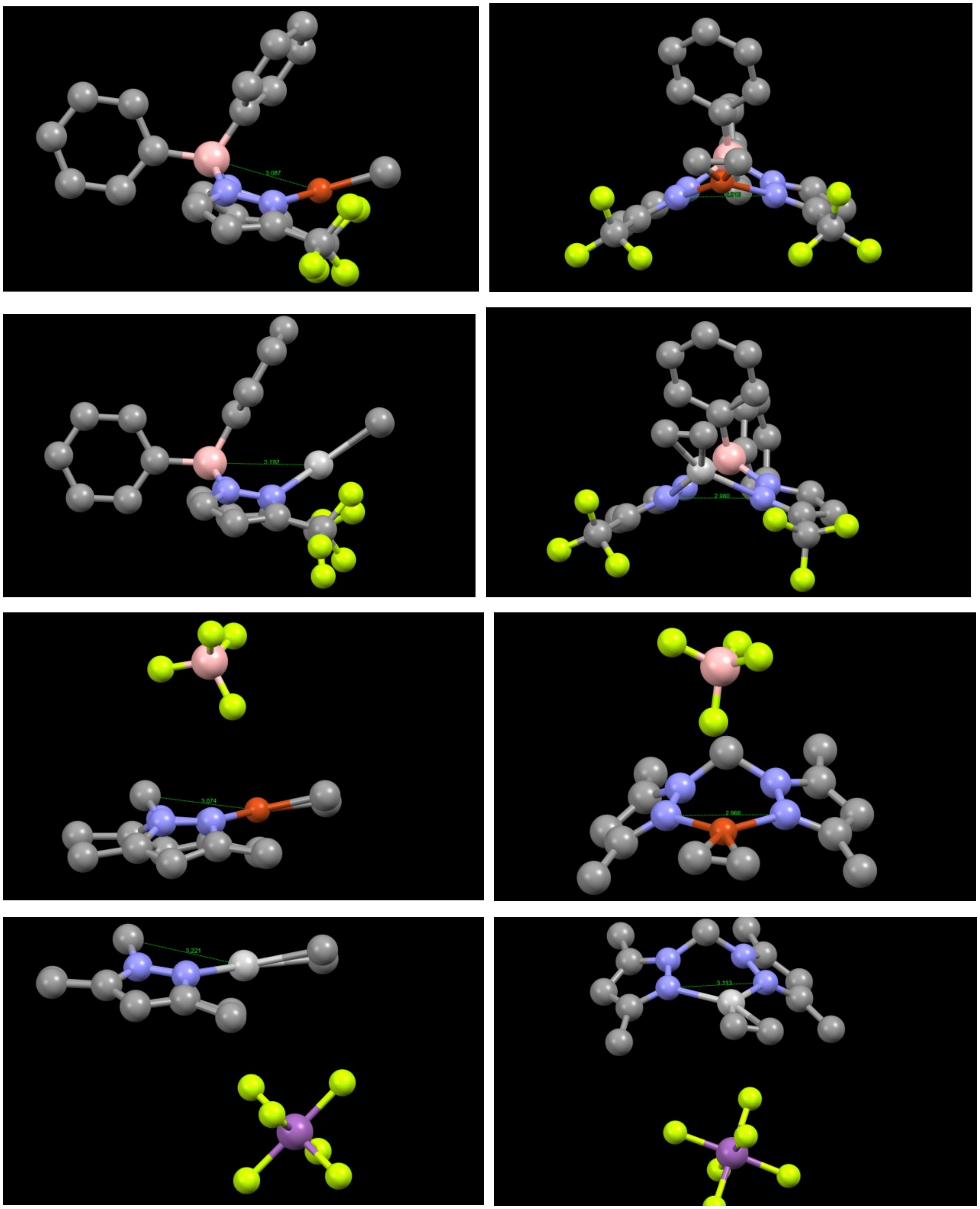

Figure S43: Views illustrating the differences in M(NN)B and M(NN)C cores of compounds [$Ph_2B(3-(CF_3)Pz)_2$]$Ag(C_2H_2)$ (**9**), [$Ph_2B(3-(CF_3)Pz)_2$]$Ag(C_2H_2)$ (**10**), [{$H_2C(3,5-(Me)_2Pz)_2$}$Cu(C_2H_2)$][BF_4] (**11**), and [{$H_2C(3,5-(Me)_2Pz)_2$}$Ag(C_2H_2)$][SbF_6] (**12**) (from top to bottom)

Table S19. The C≡C distance of structurally characterized copper and silver acetylene complexes with η^2-bound terminal acetylene ligands.

Entry	Compound	C≡C (Å) bond length	Reference
1	$[Ph_2B(3\text{-}(CF_3)Pz)_2]Cu(C_2H_2)$ (**9**)	1.217(3)	This work
2	$[HB(3,5\text{-}(CF_3)_2Pz)_3]Cu(C_2H_2)$ (**13**)	1.134(7)	This work
3	$[\{H_2C(3,5\text{-}(CH_3)_2Pz)_2\}Cu(C_2H_2)][BF_4]$ (**11**)	1.203(4)	This work
4	$[H_2B(3,5\text{-}(CF_3)_2Pz)_2]Cu(C_2H_2)$ (**4**)	1.225(5)	[9]
5	$Cu_2(\mu\text{-}[4\text{-Br-}3,5\text{-}(CF_3)_2Pz])_2(C_2H_2)_2$ (**3**)	1.227(4)	[10]
6	$[Cu\{NH(Py)_2\}(C_2H_2)][BF_4]$ (**1**$[BF_4]$)	1.188(11)	[11, 12]
7	$[Cu(phen)(C_2H_2)][ClO_4]$ (**2**$[ClO_4]$)	1.190(7)	[13]
8	$[Ph_2B(3\text{-}(CF_3)Pz)_2]Ag(C_2H_2)$ (**10**)	1.193(3)	This work
9	$[HB(3,5\text{-}(CF_3)_2Pz)_3]Ag(C_2H_2)$(**5**)	1.143(14)	[5]
10	$[\{H_2C(3,5\text{-}(CH_3)_2Pz)_2\}Ag(C_2H_2)][SbF_6]$ (**12**)	1.203(5)	This work
11	$[Al(OC(CH_3)(CF_3)_2)_4]Ag(C_2H_2)$ (**8**)	1.2089(12)	[14]
12	$[Ag(C_2H_2)_3][Al(OC(CF_3)_3)_4]$ (**6**$[Al(OC(CF_3)_3)_4]$)	av. 1.123	[14]
13	$[Ag(C_2H_2)_4][Al(OC(CF_3)_3)_4]$ (**7**$[Al(OC(CF_3)_3)_4]$)	1.092(7)	[14]
14	*Free* C_2H_2	*1.20286(3)*	[15]

Table S20. The analysis of metal coordinated acetylene C≡C bond distance

The C≡C distances (Å) reported in the manuscript (in **bold**) and the effect on resolution (obtained from the refinement of X-ray data using various d values using Shelx SHEL 999 d command (where d represents resolution from Bragg's law, $\lambda = 2d \sin\theta$, for $\lambda = 0.71073$ for Mo radiation) and the TLS distance considering the librational effect (based on librational analysis using TLS -Libr command in Olex2-1.5-dev for all the anisotropic atoms in each of the molecules).

[HB(3,5-$(CF_3)_2$Pz$)_3$]Cu(C_2H_2) (**13**), 100K data							
	Resolution	angle 2θ (deg)	R1	wR2	GOOF	CC distance from X-ray	CC (TLS distance)
**Reported**	**0.67**	**64.1**	**5.34**	**13.60**	**1.038**	**1.134(7)**	1.1344(11)
	0.66	64.7	5.41	14.05	1.053	1.135(7)	
	0.70	61.0	4.93	11.44	1.038	1.126(6)	
	0.77	55.0	4.22	9.49	1.070	1.115(6)	
	0.84	50.0	3.67	8.30	1.099	1.107(6)	1.1096(10)
[Ph_2B(3-(CF_3)Pz$)_2$]Cu(C_2H_2) (**9**), 100K							
	Resolution	angle 2θ (deg)	R1	wR2	GOOF	CC distance from X-ray	CC (TLS distance)
**Reported**	**0.65**	**66.3**	**3.31**	**8.57**	**1.077**	**1.217(3)**	1.219(2)
	0.58	76.3	4.01	10.12	1.073	1.218(3)	
	0.70	61.0	3.08	8.05	1.071	1.215(3)	
	0.77	55.0	2.86	7.24	1.082	1.210(3)	
	0.84	50.0	2.67	6.53	1.064	1.202(4)	1.204(2)
[Ph_2B(3-(CF_3)Pz$)_2$]Ag(C_2H_2) (**10**), 100K							
	Resolution	angle 2θ (deg)	R1	wR2	GOOF	CC distance from X-ray	CC (TLS distance)
**Reported**	**0.60**	**72.6**	**3.54**	**8.12**	**1.053**	**1.193(3)**	1.196(2)
	0.58	76.2	3.88	8.27	1.063	1.193(3)	
	0.70	61.0	2.56	6.46	1.051	1.189(3)	
	0.77	55.0	2.27	5.90	1.039	1.186(3)	
	0.84	50.0	2.08	5.58	1.048	1.182(4)	1.184(2)
[{H_2C(3,5-$(CH_3)_2$Pz$)_2$}Cu(C_2H_2)][BF_4] (**11**), 100K							
	Resolution	angle 2θ (deg)	R1	wR2	GOOF	CC distance from X-ray	CC (TLS distance)
**Reported**	**0.73**	**58.3**	**4.92**	**9.31**	**1.072**	**1.203(4)**	1.2043(9)
	0.67	64.6	6.62	11.92	1.122	1.202(5)	
	0.70	61.0	5.58	10.36	1.098	1.202(4)	
	0.77	55.0	4.22	8.12	1.073	1.202(4)	
	0.84	50.0	3.35	6.79	1.079	1.200(4)	1.2008(7)
[{H_2C(3,5-$(CH_3)_2$Pz$)_2$}Ag(C_2H_2)][SbF_6] (**12**), 100K							
	Resolution	angle 2θ (deg)	R1	wR2	GOOF	CC distance from X-ray	CC (TLS distance)
**Reported**	**0.67**	**64.1**	**3.52**	**7.44**	**1.022**	**1.203(5)**	1.204(7)
	0.53	84.4	5.56	11.10	1.078	1.208(5)	
	0.70	61.0	3.24	6.92	1.019	1.199(5)	
	0.77	55.0	2.70	5.86	1.022	1.198(6)	
	0.84	50.0	2.34	5.10	1.036	1.200(6)	1.201(5)

Computational Details

Geometry optimizations of the complexes were performed without symmetry constraints using the Gaussian09[16] optimizer together with Turbomole 7.1[17] energies and gradients at the BP86[18, 19]/def2-TZVPP[20] level of theory using the D3 dispersion correction suggested by Grimme et al.[21] and the resolution-of-identity (RI) approximation.[22] This level is denoted RI-BP86-D3/def2-TZVPP. Vibrational analysis was performed to ensure that the optimized geometry corresponds to an energy minimum. Natural Bond Orbital (NBO) calculations were carried out using the NBO6.0 program[23] at the BP86-D3/def2-TZVPP level using the optimized the RI-BP86-D3/def2-TZVPP geometries.

The interaction ΔE_{int} between the selected fragments is analyzed with the help of the Energy Decomposition Analysis (EDA) method.[24, 25] Within this approach, ΔE_{int} can be decomposed into the following physically meaningful terms:

$$\Delta E_{int} = \Delta E_{elstat} + \Delta E_{Pauli} + \Delta E_{orb} + \Delta E_{disp}$$

The term ΔE_{elstat} corresponds to the classical electrostatic interaction between the unperturbed charge distributions of the deformed reactants and is usually attractive. The Pauli repulsion ΔE_{Pauli} comprises the destabilizing interactions between occupied orbitals and is responsible for any steric repulsion. The orbital interaction ΔE_{orb} accounts for electron-pair bonding, charge transfer (interaction between occupied orbitals on one moiety with unoccupied orbitals on the other, including HOMO–LUMO interactions), and polarization (empty-occupied orbital mixing on one fragment due to the presence of another fragment). Finally, the ΔE_{disp} term takes into account the interactions which are due to dispersion forces. Moreover, the NOCV (Natural Orbital for Chemical Valence)[26] extension of the EDA method has been also used to further partition the ΔE_{orb} term. The EDA-NOCV approach provides pairwise energy contributions for each pair of interacting orbitals to the total bond energy.

The program package AMS 2020.101[27, 28] was used for the EDA-NOCV calculations at the same BP86-D3 level, in conjunction with a triple- ζ -quality basis set using uncontracted Slater-type orbitals (STOs) augmented by two sets of polarization functions with a frozen-core approximation for the core electrons.[29] Auxiliary sets of s, p, d, f, and g STOs were used to fit the molecular densities and to represent the Coulomb and exchange potentials accurately in each SCF cycle. Scalar relativistic effects were incorporated by applying the zeroth-order regular approximation (ZORA).[30-33] This level of theory is denoted ZORA-BP86-D3/TZ2P//RI-BP86-D3/def2-TZVPP.

Cartesian coordinates (in Å) and total energies (in a. u., ZPVE included) of all the stationary points described in the text (RI-BP86-D3/def2-TZVPP).

$[H_2B(3,5-(CF_3)_2Pz)_2]Cu(C_2H_2)$ **(4):** E= -3544.5019625

Cu	-0.004800000	-1.726243000	0.821695000
F	3.539713000	-2.719976000	0.441640000
F	2.239654000	-2.795030000	-1.315557000
F	4.254074000	-1.938026000	-1.476946000
F	-3.554048000	-2.700859000	0.444817000
F	-4.265813000	-1.916851000	-1.473918000
F	-2.255913000	-2.784516000	-1.313375000
N	1.272436000	0.820315000	0.463923000
N	1.463944000	-0.509654000	0.295831000
N	-1.268170000	0.826845000	0.463278000
N	-1.467228000	-0.502149000	0.296481000
C	2.639453000	-0.667404000	-0.344412000
C	3.234524000	0.572316000	-0.587941000
H	4.177980000	0.779008000	-1.074279000
C	2.331144000	1.489529000	-0.058027000
C	3.165090000	-2.027649000	-0.679421000
C	-2.643485000	-0.653882000	-0.343806000
C	-3.231359000	0.588980000	-0.588877000
H	-4.173463000	0.800568000	-1.075739000
C	-2.322824000	1.501580000	-0.059774000
C	-3.176700000	-2.011591000	-0.677175000
H	0.001439000	0.759231000	2.323249000
H	0.006134000	2.514869000	1.304749000
C	0.614910000	-3.442364000	1.551738000
H	1.637874000	-3.758842000	1.670198000
C	-0.632474000	-3.438586000	1.553679000
H	-1.656890000	-3.748830000	1.675835000
B	0.003193000	1.320450000	1.248889000
C	-2.454726000	2.997932000	-0.031307000
F	-3.625130000	3.358586000	-0.624253000
F	-2.470964000	3.488105000	1.233457000

F	-1.452048000	3.617538000	-0.699480000
C	2.471650000	2.985066000	-0.028216000
F	1.474801000	3.610750000	-0.699559000
F	2.486507000	3.474651000	1.236704000
F	3.646061000	3.339157000	-0.617195000

$[H_2B(3,5\text{-}(CF_3)_2Pz)_2]Ag(C_2H_2)$ **(4-Ag):** E= -2050.7774432

Ag	-1.596112000	-0.018941000	0.643016000
F	-2.370764000	-3.554479000	0.463481000
F	-2.942090000	-2.391355000	-1.295894000
F	-2.119588000	-4.412657000	-1.539886000
F	-2.299486000	3.528507000	0.697662000
F	-2.044653000	4.512674000	-1.246841000
F	-2.905827000	2.496382000	-1.130246000
N	0.983418000	-1.292461000	-0.559770000
N	-0.325543000	-1.537600000	-0.330216000
N	1.002424000	1.276563000	-0.488987000
N	-0.296984000	1.537545000	-0.225291000
C	-0.623792000	-2.714725000	-0.909123000
C	0.507046000	-3.264188000	-1.517617000
H	0.596004000	-4.199604000	-2.052903000
C	1.506528000	-2.326977000	-1.271166000
C	-2.010010000	-3.269309000	-0.826949000
C	-0.579210000	2.747339000	-0.741325000
C	0.553481000	3.302786000	-1.340945000
H	0.654255000	4.261238000	-1.831600000
C	1.536492000	2.334300000	-1.156594000
C	-1.953105000	3.322948000	-0.611634000
H	1.458183000	-0.059951000	1.270982000
H	2.836624000	-0.030559000	-0.209950000
C	-3.339337000	-0.659654000	1.765815000
H	-3.599979000	-1.698506000	1.872185000
C	-3.322945000	0.579708000	1.812598000
H	-3.555467000	1.614298000	1.996953000
B	1.676032000	-0.029670000	0.079026000
C	2.971265000	2.419965000	-1.593937000
F	3.178845000	3.608403000	-2.225345000

F	3.834920000	2.361028000	-0.549556000
F	3.307346000	1.435978000	-2.462263000
C	2.948583000	-2.425626000	-1.681547000
F	3.330917000	-1.407401000	-2.488255000
F	3.787286000	-2.443716000	-0.614879000
F	3.142420000	-3.584854000	-2.369161000

$[H_2B(3,5\text{-}(CH_3)_2Pz)_2]Cu(C_2H_2)$ **(4'):** E= -3544.5019625

Cu	-0.000768000	-1.462810000	-0.378043000
N	-1.261765000	1.104761000	-0.389756000
N	-1.462353000	-0.203322000	-0.044964000
N	1.262974000	1.103484000	-0.390073000
N	1.461905000	-0.204670000	-0.044570000
C	-2.619828000	-0.278261000	0.642469000
C	-3.177140000	1.005897000	0.735479000
H	-4.104503000	1.286689000	1.222029000
C	-2.288578000	1.857246000	0.071721000
C	2.619247000	-0.280670000	0.642964000
C	3.178142000	1.002843000	0.735340000
H	4.105843000	1.282718000	1.221759000
C	2.290630000	1.854960000	0.071167000
H	0.000172000	0.797148000	-2.236914000
H	0.001302000	2.666020000	-1.457276000
C	-0.628627000	-3.252265000	-0.822590000
H	-1.626187000	-3.645105000	-0.933194000
C	0.626147000	-3.252657000	-0.822458000
H	1.623509000	-3.645721000	-0.933779000
B	0.000704000	1.480919000	-1.228159000
C	-2.369798000	3.332936000	-0.141604000
H	-2.414919000	3.582060000	-1.210502000
H	-3.266685000	3.731583000	0.346735000
H	-1.490758000	3.846134000	0.271424000
C	-3.135099000	-1.581946000	1.158830000
H	-3.812672000	-2.060571000	0.435258000
H	-2.305351000	-2.275018000	1.351211000
H	-3.693573000	-1.441078000	2.092448000
C	2.373690000	3.330436000	-0.142878000

H	3.270516000	3.728359000	0.346138000
H	2.420176000	3.578860000	-1.211893000
H	1.494782000	3.844906000	0.268850000
C	3.132709000	-1.584660000	1.160363000
H	3.688221000	-1.444335000	2.095834000
H	2.302201000	-2.277571000	1.349933000
H	3.812388000	-2.063128000	0.438693000

$[H_2B(3,5\text{-}(CH_3)_2Pz)_2]Ag(C_2H_2)$ **(4-Ag'):** E= -2050.7774432

Ag	-1.493240000	-0.029752000	-0.307157000
N	1.264306000	1.285411000	-0.432338000
N	-0.006847000	1.534209000	0.002889000
N	1.305951000	-1.246483000	-0.435028000
N	0.041087000	-1.545688000	-0.012534000
C	0.018899000	2.663305000	0.737118000
C	1.334128000	3.155780000	0.769605000
H	1.690537000	4.050748000	1.267543000
C	2.096064000	2.255463000	0.021589000
C	0.104603000	-2.672913000	0.722402000
C	1.437330000	-3.114606000	0.765504000
H	1.823129000	-3.997540000	1.262970000
C	2.170446000	-2.184223000	0.025458000
H	0.747303000	0.011939000	-2.230155000
H	2.692763000	0.044044000	-1.687796000
C	-3.506908000	0.590446000	-0.686585000
H	-3.877139000	1.599035000	-0.758816000
C	-3.507007000	-0.657868000	-0.699983000
H	-3.883227000	-1.662276000	-0.795669000
B	1.546439000	0.024268000	-1.309622000
C	-1.218868000	3.224645000	1.357759000
H	-1.533081000	4.152085000	0.856383000
H	-2.045755000	2.505255000	1.292207000
H	-1.058176000	3.462439000	2.417999000
C	3.559310000	2.274172000	-0.276584000
H	3.749005000	2.325454000	-1.357125000
H	4.025929000	3.143439000	0.201528000
H	4.055857000	1.366746000	0.093883000

C	-1.111133000	-3.260704000	1.360855000
H	-1.108710000	-3.102172000	2.449366000
H	-2.020781000	-2.796342000	0.957902000
H	-1.167499000	-4.343419000	1.185272000
C	3.634864000	-2.149372000	-0.265351000
H	3.831754000	-2.196235000	-1.344885000
H	4.095392000	-1.223316000	0.105239000
H	4.130940000	-2.999779000	0.217026000

$[Ph_2B(3\text{-}(CF_3)Pz)_2]Cu(C_2H_2)$ **(9):** E= -3332.2638014

Cu	-1.487873000	-0.000450000	0.424966000
F	-2.553816000	-3.557254000	0.340814000
F	-3.125901000	-2.267769000	-1.330010000
F	-2.342054000	-4.282041000	-1.716052000
F	-2.554702000	3.558152000	0.341709000
F	-2.343890000	4.281045000	-1.715976000
F	-3.126624000	2.266817000	-1.327693000
N	0.832219000	-1.272394000	-0.666615000
N	-0.500633000	-1.451473000	-0.481168000
N	0.831855000	1.273244000	-0.665673000
N	-0.501274000	1.451402000	-0.481120000
C	-0.807296000	-2.656516000	-0.994986000
C	0.337726000	-3.278909000	-1.507653000
H	0.405055000	-4.249995000	-1.980486000
C	1.358720000	-2.362955000	-1.272518000
H	2.418628000	-2.411586000	-1.489048000
C	-2.203971000	-3.184479000	-0.932237000
C	-0.808119000	2.656926000	-0.993735000
C	0.337012000	3.280579000	-1.504588000
H	0.404238000	4.252327000	-1.976075000
C	1.358287000	2.364878000	-1.269723000
H	2.418347000	2.414373000	-1.485286000
C	-2.205023000	3.184320000	-0.931123000
C	1.222403000	-0.000365000	1.557833000
C	1.093326000	1.199744000	2.281384000
H	1.183532000	2.152917000	1.756253000
C	0.830665000	1.205322000	3.653231000

H	0.726430000	2.152902000	4.183560000
C	0.694558000	-0.001719000	4.343964000
H	0.480926000	-0.002248000	5.413244000
C	0.832440000	-1.208108000	3.652381000
H	0.729465000	-2.156187000	4.182062000
C	1.095038000	-1.201205000	2.280559000
H	1.186547000	-2.153873000	1.754741000
C	3.108204000	0.000656000	-0.422407000
C	3.529102000	0.001812000	-1.768752000
H	2.779523000	0.002573000	-2.564197000
C	4.881678000	0.002134000	-2.117140000
H	5.175099000	0.003177000	-3.167906000
C	5.858400000	0.001179000	-1.116226000
H	6.916128000	0.001578000	-1.381676000
C	5.468119000	-0.000095000	0.224749000
H	6.223060000	-0.000682000	1.012166000
C	4.110503000	-0.000283000	0.561683000
H	3.819581000	-0.001517000	1.613216000
C	-2.835452000	-0.624352000	1.706445000
H	-3.082830000	-1.646994000	1.936477000
C	-2.836955000	0.623552000	1.704646000
H	-3.084731000	1.646123000	1.934616000
B	1.544169000	0.000299000	-0.030699000

$[Ph_2B(3\text{-}(CF_3)Pz)_2]Ag(C_2H_2)$ **(10):** E= -1838.5381869

Ag	1.617344000	0.014372000	0.394462000
F	1.988793000	4.455846000	-1.767382000
F	2.855194000	2.448538000	-1.560134000
F	2.341651000	3.602223000	0.222328000
F	2.888701000	-2.411869000	-1.569171000
F	2.415533000	-3.551907000	0.233909000
F	2.060474000	-4.438948000	-1.740254000
N	-1.007549000	1.272008000	-0.687364000
N	0.318726000	1.514452000	-0.554485000
N	-0.988864000	-1.300834000	-0.674687000
N	0.343004000	-1.514757000	-0.550252000
C	0.548948000	2.732349000	-1.072308000

C	-0.640475000	3.303093000	-1.542677000
H	-0.772073000	4.272154000	-2.005797000
C	-1.607026000	2.340081000	-1.271387000
H	-2.675451000	2.343336000	-1.445623000
C	1.927471000	3.306638000	-1.052506000
C	0.593496000	-2.734601000	-1.054130000
C	-0.589183000	-3.337989000	-1.500486000
H	-0.705567000	-4.315892000	-1.949095000
C	-1.572486000	-2.390450000	-1.234713000
H	-2.642221000	-2.418153000	-1.398575000
C	1.983219000	-3.281322000	-1.040866000
C	-1.287840000	-0.005004000	1.558683000
C	-1.175126000	1.199705000	2.277369000
H	-1.304622000	2.149024000	1.753640000
C	-0.885505000	1.215685000	3.643966000
H	-0.796340000	2.167139000	4.169931000
C	-0.704177000	0.014741000	4.334925000
H	-0.470300000	0.022618000	5.399865000
C	-0.823830000	-1.196332000	3.648293000
H	-0.685498000	-2.140155000	4.177419000
C	-1.113425000	-1.200001000	2.281477000
H	-1.193867000	-2.156788000	1.761472000
C	-3.247700000	-0.026959000	-0.336258000
C	-4.204154000	-0.010477000	0.692118000
H	-3.866005000	0.006434000	1.729189000
C	-5.576020000	-0.014481000	0.418161000
H	-6.293504000	-0.001224000	1.239605000
C	-6.028107000	-0.036112000	-0.902843000
H	-7.096821000	-0.039481000	-1.119225000
C	-5.098321000	-0.052974000	-1.947358000
H	-5.439496000	-0.069240000	-2.983364000
C	-3.731452000	-0.047512000	-1.661451000
H	-3.019600000	-0.059854000	-2.490618000
C	3.313212000	0.638048000	1.576067000
H	3.549653000	1.670868000	1.763346000
C	3.334773000	-0.603066000	1.545698000
H	3.606336000	-1.635386000	1.681448000
B	-1.663569000	-0.016056000	-0.017628000

$[\{H_2C(3,5\text{-}(CH_3)_2Pz)_2\}Cu(C_2H_2)]^+$ **(11+):** E= -2365.8999517

Cu	-0.003152000	-1.557138000	-0.340753000
N	-1.198930000	1.079794000	-0.406633000
N	1.203495000	1.074764000	-0.406586000
N	-1.451664000	-0.229677000	-0.090541000
N	1.450929000	-0.235778000	-0.090580000
C	-0.632370000	-3.381996000	-0.700410000
C	-3.205436000	-1.498189000	1.111905000
H	-3.936467000	-1.901636000	0.396450000
H	-3.729722000	-1.324153000	2.058434000
H	-2.430262000	-2.256602000	1.276428000
C	0.616285000	-3.385292000	-0.700932000
C	-2.607196000	-0.232322000	0.600237000
C	-2.174945000	1.907864000	0.071442000
C	2.606536000	-0.243155000	0.600037000
C	2.183002000	1.898800000	0.071364000
C	0.002957000	1.385369000	-1.155391000
H	0.005198000	2.448110000	-1.409684000
H	0.001704000	0.791419000	-2.080534000
C	3.094503000	1.074257000	0.715897000
H	4.004951000	1.389891000	1.211445000
C	-3.089713000	1.087060000	0.716194000
H	-3.998815000	1.406427000	1.211820000
C	3.199593000	-1.511344000	1.111926000
H	2.422210000	-2.268966000	1.269409000
H	3.718040000	-1.340755000	2.062329000
H	3.934693000	-1.913834000	0.400122000
C	-2.161791000	3.384469000	-0.130297000
H	-1.265939000	3.848147000	0.306898000
H	-3.036347000	3.829285000	0.354924000
H	-2.199371000	3.652131000	-1.196113000
C	2.175923000	3.375439000	-0.130427000
H	2.216263000	3.642901000	-1.196199000
H	3.051432000	3.816853000	0.356177000
H	1.281196000	3.842640000	0.305303000
H	-1.644570000	-3.742573000	-0.786457000

H	1.626314000	-3.751750000	-0.787790000

[{$H_2C(3,5\text{-}(CH_3)_2Pz)_2$}$Ag(C_2H_2)$]$^+$ **(12+):** E= -872.1749758

Ag	-1.581016000	-0.007830000	-0.261946000
N	1.310911000	-1.197538000	-0.417181000
N	0.031474000	-1.521061000	-0.051515000
N	1.292776000	1.217888000	-0.418070000
N	0.005851000	1.533270000	-0.070748000
C	0.118878000	-2.668570000	0.643204000
C	1.467003000	-3.080089000	0.711243000
H	1.854081000	-3.970107000	1.193245000
C	2.209710000	-2.126787000	0.032157000
C	-1.086437000	-3.338524000	1.209626000
H	-0.940109000	-3.568165000	2.272792000
H	-1.284128000	-4.287477000	0.692194000
H	-1.973630000	-2.700944000	1.115402000
C	3.674938000	-2.043997000	-0.228844000
H	3.900830000	-2.037484000	-1.304847000
H	4.174544000	-2.913765000	0.209595000
H	4.120316000	-1.144159000	0.218220000
C	0.076484000	2.679745000	0.627572000
C	1.421011000	3.099586000	0.715491000
H	1.795402000	3.992090000	1.202948000
C	2.179151000	2.151915000	0.045962000
C	-1.139484000	3.334346000	1.188509000
H	-2.044245000	2.771533000	0.929966000
H	-1.243540000	4.357763000	0.804340000
H	-1.077461000	3.398507000	2.283134000
C	3.648279000	2.078626000	-0.194709000
H	4.093291000	1.183745000	0.262556000
H	4.135745000	2.953608000	0.246978000
H	3.889267000	2.069288000	-1.267419000
C	1.539555000	0.012049000	-1.181902000
H	0.877394000	0.007440000	-2.058849000
H	2.577919000	0.019114000	-1.523219000
C	-3.635508000	-0.660842000	-0.533812000
H	-3.942137000	-1.692186000	-0.578502000

C	-3.656091000	0.579627000	-0.546592000
H	-3.997808000	1.598766000	-0.611894000

[HB(3,5-$(CF_3)_2Pz)_3$]Cu(C_2H_2) **(13):** E= -4444.0383382

Cu	-1.865345000	0.037413000	-0.100050000
F	3.565665000	-1.502406000	1.839327000
F	-2.207347000	-3.631401000	-3.331386000
F	3.073692000	-3.101200000	-2.839253000
F	-3.063536000	-1.708822000	-2.715094000
F	3.402094000	-0.824684000	3.915659000
F	-2.872950000	-3.335863000	-1.264713000
F	3.340103000	-0.966551000	-2.430615000
F	-2.717641000	-1.741632000	3.084052000
F	3.407548000	-2.410628000	-0.788587000
F	-1.850082000	-1.019184000	4.963993000
N	-0.676342000	-1.257230000	-1.101468000
N	0.804223000	-0.293448000	1.417745000
N	-0.519813000	-0.345383000	1.692299000
N	0.668041000	-1.094356000	-0.991629000
C	0.339404000	-2.765919000	-2.441497000
H	0.504066000	-3.566752000	-3.149215000
C	-0.877710000	-2.260651000	-1.970814000
C	-0.631583000	-0.613100000	2.998937000
C	0.629779000	-0.741168000	3.599950000
H	0.864798000	-0.955015000	4.633827000
C	1.297610000	-1.996945000	-1.797570000
C	-2.257721000	-2.727904000	-2.323492000
C	1.521075000	-0.531008000	2.558331000
C	3.018511000	-0.553187000	2.640625000
C	2.787331000	-2.109981000	-1.955023000
C	-1.980383000	-0.742584000	3.640840000
C	-3.714428000	-0.651405000	0.233320000
H	-4.016442000	-1.676071000	0.108282000
B	1.260121000	0.001839000	-0.040442000
H	2.448840000	0.009040000	-0.116682000
F	3.572307000	0.636476000	2.293327000
F	-2.144346000	4.671937000	-1.666201000

F	3.126697000	3.929901000	-1.392362000
F	-3.026809000	2.672181000	-1.842197000
F	-2.828975000	3.610955000	0.124444000
F	3.368820000	1.803557000	-1.855917000
F	-2.711045000	0.399775000	3.531081000
F	3.435674000	2.483792000	0.222102000
N	-0.655644000	1.601471000	-0.520774000
N	0.686081000	1.391027000	-0.483739000
C	0.385190000	3.497082000	-1.174581000
H	0.563189000	4.507296000	-1.516710000
C	-0.840424000	2.864992000	-0.936862000
C	1.330764000	2.527035000	-0.876236000
C	-2.212627000	3.450089000	-1.085301000
C	2.822938000	2.674260000	-0.970963000
C	-3.708224000	0.565832000	0.474069000
H	-4.001714000	1.564311000	0.745756000

[HB(3,5-$(CF_3)_2$Pz$)_3$]Ag(C_2H_2) **(5):** E= -2950.3134672

Ag	-2.002855000	-0.073707000	-0.220147000
F	3.261825000	-2.106579000	1.510180000
F	-1.588488000	-4.156135000	-3.011929000
F	3.444541000	-2.194965000	-3.298546000
F	-2.660797000	-2.260129000	-2.757070000
F	3.354112000	-1.733654000	3.668859000
F	-2.333543000	-3.561945000	-1.033247000
F	3.235142000	-0.115310000	-2.635141000
F	-2.703533000	-1.527128000	3.212728000
F	3.906639000	-1.652758000	-1.230927000
F	-1.816950000	-0.656533000	5.018070000
N	-0.374013000	-1.449834000	-0.989191000
N	0.874222000	-0.261940000	1.446812000
N	-0.424749000	-0.083039000	1.773235000
N	0.904365000	-1.005429000	-0.987530000
C	0.787442000	-2.613741000	-2.539617000
H	1.043625000	-3.314241000	-3.322803000
C	-0.449927000	-2.418323000	-1.913989000
C	-0.566095000	-0.519158000	3.029684000

C	0.649575000	-0.995569000	3.546636000
H	0.854963000	-1.402950000	4.527498000
C	1.622483000	-1.691185000	-1.923061000
C	-1.756050000	-3.102241000	-2.180089000
C	1.545526000	-0.814312000	2.503165000
C	2.997095000	-1.185949000	2.478112000
C	3.060139000	-1.409998000	-2.258216000
C	-1.918375000	-0.506800000	3.672969000
C	-4.037685000	-0.842415000	0.118060000
H	-4.214041000	-1.903020000	0.107060000
B	1.372500000	0.097666000	0.019506000
H	2.561820000	0.162679000	0.000877000
F	3.811835000	-0.124523000	2.256722000
F	-2.066119000	4.620743000	-1.912418000
F	3.088281000	4.264006000	-0.270145000
F	-2.735034000	2.559948000	-2.272158000
F	-2.937897000	3.442440000	-0.282708000
F	3.662726000	2.285150000	-1.006991000
F	-2.593408000	0.645407000	3.427764000
F	3.122999000	2.550470000	1.097196000
N	-0.543872000	1.600804000	-0.723040000
N	0.765162000	1.472992000	-0.404977000
C	0.432324000	3.632734000	-0.886476000
H	0.587678000	4.689902000	-1.053726000
C	-0.749406000	2.892353000	-1.017937000
C	1.373027000	2.691057000	-0.493011000
C	-2.119209000	3.377829000	-1.379579000
C	2.819387000	2.936095000	-0.169518000
C	-4.123925000	0.387978000	0.178299000
H	-4.439118000	1.411987000	0.268518000

$[HB(3-(CF_3),5-(Ph)Pz)_3]Cu(C_2H_2)$ **(14):** E= -4125.6687580

Cu	-2.458821000	0.107041000	0.118693000
F	-2.655251000	4.911487000	-1.008356000
F	-3.588791000	3.338039000	0.201563000
F	-3.368841000	3.068644000	-1.957083000
F	-3.336358000	-1.562031000	-3.274363000

F	-2.423688000	-3.503954000	-3.729623000
N	-1.252213000	1.666735000	-0.323667000
N	0.176115000	-0.899847000	-1.165222000
N	-1.144781000	-1.143573000	-1.336605000
N	0.084004000	1.447969000	-0.202401000
C	-0.150428000	3.599074000	-0.726650000
H	0.047744000	4.642810000	-0.932955000
C	-1.395837000	2.965727000	-0.635723000
C	-1.229121000	-2.068500000	-2.301253000
C	0.043275000	-2.443100000	-2.761758000
H	0.286494000	-3.145590000	-3.548677000
C	0.782463000	2.602512000	-0.432179000
C	-2.750021000	3.567560000	-0.842685000
C	0.932982000	-1.667923000	-2.016130000
C	-2.566211000	-2.560353000	-2.758840000
C	-4.303079000	0.275400000	-0.623779000
H	-4.610056000	0.999776000	-1.357462000
B	0.625718000	0.006111000	0.000401000
H	1.810505000	0.039067000	0.045299000
F	-2.649482000	-1.360293000	4.814505000
F	-3.525042000	-0.006110000	3.326927000
F	-3.443554000	-2.154665000	2.931148000
F	-3.282532000	-3.125705000	-1.746907000
N	-1.251971000	-0.561865000	1.594614000
N	0.083602000	-0.550070000	1.345737000
C	-0.149292000	-1.190903000	3.465390000
H	0.049987000	-1.535787000	4.471641000
C	-1.394883000	-0.952186000	2.872403000
C	0.783168000	-0.930243000	2.459068000
C	-2.750665000	-1.111427000	3.483759000
C	-4.299766000	-0.657330000	0.196835000
H	-4.600212000	-1.477499000	0.824777000
C	2.249861000	-1.011143000	2.504660000
C	2.977927000	-1.670065000	1.499200000
C	2.947403000	-0.402945000	3.561969000
C	4.370736000	-1.689965000	1.531474000
C	4.342577000	-0.436686000	3.600370000
C	5.057444000	-1.071319000	2.580325000

H	2.451788000	-2.164270000	0.684167000
H	2.388421000	0.117689000	4.340122000
H	4.913747000	-2.185829000	0.727613000
H	4.871687000	0.044806000	4.422937000
H	6.147209000	-1.085047000	2.603975000
C	2.400382000	-1.629102000	-2.074219000
C	3.126746000	-2.832851000	-2.099047000
C	3.105983000	-0.413339000	-2.102075000
C	4.522079000	-2.819392000	-2.135483000
C	4.499437000	-0.401723000	-2.122529000
C	5.212817000	-1.603813000	-2.139401000
H	2.587448000	-3.779986000	-2.067999000
H	2.561797000	0.529361000	-2.108473000
H	5.070571000	-3.761465000	-2.150215000
H	5.022322000	0.554054000	-2.122684000
H	6.302788000	-1.594085000	-2.155152000
C	2.247046000	2.696871000	-0.352201000
C	2.962050000	3.324327000	-1.386277000
C	2.956526000	2.162742000	0.737723000
C	4.355567000	3.397274000	-1.339746000
C	4.347808000	2.222782000	0.774670000
C	5.052034000	2.838811000	-0.264210000
H	2.418698000	3.733543000	-2.238452000
H	2.417075000	1.693075000	1.558507000
H	4.898120000	3.879759000	-2.152804000
H	4.876770000	1.779920000	1.618060000
H	6.140813000	2.884152000	-0.235787000

[HB(3-(CF_3),5-(Ph)Pz)$_3$]Ag(C_2H_2) **(15):** E= -2631.9431741

Ag	2.608711000	0.041555000	-0.190196000
F	2.395746000	4.844706000	-1.623634000
F	3.329110000	2.908703000	-2.064995000
F	3.191411000	3.602296000	0.001712000
F	3.205290000	0.415639000	3.643060000
F	2.267047000	-0.951206000	5.080621000
N	1.087147000	1.654098000	-0.611452000
N	-0.287994000	-0.119661000	1.481935000

N	1.022382000	-0.273262000	1.781894000
N	-0.230028000	1.333251000	-0.619938000
C	-0.085855000	3.433822000	-1.358186000
H	-0.326307000	4.409346000	-1.760471000
C	1.178844000	2.914971000	-1.056573000
C	1.082028000	-0.498805000	3.098082000
C	-0.197738000	-0.503010000	3.675451000
H	-0.460502000	-0.631784000	4.717687000
C	-0.973704000	2.391860000	-1.078968000
C	2.518416000	3.566153000	-1.188345000
C	-1.064721000	-0.248175000	2.611664000
C	2.412448000	-0.689007000	3.753914000
C	4.665896000	0.601308000	0.324627000
H	4.913724000	1.624408000	0.543494000
B	-0.735777000	0.000021000	0.006053000
H	-1.922360000	-0.000495000	-0.035186000
F	2.428221000	-3.946631000	-3.218994000
F	3.255330000	-1.928377000	-2.974867000
F	3.317747000	-3.338706000	-1.307567000
F	3.114194000	-1.722958000	3.208279000
N	1.100323000	-1.385181000	-1.075171000
N	-0.220744000	-1.211545000	-0.825356000
C	-0.062190000	-2.959223000	-2.203173000
H	-0.297638000	-3.817604000	-2.818841000
C	1.200682000	-2.437078000	-1.900105000
C	-0.958184000	-2.157300000	-1.492248000
C	2.545035000	-2.909571000	-2.352713000
C	4.678582000	-0.618476000	0.121588000
H	4.948145000	-1.651779000	-0.003270000
C	-2.423417000	-2.253926000	-1.434407000
C	-3.116926000	-2.214112000	-0.213013000
C	-3.155169000	-2.389140000	-2.626797000
C	-4.509007000	-2.270941000	-0.186953000
C	-4.548763000	-2.463134000	-2.597620000
C	-5.229503000	-2.394452000	-1.378492000
H	-2.564308000	-2.135522000	0.721492000
H	-2.623607000	-2.412820000	-3.578480000
H	-5.025066000	-2.211575000	0.770656000

H	-5.103718000	-2.562172000	-3.530735000
H	-6.318610000	-2.437468000	-1.357375000
C	-2.528313000	-0.125512000	2.632735000
C	-3.294461000	-1.072812000	3.334874000
C	-3.191989000	0.920628000	1.968819000
C	-4.687160000	-0.983677000	3.358069000
C	-4.583448000	0.997955000	1.977062000
C	-5.336521000	0.046656000	2.671306000
H	-2.788388000	-1.892949000	3.844794000
H	-2.615319000	1.679633000	1.443567000
H	-5.266587000	-1.728895000	3.903571000
H	-5.074011000	1.806017000	1.435201000
H	-6.424999000	0.107571000	2.677460000
C	-2.434083000	2.369391000	-1.238562000
C	-3.200406000	3.452126000	-0.774084000
C	-3.089819000	1.288405000	-1.852766000
C	-4.590072000	3.444181000	-0.904961000
C	-4.478414000	1.274076000	-1.966398000
C	-5.233389000	2.351029000	-1.492645000
H	-2.699799000	4.290058000	-0.288479000
H	-2.510289000	0.452859000	-2.241399000
H	-5.171991000	4.288048000	-0.533985000
H	-4.965520000	0.413039000	-2.423045000
H	-6.319946000	2.338547000	-1.580747000

References

1. A. Noonikara-Poyil, A. Muñoz-Castro, A. Boretskyi, P. K. Mykhailiuk and H. V. R. Dias, *Chem. Sci.*, 2021, **12**, 14618-14623.
2. H. V. R. Dias, H.-L. Lu, H.-J. Kim, S. A. Polach, T. K. H. H. Goh, R. G. Browning and C. J. Lovely, *Organometallics*, 2002, **21**, 1466-1473.
3. J. Mehara, B. T. Watson, A. Noonikara-Poyil, A. O. Zacharias, J. Roithová and H. V. R. Dias, *Chem. Eur. J*, 2022, **28**.
4. H. V. R. Dias and T. K. H. H. Goh, *Polyhedron*, 2004, **23**, 273-282.
5. H. V. R. Dias, Z. Wang and W. Jin, *Inorg. Chem.*, 1997, **36**, 6205-6215.
6. G. Sheldrick, *Acta Crystallogr. Sect. A: Found. Adv.*, 2015, **71**, 3-8.
7. G. Sheldrick, *Acta Crystallogr. Sect. C: Struct. Chem.*, 2015, **71**, 3-8.
8. O. V. Dolomanov, L. J. Bourhis, R. J. Gildea, J. A. K. Howard and H. Puschmann, *J. Appl. Cryst.*, 2009, **42**, 339-341.
9. A. Noonikara-Poyil, A. Muñoz-Castro and H. V. R. Dias, *Molecules*, 2021, **27**, 16.
10. D. Parasar, T. T. Ponduru, A. Noonikara-Poyil, N. B. Jayaratna and H. V. R. Dias, *Dalton Trans.*, 2019, **48**, 15782-15794.
11. J. S. Thompson and J. F. Whitney, *Inorg. Chem.*, 2002, **23**, 2813-2819.
12. J. S. Thompson and J. F. Whitney, *J. Am. Chem. Soc.*, 2002, **105**, 5488-5490.
13. M. Munakata, S. Kitagawa, I. Kawada, M. Maekawa and H. Shimono, *J. Chem. Soc., Dalton Trans.*, 1992, DOI: 10.1039/dt9920002225, 2225.
14. A. Reisinger, N. Trapp, I. Krossing, S. Altmannshofer, V. Herz, M. Presnitz and W. Scherer, *Angew. Chem. Int. Ed.*, 2007, **46**, 8295-8298.
15. J. Liévin, J. Demaison, M. Herman, A. Fayt and C. Puzzarini, *J. Chem. Phys*, 2011, **134**, 064119.
16. M. J. Frisch, G. W. Trucks, H. B. Schlegel, G. E. Scuseria, M. A. Robb, J. R. Cheeseman, G. Scalmani, V. Barone, G. A. Petersson, H. Nakatsuji, X. Li, M. Caricato, A. V. Marenich, J. Bloino, B. G. Janesko, R. Gomperts, B. Mennucci, H. P. Hratchian, J. V. Ortiz, A. F. Izmaylov, J. L. Sonnenberg, Williams, F. Ding, F. Lipparini, F. Egidi, J. Goings, B. Peng, A. Petrone, T. Henderson, D. Ranasinghe, V. G. Zakrzewski, J. Gao, N. Rega, G. Zheng, W. Liang, M. Hada, M. Ehara, K. Toyota, R. Fukuda, J. Hasegawa, M. Ishida, T. Nakajima, Y. Honda, O. Kitao, H. Nakai, T. Vreven, K. Throssell, J. A. Montgomery Jr., J. E. Peralta, F. Ogliaro, M. J. Bearpark, J. J. Heyd, E. N. Brothers, K. N. Kudin, V. N. Staroverov, T. A. Keith, R. Kobayashi, J. Normand, K. Raghavachari, A. P. Rendell, J. C. Burant, S. S. Iyengar, J. Tomasi, M. Cossi, J. M. Millam, M. Klene, C. Adamo, R. Cammi, J. W. Ochterski, R. L. Martin, K. Morokuma, O. Farkas, J. B. Foresman and D. J. Fox, *Gaussian 09 Revision A. 1 ed.*, Wallingford, CT, 2009.
17. R. Ahlrichs, M. Bär, M. Häser, H. Horn and C. Kölmel, *Chem. Phys. Lett.*, 1989, **162**, 165-169.
18. A. D. Becke, *Phys. Rev. A*, 1988, **38**, 3098-3100.
19. J. P. Perdew, *Phys. Rev. B*, 1986, **33**, 8822-8824.
20. F. Weigend and R. Ahlrichs, *Phys. Chem. Chem. Phys*, 2005, **7**, 3297.
21. S. Grimme, J. Antony, S. Ehrlich and H. Krieg, *J. Chem. Phys*, 2010, **132**, 154104.
22. K. Eichkorn, O. Treutler, H. Öhm, M. Häser and R. Ahlrichs, *Chem. Phys. Lett.*, 1995, **242**, 652-660.
23. E. D. Glendening, C. R. Landis and F. Weinhold, *J. Comput. Chem.*, 2013, **34**, 1429-1437.

24. M. v. Hopffgarten and G. Frenking, *WIREs Comput. Mol. Sci.*, 2011, **2**, 43-62.
25. F. M. Bickelhaupt and E. J. Baerends, *Kohn-Sham Density Functional Theory: Predicting and Understanding Chemistry*, 2007.
26. M. P. Mitoraj, A. Michalak and T. Ziegler, *J. Chem. Theory Comput.*, 2009, **5**, 962-975.
27. G. te Velde, F. M. Bickelhaupt, E. J. Baerends, C. Fonseca Guerra, S. J. A. van Gisbergen, J. G. Snijders and T. Ziegler, *J. Comput. Chem.*, 2001, **22**, 931-967.
28. ADF2020, *SCM, Theoretical Chemistry*, Vrije Universiteit, Amsterdam, The Netherlands, http://www.scm.com.
29. J. G. Snijders, P. Vernooijs and E. J. Baerends, *At. Data Nucl. Data Tables*, 1981, **26**, 483-509.
30. E. v. Lenthe, E. J. Baerends and J. G. Snijders, *J. Chem. Phys*, 1993, **99**, 4597-4610.
31. E. van Lenthe, E. J. Baerends and J. G. Snijders, *J. Chem. Phys*, 1994, **101**, 9783-9792.
32. E. van Lenthe, A. Ehlers and E.-J. Baerends, *J. Chem. Phys*, 1999, **110**, 8943-8953.
33. J. Krijn and E. J. Baerends, *Fit Functions in the HFS-Method, Internal Report (in Dutch)*, Vrije Universiteit Amsterdam, The Netherlands, 1984.
